# F16 Hybrids Derived from Steviol or Isosteviol Are Accumulated in the Mitochondria of Tumor Cells and Overcome Drug Resistance

**DOI:** 10.3390/molecules29020381

**Published:** 2024-01-12

**Authors:** Niels V. Heise, Julia Heisig, Kristof Meier, René Csuk, Thomas Mueller

**Affiliations:** 1 Organic Chemistry, Martin-Luther University Halle-Wittenberg, Kurt-Mothes, Str. 2, D-06120 Halle (Saale), Germany; niels.heise@chemie.uni-halle.de (N.V.H.); julia.heisig@chemie.uni-halle.de (J.H.); 2 Hematology/Oncology, Medical Faculty, University Clinic for Internal Medicine IV, Martin-Luther University Halle-Wittenberg, Ernst-Grube Str. 40, D-06120 Halle (Saale), Germany; kristof.meier@uk-halle.de (K.M.); thomas.mueller@medizin.uni-halle.de (T.M.)

**Keywords:** steviol, isosteviol, F16 hybrids, cytotoxicity, mitochondrial accumulation

## Abstract

Steviol and isosteviol were prepared from the commercially available sweetener stevioside and converted into lipophilic F16 hybrids. Their cytotoxicity was determined in SRB assays and showed to depend on both the substitution pattern of the aromatic substituent as well as on the spacer length. Therefore, compound **25** held an IC_50_ (A2780) of 180 nM, thus surpassing the activity of comparable rhodamine hybrids. Several of the compounds were also able to overcome drug resistance in the A2780/A2780cis model. Extra staining experiments showed a similar subcellular accumulation pattern of the F16 hybrids as a well-established mitocan, hence proving preferential mitochondrial accumulation but also some other accumulation in other cellular areas.

## 1. Introduction

Exploiting structural metabolic differences between “normal” cells and cancer cells determines the efficiency, safety and success of a therapy and cure for cancer. Since cancer is still one of the most common causes of death worldwide, despite all the successes achieved, the development of new therapeutics is of the highest interest and essential importance. In addition to other structural differences, cancer cells can differ from normal cells in the potential of their mitochondrial membranes [1]. This is higher in many tumor cells, resulting in an increased affinity for cations, especially if these also have a high lipophilicity.

The resulting concept was successfully proven for compounds carrying, for example, a triphenylphosphonium residue [2], or a rhodamine residue connected via a suitable linker [3] or a Changsha NIR ligand [4]. Hybrids of acetylated pentacyclic triterpene carboxylic acids with (homo)-piperazinyl linkers and rhodamine residues were shown to act as mitocans. IC_50_ values in the single-digit or even sub-nanomolar nanomolar concentration range could be obtained with relatively high selectivity between malignant and non-malignant cells. Depending on the triterpene carboxylic acid used (or its degree of hydroxylation/acetylation), 2,3,23-tris-acetylated triterpene carboxylic acid derivatives interact preferentially with mitochondrial membranes with the almost complete shutdown of mitochondrial ATP synthesis, whereas with mono-acetylated analogues the conjugates could also be detected in the cell nucleus in some cases [4,5,6].

Currently, hybrids of pentacyclic triterpene carboxylic acids with a distal-linked, substituted *N*-methylpyridinium radical, so-called F16 derivatives [7,8], can also be prepared, which exhibit low EC_50_ values with good selectivity [9,10,11]. A decrease in the intensity of ADP phosphorylation was also observed for these derivatives.

In extensions of our previous investigations of triterpenes to diterpenes [12,13,14], we were able to present and investigate cytotoxic derivatives of isosteviol, a stachane-type diterpene, for the first time and to achieve initial success [15]. Therefore, it was obvious to also make corresponding F16 derivatives of steviol and isosteviol synthetically accessible and to investigate their cytotoxic potential.

## 2. Results

**Scheme 1:**
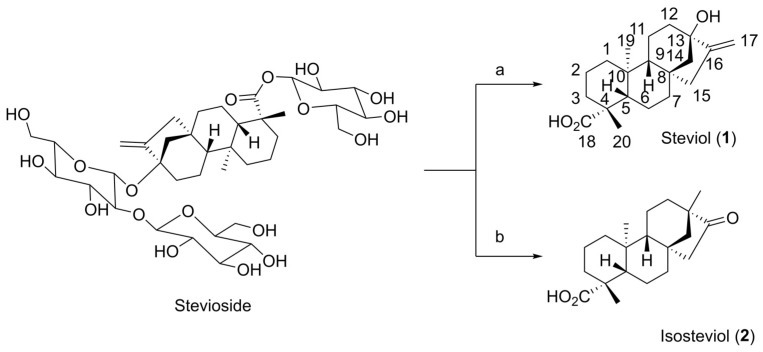
Synthesis of steviol (**1**) and isosteviol (**2**) from stevioside; numbering scheme as well as reactions and conditions: (a) NaIO_4_, H_2_O, 21 °C, 1 d, 26%; (b) aq. HCl, MeOH, reflux, 2 h, 63%.

Steviol (**1**) [16,17,18,19,20,21,22] and isosteviol (**2**) [23,24,25] can be obtained by hydrolysis from commercially available stevioside (Scheme 1). Stevioside is used in large quantities in food manufacturing as a non-cariogenic sweetener with high sweetness. Its acid hydrolysis [26,27,28,29] proceeded smoothly and yielded isosteviol (**2**) in 63% isolated yield (Scheme 1). Meanwhile, the preparation of **1** is more problematic, as the degradation reaction described in the literature (Malaprade cleavage [30,31,32] with NaIO_4_ followed by alkaline hydrolysis) of stevioside gave **1** in only 20–30% yield. Our yield is in excellent agreement with the reported data [29] of M. Ukiya; previously reported [30,31,32] yields ranging between 57 and 70% could not be reproduced in our labs. Alternatively, enzymatic hydrolyses have been described in the literature [33,34], but the enzymes used in these reactions are not commercially available or are difficult to obtain. Our own experiments with commercially available pectinases (from *Aspergillus niger*), pancreatinases (porcine or hog) and β-glucosidases (almonds, *A. niger*) in acetate buffer at pH = 6 either at 21 °C or 50 °C for a period of 1–7 days with or without co-solvent ethanol or toluene were not successful at all, and only small amounts of **1** were isolated. Therefore, we decided to keep the classical synthesis variant—even if the yields were not satisfactory. Attempts to optimize this reaction were not very successful, and the yields could not be improved.

**Scheme 2:**
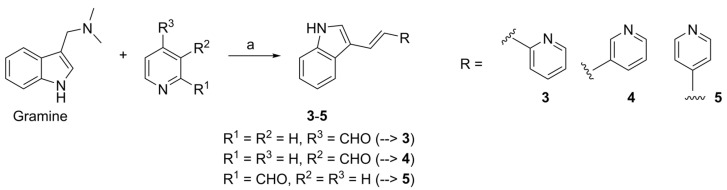
Synthesis of precursors **3**–**5**. Reactions and conditions: MeCN, *n*-Bu_3_P, 85 °C, 1 d, 65% of **3**, 50% of **4** and 46% of **5**, respectively.

**Scheme 3:**
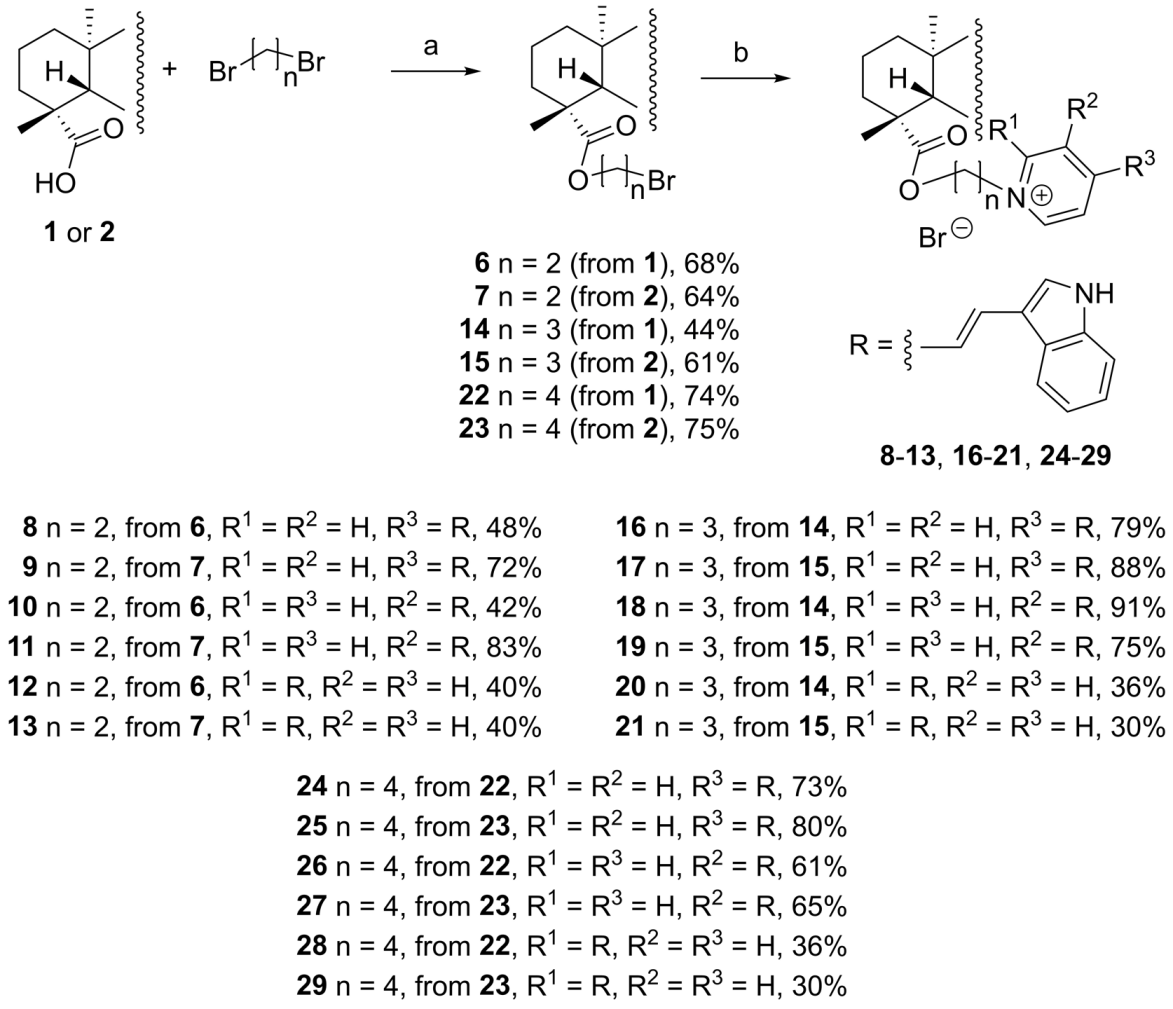
Synthesis of target compounds **8**–**13**, **16**–**21** and **24**–**29**. Reactions and conditions: (a) DMF, MeCN, K_2_CO_3_, 3 h 50 °C; (b) DMF, microwaves (14 h, 120 °C).

The reaction [35] of gramine (Scheme 2) with *o*-, *m*- or *p*-pyridine carbaldehyde furnished (*E*) configurated pyridyl-ethenylindoles **3**–**5**. The coupling constant between the olefinic protons of *J* ~ 16 Hz confirms the (*E*) configuration of the compounds. The esterification of **1** or **2** with 1,2-dibromoethane, 1,3-dibromopropane or 1,4-dibromobutane (Scheme 3) furnished esters **6**/**7**, **14**/**15** and **22**/**23**, respectively, whose microwaves assisted coupling with **3**–**5** furnished final F16 derivatives **8**–**13**, **16**–**21** and **24**–**29**. This small library of compounds hence differs in the kind of diterpenoid skeleton (steviol vs. isosteviol), the length of the spacer between the diterpenoid core and the F16 moiety (2–4 carbons) and the substitution pattern of the F16 unit (*ortho*, *meta* or *para*).

All products were fully characterized by their ^1^H-, ^13^C NMR, IR and UV-vis spectra, as well as MS spectra and microanalysis. The obtained spectroscopic data agreed perfectly with the expected values. The assignment of the signals of the (iso)-steviol skeleton in the respective NMR spectra followed the assignments previously made [15].

**Figure 1:**
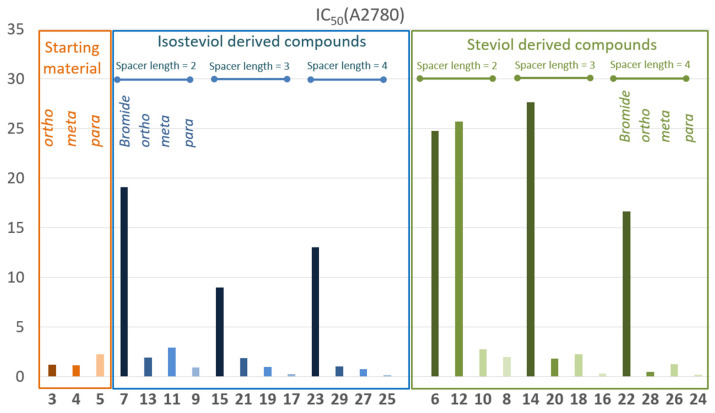
Representative IC_50_ (in μM) values (from SRB assays) for steviol- and isosteviol-derived compounds employing A2780 ovarian carcinoma cells with respect to substitution pattern (*ortho*-, *meta*- or *para* in the pyridinium moiety) and spacer length (2–4).

All compounds were screened for their cytotoxic activity and tumor cell selectivity using our standard model, previously [36] described, comprising a selection of human tumor cell lines representing different solid tumor entities and non-malignant human fibroblasts (CCD18Co) including the cell line pair A2780/A2780cis, a well-known model of acquired drug resistance. The results from these assays are summarized in Table 1 and Table 2 and depicted in Figure 1.

The results reveal that the compounds were most sensitive for A2780 cells, with pyridyl-ethenylindoles **3**–**5** showing IC_50_ values below 3.0 µM. While the spacered diterpenoids were not cytotoxic at all, the F16 hybrids were even more active than their parent compounds, with only marginal differences observed between steviol- and isosteviol-derived variants. Those hybrids holding the linker in the *para*-position proved to be the most active, especially with a higher spacer length. Therefore, compound **25** has an IC_50_ (A2780) of 180 nM, surpassing the activity of comparable rhodamine hybrids. Though the activity is increased, coupling the F16 structures with the diterpenes leads to less selective compounds, except for compounds **17**, **24** and **25**, which retained selectivity. Thus, the three most active compounds were also the most selective. In addition, compounds **17**, **24** and **25** were able to reduce or even overcome drug resistance in the A2780/A2780cis model. Using doxorubicin treatment as a control, the relative resistance of A2780cis compared to A2780 was reproduced, resulting in an approximately 14-fold difference in their IC_50_ values. In conclusion, compounds **17**, **24** and **25**, representing the *para*-linked hybrids with higher spacer length, showed the most promising profiles, combining anti-tumor cell activity, selectivity and the ability to overcome drug resistance. An increased spacer length allows more flexibility, providing effective action at its target site. Moreover, it offers a larger nonpolar region, obviously enhancing the compound’s ability to be accumulated in the mitochondria. However, no complete SAR model can be developed based on these results because the mode of action of the compounds is not yet fully understood. The influence of the orientation of the pyridinium and indole moiety on the cytotoxicity of F16 has already been established by Xu et al. [37]. Our own corresponding molecular modelling calculations for conformational analysis of the derivatives were postponed. In addition, since the intracellular distribution is much more inhomogeneous than, for example, with **AHCS2** (see below), any differences in constitution, configuration and orientation will also have an influence on the mode of action. This will have to be investigated in detail in further experiments as well as with an exact investigation of possible differences between malignant and non-malignant cells.

**Figure 2:**
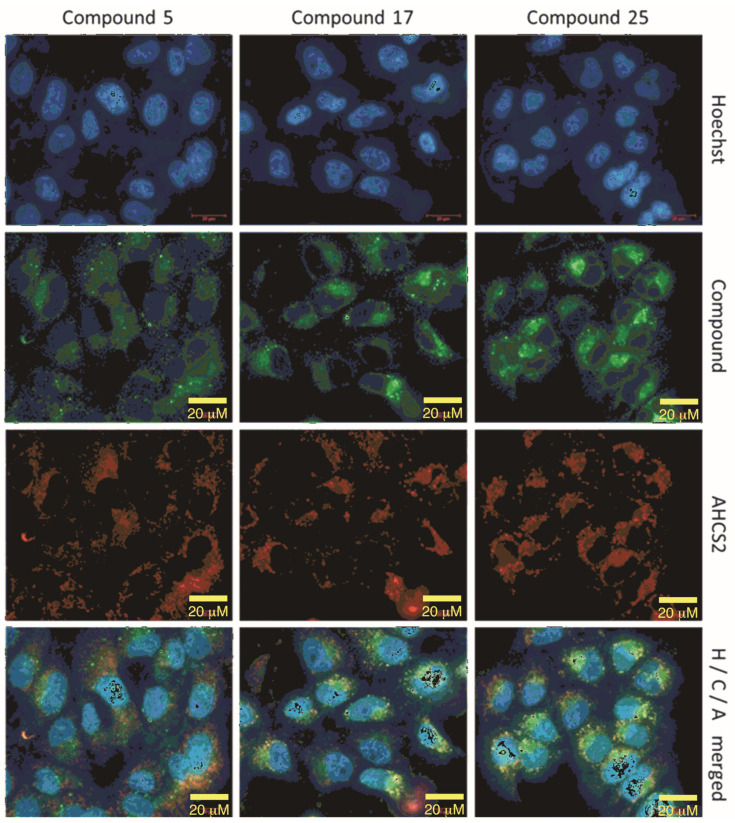
Analysis of subcellular accumulation of **5**, **17** and **25** using combined treatment with the mitochondrial targeting compound **AHCS2** and Hoechst 33,342 for the staining of nuclei showing the preferential mitochondrial localization of compounds with some accumulation in other cellular areas. Scale bar: 20 μm; (for higher resolution, see original images in the Supplementary Materials File).

Next, the subcellular accumulation of compounds **17** and **25** compared to **5** (F16 group) was studied, employing the fluorescent characteristics mediated by the F16 group. Expecting a mitochondrial accumulation of the compounds, our previously described strong mitochondria-targeted and NIR fluorescent agent/mitocan **AHCS2** (compound **21** in ref. [4]) was used, thus enabling simultaneous analysis and direct comparison due to different fluorescence spectra. As shown in Figure 2, a similar subcellular accumulation pattern of the F16 compounds and **AHCS2** could be observed, proving mitochondrial accumulation. However, a thorough analysis, especially of the merged pictures, revealed some minor accumulation of the F16 diterpene hybrids in other cellular areas in addition to mitochondria.

As a possible mode of action—in analogy to F16 or rhodamine conjugates of triterpenes [3,4,8]—an induced cell death combined with a cell cycle arrest, an interruption of the mitochondrial respiratory chain and an influence on the intracellular ATP level seems most likely. Further clarification is reserved for future experiments, as is the performance of clone formation assays or cell scratch assays. The results of our investigations also shed new light on earlier reports on the cytotoxicity of “simple” alkylpyridinium compounds; however, the F16 derivatives investigated in this work are significantly more cytotoxic than, for example, cetylpyridinium chloride—a very commonly used disinfectant [38].

## 3. Conclusions

Steviol and isosteviol were prepared from the commercial sweetener stevioside and converted into lipophilic F16 hybrids. Their cytotoxicity was determined in SRB assays and was shown to depend on both the substitution pattern of the aromatic substituent and the spacer length. Several of the compounds were able to overcome drug resistance in the A2780/A2780cis model. Staining experiments revealed a similar subcellular accumulation pattern of the F16 hybrids as a well-established mitocan, thus demonstrating preferential mitochondrial accumulation, but also minor accumulation in other cellular compartments. The IC_50_ value of compound **25** was as low as 0.18 μM (for A2780 ovarian tumor cells), holding a selectivity for this tumor cell line (as compared to non-malignant human fibroblast CCD18Co) of 59. Despite these very good results, the authors are aware that more intensive biological studies are needed to prove the merits of this new class of compounds.

## 4. Experimental Procedure

### 4.1. General

Reagents were bought from commercial suppliers and used without further purification. The solvents were dried according to the usual procedures. TLC was performed on silica gel (Macherey-Nagel, detection with UV absorption; Macherey-Nagel, Düren, Germany). Melting points have been measured with a Büchi M-565 instrument (Büchi Labortechnik, Flawill, Switzerland). NMR spectra were recorded using VARIAN spectrometers (Varian Germany, Darmstadt, Germany) at 27 °C (δ given in ppm; *J* in Hz, typical experiments for assignments: ^13^C APT, HMBC, HSQC). Numbering in the NMR spectra: 1–20 (diterpene core), 21–24 for O-(CH_2_)_n_-N fragment followed by the pyridinium ring, the ethenyl moiety and the indole moiety. ASAP-MS spectra were taken on an Advion (Advion, Ithaca, NY, USA); expression: CMS-L with an ASAP/APCI Ion source (capillary voltage 150 V, capillary temperature 220 °C and voltage of the ion source: 15 V; APCI source temperature 300 °C with 5 μA). IR spectra were recorded on a Perkin-Elmer Spectrum Two (UATR Two Unit, Perkin-Elmer GmbH, Rodgau, Germany). The human cancer cell lines A2780 (ECACC #93112519), A2780Cis (ECACC # 93112517), A549 (ATCC—CCL-185), HT29 (ATCC—HTB-38) and MCF7 (ATCC—HTB-22) were cultivated in RPMI1640 medium, and non-malignant human fibroblasts CCD18Co (ATCC—CRL-1459) were grown in MEME (both from Sigma-Aldrich, St. Louis, MO, USA). Both media were supplemented with 10% fetal bovine serum (Biowest, Nuaillé, France) and 1% penicillin-streptomycin (Sigma-Aldrich).

Cytotoxic activities of compounds were analyzed using the SRB cytotoxicity assay. Cells were seeded in 96-well plates and after 24 h were treated with serial dilutions of compounds for 72 h. All subsequent steps were performed according to the previously described SRB assay protocol [36]. Dose–response curves and calculation of IC_50_ values, including standard deviations, were carried out using GraphPad Prism8.

Analysis of subcellular localization of compounds was performed in A549 cells using the established mitochondrial targeting compound **AHCS2** [4] for direct comparison. For all procedures, RPMI 1640 media without phenol-red (Pan-Biotech GmbH, Aidenbach, Germany) was used. Cells were seeded in a µ-Plate 96-well black plate (ibiTreat: #1.5 polymer coverslip bottom, ibidi GmbH, Gräfelfing, Germany) at a cell density of 30.000 per well. After 24 h, cells were treated with 10 µM of compound **5** or 1 µM of compound **17** or 1 µM of compound **25**, each together with 20 nM of compound **AHCS2**. After 24 h, Hoechst 33342 (Sigma-Aldrich) was added and live cell imaging was performed on an Axio Observer 7 (Carl Zeiss Microscopy Deutschland GmbH, Oberkochen, Germany) using the settings for Ex/Em, as follows: Hoechst 33342 (385 nm/425 nm), F16 compounds (475 nm/514 nm), **AHCS2** (735 nm/785 nm). For simultaneous analysis, multiple Z-stacked images were taken and resulting pictures were reconstructed using the ZEN 3.5 pro software (Zeiss).

### 4.2. Syntheses

#### 4.2.1. (4α) 13-Hydroxy-kaur-16-en-18-oic Acid (**1**)

To a solution of stevioside (100.0 g, 0.12 mol) in dist. water (8.0 L), NaIO_4_ (160.0 g, 0.75 mol) was added and the mixture was stirred for 1 day at 21 °C. Finely ground KOH (650.0 g, 11.6 mol) was added, and the mixture was stirred under reflux for 3 h. The mixture was cooled to 0 °C and HOAc (650 mL) was slowly added; the mixture was extracted with ether (1.8 L). The combined organic layers were washed with water, dried (MgSO_4_), and the solvents were removed under reduced pressure. The residue was subjected to re-crystallization from MeOH to yield **1** (10.5 g, 26%) as a colorless solid: *R*_f_ = 0.47 (SiO_2_, CHCl_3_/MeOH, 9:1); m.p. = 204–206 °C [lit.: [39] 212–213 °C]; ${\left[\alpha \right]}_{D}^{20}$ = −62.88° (*c* = 0.09, CHCl_3_) [lit.: [40] ${\left[\alpha \right]}_{D}^{20}$ = −55.2° (CHCl_3_)]; IR (ATR): ν = 2945*br*, 1694*w*, 1456*w*, 1184*w*, 757*w* cm^−1^; ^1^H NMR (500 MHz, CDCl_3_): δ = 4.98 (*s*, 1H, 17-H), 4.81 (*s*, 1H, 17-H), 2.20 (*dt*, *J* = 17.0, 2.9 Hz, 1H, 15-H), 2.14 (*td*, *J* = 13.4, 3.3 Hz, 1H, 3-H), 2.12–2.04 (*m*, 2H, 14-H, 15-H), 1.94 (*td*, *J* = 13.8, 4.1 Hz, 1H, 2-H), 1.92–1.70 (*m*, 5H, 1-H, 6-H, 11-H, 12-H), 1.65–1.51 (*m*, 3H, 7-H, 11-H, 12-H), 1.48–1.39 (*m*, 2H, 2-H, 7-H), 1.30 (*dd*, *J* = 10.5, 2.7 Hz, 1H, 14-H), 1.23 (*s*, 3H, 19-H), 1.08 (*dd*, *J* = 12.1, 2.3 Hz, 1H, 5-H), 1.04–0.99 (*m*, 1H, 3-H), 0.99–0.96 (*m*, 1H, 9-H), 0.95 (*s*, 3H, 20-H), 0.84–0.79 (*m*, 1H, 1-H) ppm; ^13^C NMR (126 MHz, CDCl_3_): δ = 183.77 (C-18), 155.80 (C-16), 103.20 (C-17), 80.51 (C-13), 57.03 (C-5), 53.96 (C-9), 47.54 (C-15), 47.05 (C-14), 43.74 (C-4), 41.87 (C-8), 41.36 (C-7), 40.64 (C-1), 39.65 (C-10), 39.50 (C-12), 37.84 (C-3), 28.92 (C-19), 21.93 (C-6), 20.59 (C-11), 19.14 (C-2), 15.55 (C-20) ppm; MS (ESI, MeOH): *m/z* (%) 317 (100%, [M − H]^−^).

#### 4.2.2. (4α, 8β, 13β) 13-Methyl-16-oxo-17-norkauran-18-oic Acid (**2**)

A solution of stevioside (86.0 g, 0.10 mol) in MeOH (500 mL) and aq. HCl (33%, 90 mL) was heated under reflux for 2 h. Stirring at 21 °C was continued overnight, water (1200 mL) was added slowly, and the precipitate was filtered off, dried and re-crystallized from EtOH (300 mL) to yield **2** (21.6 g, 63%) as a colorless solid: *R*_f_ = 0.71 (SiO_2_, CHCl_3_/MeOH 9:1); m.p. = 229–231 °C [lit.: [41] 228–230 °C]; ${\left[\alpha \right]}_{D}^{20}$ = −84.02° (*c* = 0.15, CHCl_3_) [lit.: [39] ${\left[\alpha \right]}_{D}^{20}$ = −79.3° (EtOH)]; IR (ATR), ^1^H NMR and ^13^C NMR as previously reported; MS (ESI, MeOH/CHCl_3_, 4:1): *m*/*z* (%) 317 (100%, [M − H]^−^).

#### 4.2.3. General Procedure for the Synthesis of Pyridyl-ethenylindoles **3**–**5** (GPA)

A mixture of 2-, 3- or 4-pyridinecarboxaldehyde (1.5 equiv), tri-*n*-butyl-phosphine (1.5 equiv) and gramine (3-dimethylaminomethyl-indole, 3 equiv) in dry acetonitrile (75 mL) was stirred at 85 °C for 1 day; [35] the volatiles were removed under reduced pressure, and the residue was purified by chromatography.

#### 4.2.4. 3-[(*E*)-2-Pyridin-2-yl-ethenyl]-1H-indole (**3**)

Following GPA from gramine (5.25 g, 30 mmol), acetonitrile (75 mL), *n*-PBu_3_ (12 mL, 46 mmol) and 24-pyridinecarbaldehyde (4.2 mL, 45 mmol) followed by chromatography (SiO_2_; hexanes/ethyl acetate, 1:1), **5** (3.05 g, 46%) was obtained as a yellowish solid: *R*_f_ = 0.52 (SiO_2_, hexanes/ethyl acetate, 1:1); m.p. = 185–187 °C (lit: [41] 190–191 °C); IR (ATR): ν = 3128*w*, 3086*w*, 3041*w*, 2972*w*, 2919*w*, 2879*w*, 1632*m*, 1565*m*, 1523*w*, 1500*m*, 1451*m*, 1417*m*, 1346*br*, 1283*m*, 1252*m*, 1222*w*, 1185*w*, 1151*w*, 1138*w*, 1117*m*, 1084*w*, 1042*w*, 1025*w*, 977*w*, 952*s*, 913*w*, 881*w*, 853*w*, 817*w*, 771*br*, 739*s*, 701*s*, 627*m*, 618*m*, 564*w*, 547*w*, 426*m* cm^−1^; UV-Vis (MeOH): λ_max_ (log ε) = 303.67 nm (2.38); ^1^H NMR (500 MHz, DMSO-d_6_): δ = 11.42 (*s*, 1H, NH), 8.50 (*ddd*, *J* = 4.8, 1.8, 0.7 Hz, 1H, 15-H), 7.99 (*d*, *J* = 7.8 Hz, 1H, 3-H), 7.88 (*d*, *J* = 16.1 Hz, 1H, 9-H), 7.73 (*d*, *J* = 2.7 Hz, 1H, 8-H), 7.69 (*td*, *J* = 7.6, 1.8 Hz, 1H, 13-H), 7.47 (*d*, *J* = 7.9 Hz, 1H, 12-H), 7.45–7.41 (*m*, 1H, 2-H), 7.18 (*dd*, *J* = 7.0, 1.2 Hz,1H, 5-H), 7.15(*d*, *J* = 16.3 Hz, 1H, 10-H), 7.20–7.09 (m, 1H, 4-H), 7.12–7.10 (m, 1H, 14-H) ppm; ^13^C NMR (126 MHz, DMSO-d_6_): δ = 156.42 (C-15), 149.20 (C-11), 137.19 (C-1), 136.51 (C-13), 127.85 (C-9), 127.45 (C-6), 126.38 (C-8), 125.12 (C-3), 124.15 (C-10), 122.63 (C-12), 121.93 (C-4), 121.22 (C-14), 119.99 (C-5), 112.03 (C-7), 111.68 (C-2) ppm; MS (ESI, DMSO/CHCl_3_, 4:1): *m*/*z* (%) 221 (100%, [M + H]^+^); analysis calcd. for C_15_H_12_N_2_ (220.28): C 81.79, H 5.49, N 12.72; found: C 81.63, H 5.70, N 12.45.

#### 4.2.5. 3-[(*E*)-2-Pyridin-3-yl-ethenyl]-1H-indole (**4**)

Following GPA from gramine (5.25 g, 30 mmol), acetonitrile (75 mL), *n*-PBu_3_ (12 mL, 46 mmol) and 3-pyridinecarbaldehyde (4.2 mL, 45 mmol), followed by chromatography (SiO_2_; hexanes/ethyl acetate, 1:1), **4** (3.32 g, 50%) [42,43,44,45] was obtained as a yellowish solid: *R*_f_ = 0.25 (SiO_2_, hexanes/ethyl acetate, 1:1); m.p. = 194–195 °C (lit.: [35] 194–195 °C); IR (ATR): ν = 3129*w*, 3089*w*, 3039*w*, 2970*w*, 2921*w*, 2881*w*, 1630*m*, 1568*m*, 1524*w*, 1499*m*, 1452*m*, 1416*m*, 1348*br*, 1281*m*, 1250*m*, 1224*w*, 1186*w*, 1153*w*, 1136*w*, 1118*m*, 1083*w*, 1042*w*, 1026*w*, 976*w*, 953*s*, 911*w*, 882*w*, 851*w*, 816*w*, 772*br*, 740*s*, 699*s*, 629*m*, 617*m*, 563*w*, 548*w*, 424*s* cm^−1^; UV-Vis (MeOH): λ_max_ (log ε) = 335.01 nm (2.51); ^1^H NMR (500 MHz, DMSO-d_6_): δ = 11.40 (*s*, 1H, NH), 8.77 (*d*, *J* = 2.2 Hz, 1H, 15-H), 8.37 (*dd*, *J* = 4.6, 1.6 Hz, 1H, 14-H), 8.05 (*d*, *J* = 7.8 Hz, 1H, 2-H), 7.99 (*dt*, *J* = 7.9, 1.8 Hz, 1H, 12-H), 7.68 (*dt*, *J* = 2.6 Hz, 1H, 8-H), 7.56 (*d*, *J* = 16.6 Hz, 1H, 9-H), 7.45 (*d*, *J* = 7.8 Hz, 1H, 3-H), 7.34 (*dd*, *J* = 7.9, 4.7 Hz, 1H, 13-H), 7.21–7.16 (m1H, 5-H), 7.14 (*td*, *J* = 7.4, 6.8, 1.2 Hz, 1H, 4-H), 7.12 (*d*, *J* = 16.6 Hz, 1H, 10-H) ppm; ^13^C NMR (126 MHz, DMSO-d_6_): δ = 147.95 (C-15), 147.47 (C-14), 137.54 (C-6), 134.76 (C-11), 131.91 (C-12), 127.17 (C-8), 125.57 (C-1), 125.14 (C-9), 124.10 (C-13), 122.37 (C-5), 120.38 (C-2), 120.31 (C-4), 119.93 (C-10), 114.02 (C-7), 112.45 (C-3) ppm; MS (ESI, MeOH/DMSO, 4:1): *m*/*z* (%) 219 (77%, [M − H]^−^); analysis calcd. for C_15_H_12_N_2_ (220.28): C 81.79, H 5.49, N 12.72; found: C 81.58, H 5.79, N 12.40.

#### 4.2.6. 3-[(*E*)-2-Pyridin-4-yl-ethenyl]-1H-indole (**5**)

Following GPA from gramine (5.25 g, 30 mmol), acetonitrile (75 mL), *n*-PBu_3_ (12 mL, 46 mmol) and 4-pyridinecarbaldehyde (4.2 mL, 45 mmol), followed by chromatography (SiO_2_; hexanes/ethyl acetate, 1:1), **3** (4.31 g, 65%) was obtained as a reddish solid [37,46,47,48,49,50,51]: *R*_f_ = 0.26 (SiO_2_, hexanes/ethyl acetate, 1:1); m.p. = 254–255 °C (lit.: [37] 255–256 °C); C; IR (ATR): ν = 3127*w*, 3086*w*, 3064*w*, 3026*w*, 2958*w*, 2915*w*, 2869*w*, 1625*m*, 1592*s*, 1547*m*, 1519*m*, 1493*m*, 1444*s*, 1420*s*, 1352*w*, 1332*w*, 1303*w*, 1279*m*, 1247*s*, 1215*m*, 1200*m*, 1153*w*, 1135*w*, 1121*m*, 1093*m*, 1065*w*, 1018*w*, 999*m*, 962*s*, 902*w*, 869*m*, 830*m*, 802*m*, 772*w*, 732*s*, 666*w*, 618*m*, 559*w*, 546*w*, 526*m*, 496*w*, 422*m* cm^−1^; UV-Vis (MeOH): λ_max_ (log ε) = 352.44 nm (1.26); ^1^H NMR (400 MHz, DMSO-d_6_): δ = 11.63–11.45 (*s*, 1H, NH), 8.47 (*d*, *J* = 6.1 Hz, 2H, 13-H, 14-H), 8.03 (*dd*, *J* = 7.5, 1.5 Hz, 1H, 5-H), 7.73 (*d*, *J* = 13.8 Hz, 1H, 9-H,), 7.75 (*s*, 1H, 8-H), 7.52 (*d*, J = 6.2 Hz, 2H, 12-H, 15-H), 7.43 (*d*, *J* = 7.9 Hz, 1H, 2-H), 7.20–7.11 (*m*, 2H, 3-H, 4-H), 7.04 (*d*, *J* = 16.5 Hz, 1H, 10-H) ppm; ^13^C NMR (101 MHz, DMSO-d_6_): δ = 150.18 (C-13, C-14), 146.38 (C-11), 137.61 (C-1), 128.44 (C-8), 127.83 (C-9), 125.50 (C-6), 122.54 (C-4), 120.67 (C-10), 120.55 (C-3), 120.43 (C-5), 120.37 (C-12, C-15), 113.65 (C-7), 112.54 (C-2) ppm; MS (ESI, MeOH/DMSO, 4:1): *m*/*z* (%) 219 (83%, [M − H]^−^); analysis calcd. for C_15_H_12_N_2_ (220.28): C 81.79, H 5.49, N 12.72; found: C 81.50, H 5.71, N 12.46.

#### 4.2.7. General Procedure for the Synthesis of Alkyl Bromides **6**, **7**, **14**, **15**, **22** and **23** (GPB)

To a solution of **1** or **2** in dry DMF/ACN (3:1), finely ground K_2_CO_3_ (2 equiv.) and 1,2-dibromoethane, 1,3-dibromopropane or 1,4-dibromobutane (4 equiv.) were added, and the mixture was stirred for 3 h at 50 °C [8]. The volatiles were removed under reduced pressure, and the residue was subjected to chromatography to yield compounds **6** and **7** (from dibromoethane), **14** and **15** (from dibromopropane) and **22** and **23** (from dibromobutane), respectively.

#### 4.2.8. General Procedure for the Synthesis of the F16 Conjugates **8**–**13**, **16**–**21** and **24**–**29** (GPC)

A mixture of bromide **6**, **7**, **14**, **15**, **22** or **23** (1.0 mmol) and pyridyl-ethenylindoles **3**–**5** (1 equiv.) in dry DMF (10 mL) was stirred in a microwave for 14 h at 120 °C (microwave assisted; Anton Parr Monowave apparatus; Anton Paar GmbH, Graz, Austria). The volatiles were removed under reduced pressure and the residue was subjected to chromatography to yield products **8**–**13**, **16**–**21** and **24**–**29**.

#### 4.2.9. 2-Bromoethyl (4α)-13-Hydroxykaur-16-en-18-oate (**6**)

Following GPB from **1** (0.5 g, 1.57 mmol), K_2_CO_3_ (0.434 g, 3.1 mmol), 1,2-dibromoethane (0.55 mL, 6.3 mmol) followed by chromatography (SiO_2_, hexanes/ethyl acetate, 6:1), **6** (0.45 g, 68%) was obtained as a colorless solid: *R*_f_ = 0.33 (SiO_2_, hexanes/ethyl acetate, 8:2); m.p. = 115–117 °C; ${\left[\alpha \right]}_{D}^{20}$ = −57.06° (*c* = 0.145, CHCl_3_); IR (ATR): ν = 3554*m*, 2934*m*, 2853*m*, 1709*s*, 1460*m*, 1443*m*, 1388*w*, 1366*w*, 1316*w*, 1282*w*, 1208*m*, 1138*s*, 1113*m*, 1092*m*, 1057*w*, 1008*w*, 967*m*, 877*w*, 819*w*, 774*w*, 678*w*, 578*w*, 531*w* cm^−1^; ^1^H NMR (500 MHz, CDCl_3_): δ = 4.97 (*s*, 1H, 17-H), 4.81 (*s*, 1H, 17-H), 4.42–4.30 (*m*, 2H, 21-H), 3.53 (*t*, *J* = 5.8 Hz, 2H, 22-H), 2.17 (*dd*, *J* = 9.0, 5.6 Hz, 2H, 12-H_a_, 15-H_a_), 1.93–1.71 (*m*, 4H, 2-H_a_, 6-H, 1-H_a_), 1.71–1.57 (*m*, 5H, 3-H, 7-H, 11-H_a_), 1.57–1.35 (*m*, 5H, 2-H_b_, 11-H, 14-H), 1.20 (*s*, 3H, 19-H), 1.10–0.94 (*m*, 2H, 12-H_b_, 5-H), 0.88 (*s*, 3H, 20-H), 0.87–0.76 (*m*, 2H, 1-H, 9-H) ppm; ^13^C NMR (126 MHz, CDCl_3_): δ = 177.27 (C-18), 143.61 (C-16), 103.04 (C-17), 82.73 (C-13), 63.99, 56.85 (C-5), 53.88 (C-9), 51.00 (C-15), 48.08 (C-14), 44.13 (C-4), 41.45 (C-8), 40.98 (C-1), 39.75 (C-7), 39.50 (C-10), 38.16 (C-12), 31.89 (C-3), 29.05 (C-22), 28.96 (C-19), 21.15 (C-6), 20.93 (C-11), 19.18 (C-2), 15.47 (C-20) ppm; MS (ESI, MeOH/CHCl_3_, 4:1): *m*/*z* (%) 345 (100%, [M − Br]^+^); analysis calcd. for C_22_H_33_O_3_Br (425.41): C 62.12, H 7.82; found: C 61.97, H 8.03.

#### 4.2.10. 2-Bromoethyl (4α, 8β, 13β) 13-Methyl-16-oxo-17-norkauran-18-oate (**7**)

Following GPB from **2** (0.5 g, 1.57 mmol), K_2_CO_3_ (0.434 g, 3.1 mmol), 1,2-dibromoethane (0.55 mL, 6.3 mmol) followed by chromatography (SiO_2_, hexanes/ethyl acetate, 9:1), **7** (0.43 g, 64%) was obtained as a colorless solid: *R*_f_ = 0.7 (SiO_2_; hexanes/ethyl acetate, 4:1); m.p. = 141–144 °C; ${\left[\alpha \right]}_{D}^{20}$ = −63.27° (*c* = 0.156, CHCl_3_); IR (ATR): ν = 2935*m*, 2883*w*, 2839*m*, 1723*s*, 1470*m*, 1451*m*, 1431*w*, 1386*w*, 1322*w*, 1291*w*, 1229*m*, 1209*m*, 1176*s*, 1146*s*, 1132*s*, 1093*m*, 1060*w*, 1030*w*, 1017*w*, 996*w*, 974*m*, 952*w*, 928*w*, 880*w*, 856*w*, 803*w*, 759*m*, 696*w*, 587*w*, 574*w*, 561*w*, 535*w*, 505*w*, 462*w* cm^−1^; ^1^H NMR (500 MHz, CDCl_3_): δ = 4.42–4.32 (*m*, 2H, 21-H), 3.53 (*t*, *J* = 5.7 Hz, 2H, 22-H), 2.64 (*dd*, *J* = 18.6, 3.8 Hz, 1H, 15-H), 2.21 (*d*, *J* = 13.5 Hz, 1H, 3-H), 1.91 (*dd*, *J* = 14.1, 2.6 Hz, 2H, 2-H, 15-H), 1.86–1.76 (*m*, 3H, 1-H, 6-H, 11-H), 1.75–1.52 (*m*, 6H, 6-H, 7-H, 11-H, 12-H, 14-H), 1.52–1.34 (*m*, 3H, 2-H, 12-H, 14-H), 1.23 (*s*, 3H, 19-H), 1.22–1.13 (*m*, 2H, 5-H, 9-H), 1.04 (*td*, *J* = 13.5, 4.2 Hz, 1H, 3-H), 0.98 (*s*, 3H, 17-H), 0.92 (*td*, *J* = 13.3, 4.2 Hz, 1H, 1-H), 0.75 (*s*, 3H, 20-H) ppm; ^13^C NMR (126 MHz, CDCl_3_): δ = 222.39 (C-16), 177.03 (C-18), 63.94 (C-21), 57.09 (C-5), 54.69 (C-9), 54.28 (C-14), 48.70 (C-13), 48.42 (C-15), 43.97 (C-4), 41.51 (C-7), 39.79 (C-1), 39.48 (C-8), 38.09 (C-10), 37.93 (C-3), 37.31 (C-12), 28.95 (C-22), 28.93 (C-19), 21.73 (C-6), 20.35 (C-11), 19.85 (C-17), 18.89 (C-2), 13.45 (C-20) ppm; MS (ESI, MeOH:CHCl_3_ 4:1): *m*/*z* (%) 345 (100%, [M − Br]^+^); analysis calcd. for C_22_H_33_O_3_Br (425.41): C 62.12, H 7.82; found: C 61.87, H 7.97.

#### 4.2.11. 2-{4-[(E)-2-(1H-Indol-3-yl)ethenyl]-pyridinium-1-yl}-ethyl (4α)-13-Hydroxykaur-16-en-18-oate Bromide (**8**)

Following GPC from **6** (0.23 g, 0.54 mmol) and **3** (0.12 g, 0.54 mmol) followed by chromatography (SiO_2_, CHCl_3_/MeOH, 9:1), **8** (0.15 g, 48%) was obtained as a reddish solid: *R*_f_ = 0.2 (SiO_2_, CHCl_3_/MeOH, 9:1); m.p. = 188–190 °C; ${\left[\alpha \right]}_{D}^{20}$ = −27.39° (*c* = 0.076, MeOH); IR (ATR): ν = 2924*w*, 2850*w*, 1721*w*, 1594*m*, 1574*m*, 1500*m*, 1430*m*, 1363*w*, 1317*br*, 1275*w*, 1245*m*, 1203*w*, 1178*m*, 1131*m*, 1043*w*, 958*w*, 870*w*, 744*m*, 611*w*, 565*br*, 509*w*, 424*w* cm^−1^; UV-Vis (MeOH): λ_max_ (log ε) = 436.98 nm (4.46); ^1^H NMR (500 MHz, DMSO-d_6_): δ = 11.99 (*s*, 1H, NH), 8.83 (*d*, *J* = 6.9 Hz, 2H, 23-H, 27-H), 8.32 (*s*, 1H, 37-H), 8.29 (*d*, *J* = 16.2 Hz, 1H, 29-H), 8.19–8.13 (*m*, 2H, 24-H, 26-H), 7.98 (*d*, *J* = 2.9 Hz, 1H, 35-H), 7.51 (*d*, *J* = 7.5 Hz, 1H, 32-H), 7.30 (*d*, *J* = 16.2 Hz, 1H, 28-H), 7.27–7.19 (*m*, 2H, 33-H, 34-H), 4.83–4.71 (*m*, 2H, 22-H), 4.75–4.64 (*m*, 2H, 17-H), 4.58 (*m*, 2H, 21-H), 2.54–2.46 (*m*, 1H, 15-H), 1.95 (*s*, 1H, 12-H_a_), 1.84–1.65 (*m*, 4H, 3-H, 6-H_a_, 11-H_a_, 15-H_a_), 1.65–1.43 (*m*, 4H, 1-H_a_, 2-H_a_, 11-H_b_, 14-H_a_), 1.43–1.17 (*m*, 3H, 2-H_b_, 6-H_b_, 7-H_a_), 1.14 (*d*, *J* = 10.0 Hz, 2H, 12-H_b_, 14-H_b_), 1.03 (*s*, 3H, 19-H), 0.96 (*q*, *J* = 13.6, 12.6 Hz, 2H, 3-H, 7-H_b_), 0.84 (*d*, *J* = 8.0 Hz, 2H, 5-H, 9-H), 0.72 (*t*, 1H, 1-H_b_), 0.61 (*s*, 3H, 20-H) ppm; ^13^C NMR (126 MHz, DMSO-d_6_): δ = 176.64 (C-18), 156.59 (C-16), 155.67 (C-25), 144.13 (C-23, C-27), 138.00 (C-36), 137.66 (C-29), 133.12 (C-35), 125.39 (C-31), 123.42 (C-31), 122.23 (C-33), 121.67 (C-24, C-26), 120.94 (C-34), 117.23 (C-28), 114.16 (C-30), 113.09 (C-32), 102.96 (C-17), 79.03 (C-13), 62.73 (C-21), 57.94 (C-22), 56.21 (C-5), 53.50 (C-9), 47.63 (C-15), 46.50 (C-14), 43.74 (C-4), 43.70 (C-8), 41.43 (C-7), 41.19 (C-1), 39.39 (C-10), 39.12 (C-12), 37.72 (C-3), 28.63 (C-19), 21.93 (C-6), 20.32 (C-11), 18.99 (C-2), 15.42 (C-20) ppm; MS (ESI, MeOH/DMSO, 4:1): *m*/*z* (%) 566 (66%, [M − Br]^+^); analysis calcd. for C_37_H_45_N_2_O_3_Br (645.68): C 68.83, H 7.03, N 4.34; found: C 68.59, H 7.26, N 4.11.

#### 4.2.12. 2-{4-[(E)-2-(1H-Indol-3-yl)-ethenyl]pyridinium-1-yl}-ethyl (4α, 8β, 13β) 13-Methyl-16-oxo-17-norkauran-18-oate Bromide (**9**)

Following GPC from **7** (0.215 g, 0.51 mmol) and **3** (0.115 g, 0.51 mmol) followed by chromatography (SiO_2_, CHCl_3_/MeOH, 9:1), **9** (0.2 g, 72%) was obtained as a reddish solid: *R*_f_ = 0.32 (SiO_2_, CHCl_3_/MeOH, 9:1); m.p. = 194–196 °C; ${\left[\alpha \right]}_{D}^{20}$ = −43.56° (*c* = 0.101, MeOH); IR (ATR): ν = 2925*br*, 2848*w*, 1726*s*, 1645*w*, 1594*s*, 1574*s*, 1500*s*, 1431*s*, 1356*w*, 1317*w*, 1275*w*, 1246*m*, 1204*m*, 1176*s*, 1130*s*, 1043*w*, 960*w*, 870*w*, 745*s*, 663*w*, 612*w*, 563*w*, 509*w*, 425*w* cm^−1^; UV-Vis (MeOH): λ_max_ (log ε) = 438.15 nm (4.79); ^1^H NMR (500 MHz, DMSO-d_6_): δ = 11.98 (*s*, 1H, NH), 8.83 (*d*, *J* = 6.8 Hz, 2H, 23-H, 27-H), 8.31 (*s*, 1H, 37-H), 8.29 (*d*, *J* = 16.1 Hz, 1H, 29-H), 8.19 (*d*, *J* = 7.0 Hz, 2H, 24-H, 26-H), 8.12 (*d*, *J* = 7.6 Hz, 1H, 32-H), 7.51 (*d*, *J* = 7.6 Hz, 1H, 35-H), 7.29 (*d*, *J* = 16.1 Hz, 1H, 28-H), 7.27–7.19 (*m*, 2H, 33-H, 34-H), 4.77 (*s*, 2H, 22-H), 4.57–4.50 (*m*, 1H, 21-H_a_), 4.44–4.37 (*m*, 1H, 21-H_b_), 2.29–2.20 (*m*, 1H, 15-H_a_), 1.95 (*d*, *J* = 12.8 Hz, 1H, 3-H_a_), 1.75 (*d*, *J* = 18.5 Hz, 1H, 15-H_b_), 1.65–1.46 (*m*, 5H, 1-H_a_, 6-H_a_, 2-H_a_, 7-H_a_, 11-H_a_), 1.40–1.20 (*m*, 6H*,* 2-H*_b_,* 7-H_b_, 12-H, 14-H), 1.13–1.02 (*m*, 3H, 5-H, 6-H_b_, 9-H), 1.06 (*s*, 3H, 19-H), 1.02–0.91 (*m*, 2H*,* 3-H*_b_,* 11-H_b_), 0.88–0.78 (*m*, 1H, 1-H_b_), 0.75 (*s*, 3H, 17-H), 0.44 (*s*, 3H, 20-H) ppm; ^13^C NMR (126 MHz, DMSO-d_6_): δ = 220.8 (C-16), 176.6 (C-18), 155.7 (C-25), 144.2 (C-23, C-27), 138.0 (C-36), 137.8 (C-29), 133.2 (C-37), 125.4 (C-31), 123.4 (C-24, C-26), 122.2 (C-28), 121.7 (C-34), 120.9 (C-33), 117.2 (C-32), 114.2 (C-30), 113.1 (C-35), 63.0, 57.9, 56.1 (C-5), 54.0 (C-9), 53.6 (C-14), 48.2 (C-13), 48.1 (C-15), 43.7 (C-4), 40.8 (C-7), 39.4 (C-1), 39.3 (C-8), 37.8 (C-10), 37.7 (C-3), 36.9 (C-12), 28.7 (C-19), 21.8 (C-6), 20.2 (C-11), 20.1 (C-17), 18.8 (C-2), 13.2 (C-20) ppm; MS (ESI, MeOH/DMSO, 4:1): *m*/*z* (%) 566 (85%, [M − Br]^+^); C_37_H_45_N_2_O_3_Br (645.68): C 68.83, H 7.03, N 4.34; found: C 68.71, H 7.19, N 4.05.

#### 4.2.13. 2-{3-[(E)-2-(1H-Indol-3-yl)ethenyl]-pyridinium-1-yl}-ethyl (4α)-13-Hydroxykaur-16-en-18-oate Bromide (**10**)

Following GPC from **6** (0.23 g, 0.54 mmol) and **4** (0.12 g, 0.54 mmol) followed by chromatography (SiO_2_, CHCl_3_/MeOH, 9:1), **10** (0.13 g, 42%) was obtained as a yellowish solid: *R*_f_ = 0.12 (SiO_2_, CHCl_3_/MeOH, 9:1); m.p. = 184–187 °C; ${\left[\alpha \right]}_{D}^{20}$ = −6.74° (*c* = 0.095, MeOH); IR (ATR): ν = 3370*br*, 2928*m*, 2851*m*, 1725*m*, 1662*w*, 1632*m*, 1615*m*, 1576*m*, 1526*w*, 1503*w*, 1459*m*, 1434*m*, 1387*w*, 1364*w*, 1330*w*, 1276*w*, 1229*m*, 1167*w*, 1148*m*, 1118*m*, 1083*m*, 1048*w*, 967*w*, 920*w*, 874*w*, 819*w*, 747*s*, 681*m*, 611*w*, 571*w*, 528*w*, 500*w*, 475*w*, 425*w* cm^−1^; UV-Vis (MeOH): λ_max_ (log ε) = 360.00 nm (4.17); ^1^H NMR (400 MHz, DMSO-d_6_): δ = 11.6 (*s*, 1H, NH), 9.4 (*s*, 1H, 23-H), 8.9 (*d*, *J* = 5.9 Hz, 1H, 27-H), 8.7 (*d*, *J* = 8.3 Hz, 1H, 25-H), 8.3 (*s*, 1H), 8.1 (*dd*, *J* = 8.1, 6.0 Hz, 1H, 26-H), 8.0 (*d*, *J* = 7.6 Hz, 1H, 32-H), 7.9 (*s*, 1H, 37-H), 7.9 (*d*, *J* = 16.5 Hz, 1H, 29-H), 7.5 (*d*, *J* = 7.4 Hz, 1H, 35-H), 7.2 (*d*, *J* = 16.4 Hz, 1H, 28-H), 7.3–7.1 (*m*, 2H, 33-H, 34-H), 5.0–4.8 (*m*, 3H, 17-H_a_, 22-H), 4.7–4.4 (*m*, 3H, 17-H_b_, 21-H), 2.0–1.9 (*m*, 3H, 15-H, 3-H_a_), 1.7–1.5 (*m*, 6H, 1-H_b_, 2-H, 6-H_b_, 11-H_a_, 14-H_b_), 1.5–1.4 (*m*, 1H, 6-H_a_), 1.3–1.1 (*m*, 6H, 7-H, 11-H_b_, 12-H, 14-H_a_), 1.0 (*s*, 3H, 19-H), 1.0–0.9 (*m*, 2H, 3-H_b_, 5-H), 0.9–0.8 (*m*, 1H, 9-H), 0.7 (*s*, 1H, 1-H_a_), 0.5 (*s*, 3H, 20-H) ppm; ^13^C NMR (101 MHz, DMSO-d_6_): δ = 176.7 (C-18), 156.7 (C-16), 142.4 (C-23), 141.1 (C-27), 140.6 (C-25), 140.0 (C-36), 137.7 (C-24), 133.5 (C-37), 130.8 (C-29), 128.2 (C-26), 125.3 (C-31), 122.9 (C-34), 120.9 (C-33), 120.4 (C-32), 115.7 (C-28), 113.4 (C-30), 112.8 (C-35), 103.0 (C-17), 81.1 (C-13), 63.0 (C-21), 59.8 (C-22), 55.9 (C-5), 53.4 (C-9), 47.5 (C-15), 47.0 (C-14), 43.7 (C-4), 43.7 (C-8), 41.3 (C-7), 40.6 (C-1), 39.4 (C-10), 39.2 (C-12), 37.8 (C-3), 28.5 (C-19), 20.9 (C-6), 18.9 (C-11), 16.8 (C-2), 15.1 (C-20) ppm; MS (ESI, MeOH/DMSO, 4:1): *m*/*z* (%) 566 (70%, [M − Br]^+^); analysis calcd. for C_37_H_45_N_2_O_3_Br (645.67): C 68.83, H 7.02, N 4.34; found: C 68.61, H 7.22, N 4.17.

#### 4.2.14. 2-{3-[(E)-2-(1H-Indol-3-yl)ethenyl]-pyridinium-1-yl}-ethyl (4α)-13-Hydroxykaur-16-en-18-oate bromide Bromide (**11**)

Following GPC from **7** (0.215 g, 0.51 mmol) and **4** (0.115 g, 0.51 mmol) followed by chromatography (SiO_2_, CHCl_3_/MeOH, 9:1), **11** (0.24 g, 83%) was obtained as a yellowish solid: *R*_f_ = 0.22 (SiO_2_, CHCl_3_/MeOH, 9:1); m.p. = 193–194 °C; ${\left[\alpha \right]}_{D}^{20}$ = +0.25° (*c* = 0.081, MeOH); IR (ATR): ν = 2926*w*, 2848*w*, 1725*m*, 1663*w*, 1630*m*, 1615*w*, 1576*w*, 1525*w*, 1501*w*, 1456*m*, 1433*m*, 1340*w*, 1319*w*, 1276*w*, 1250*m*, 1229*m*, 1176*w*, 1146*w*, 1130*w*, 1111*w*, 1095*w*, 1029*w*, 964*w*, 928*w*, 882*w*, 825*w*, 744*m*, 681*m*, 616*w*, 590*w*, 569*w*, 528*w*, 505*w*, 473*w*, 425*w* cm^−1^; UV-Vis (MeOH): λ_max_ (log ε) = 360.42 nm (4.22); ^1^H NMR (400 MHz, DMSO-d_6_): δ = 11.66 (*s*, 1H, NH), 9.43 (*s*, 1H, 23-H), 8.88 (*d*, *J* = 6.0 Hz, 1H, 27-H), 8.77 (*d*, *J* = 8.4 Hz, 1H, 25-H), 8.30 (*s*, 1H, 37-H), 8.12 (*dd*, *J* = 8.2, 6.0 Hz, 1H, 26-H), 8.04 (*d*, *J* = 7.7 Hz, 1H, 32-H), 7.89 (*d*, *J* = 16.5 Hz, 1H, 29-H), 7.47 (*d*, *J* = 7.5 Hz, 1H, 35-H), 7.20 (*d*, *J* = 16.5 Hz, 1H, 28-H), 7.27–7.10 (*m*, 2H, 33-H, 34-H), 5.01–4.79 (*m*, 2H, 22-H), 4.63 (*dt*, *J* = 12.3, 4.4 Hz, 1H, 21-H_a_), 4.47 (*dd*, *J* = 11.9, 3.5 Hz, 1H, 21-H_b_), 2.20 (*dd*, *J* = 18.4, 3.4 Hz, 1H, 15-H_a_), 1.94 (*d*, *J* = 12.8 Hz, 1H, 3-H_a_), 1.74 (*d*, *J* = 18.4 Hz, 1H, 15-H_b_), 1.60–1.40 (*m*, 5H, 1-H_a_, 2-H_a_, 6-H_a_, 7-H_a_, 11-H_a_), 1.38–1.16 (*m*, 6H, 2-H_b_, 7-H_b_, 12-H, 14-H), 1.10–1.00 (*m*, 2H, 5-H, 9-H), 1.04 (*s*, 3H, 19-H), 1.02–0.86 (*m*, 3H, 3-H_b_, 6-H_b_, 11-H_b_), 0.81 (*s*, 3H, 17-H), 0.85–0.73 (*m*, 1H, 1-H_b_), 0.41 (*s*, 3H, 20-H) ppm; ^13^C NMR (101 MHz, DMSO-d_6_): δ = 221.0 (C-16), 176.6 (C-18), 142.3 (C-23), 141.2 (C-27), 140.5 (C-25), 139.9 (C-36), 137.7 (C-24), 130.8 (C-29), 129.7 (C-37), 128.2 (C-26), 125.3 (C-31), 122.9 (C-28), 120.9 (C-34), 120.4 (C-32), 115.6 (C-33), 113.4 (C-30), 112.8 (C-35), 63.1 (C-21), 59.8 (C-22), 56.1 (C-5), 54.0 (C-9), 53.5 (C-14), 48.2 (C-13), 48.2 (C-15), 43.7 (C-4), 40.6 (C-7), 39.4 (C-1), 39.3 (C-8), 37.8 (C-10), 37.6 (C-3), 36.9 (C-12), 28.6 (C-19), 21.8 (C-6), 20.2 (C-11), 20.1 (C-17), 18.7 (C-2), 13.1 (C-20) ppm; MS (ESI, MeOH/DMSO, 4:1): *m*/*z* (%) 566 (60%, [M − Br]^+^); analysis calcd. for C_37_H_45_N_2_O_3_Br (645.67): C 68.83, H 7.02, N 4.34; found: C 68.64, H 7.19, N 4.21.

#### 4.2.15. 2-{2-[(E)-2-(1H-Indol-3-yl)ethenyl]-pyridinium-1-yl}-ethyl (4α)-13-Hydroxykaur-16-en-18-oate Bromide (**12**)

Following GPC from **6** (0.23 g, 0.54 mmol) and **5** (0.12 g, 0.54 mmol) followed by chromatography (SiO_2_, CHCl_3_/MeOH, 8:2), **12** (0.12 g, 40%) was obtained as a yellowish solid: *R*_f_ = 0.11 (SiO_2_, CHCl_3_/MeOH, 8.5:1.5); m.p. = 90–93 °C; ${\left[\alpha \right]}_{D}^{20}$ = −11.07° (*c* = 0.042, MeOH); IR (ATR): ν = 3386*br*, 2926*w*, 2854*w*, 1654*s*, 1495*w*, 1436*m*, 1420*m*, 1389*m*, 1351*m*, 1252*w*, 1166*w*, 1110*m*, 1061*w*, 823*w*, 666*w*, 481*br* cm^−1^; UV-Vis (MeOH): λ_max_ (log ε) = 433.17 nm (3.48); ^1^H NMR (500 MHz, DMSO-d_6_): δ = 12.17 (*s*, 1H, NH), 8.87 (*d*, *J* = 6.2 Hz, 1H, 23-H), 8.62 (*d*, *J* = 8.7 Hz, 1H, 26-H), 8.40 (*t*, *J* = 8.1 Hz, 2H, 25-H), 8.35 (*d*, *J* = 15.8 Hz, 1H, 29-H), 8.28 (*s*, 1H, 37-H), 8.13 (*d*, *J* = 7.2 Hz, 1H, 32-H), 7.76 (*t*, *J* = 6.4 Hz, 1H, 24-H), 7.52 (*d*, *J* = 7.2 Hz, 1H, 35-H), 7.40 (*d*, *J* = 15.6 Hz, 1H, 28-H), 7.28–7.17 (*m*, 2H, 33-H, 34-H), 5.26–5.07 (*m*, 2H, 22-H), 4.94 (*s*, 1H, 17-H_a_), 4.73 (*s*, 1H, 17-H_b_), 4.69–4.57 (*m*, 1H, 21-H_a_), 4.41–4.31 (*m*, 1H, 21-H_b_), 2.03–1.80 (*m*, 2H, 3-H_a_, 15-H), 1.74–1.36 (*m*, 6H, 1-H_a_,2-H_a_, 6-H_a_, 11-H_a_, 12-H_a_, 14-H_a_), 1.35–1.03 (*m*, 7H, 2-H_b_, 6-H_b_, 7-H, 11-H_b_, 12-H_b_, 14-H_b_), 0.98 (*s*, 3H, 19-H), 0.96–0.73 (*m*, 3H, 3-H_b_, 5-H, 9-H), 0.72–0.61 (*m*, 1H, 1-H_a_), 0.51 (*s*, 3H, 20-H) ppm; ^13^C NMR (126 MHz, DMSO-d_6_): δ = 176.9 (C-18), 154.5 (C-16), 145.6 (C-23), 145.1 (C-27), 144.1 (C-25), 139.3 (C-29), 137.8 (C-36), 132.9 (C-37), 125.8 (C-31), 124.6 (C-26), 123.5, 123.4 (C-24, 34), 121.8 (C-33), 121.4 (C-30), 120.6 (C-32), 114.0 (C-17), 113.1 (C-35), 110.3 (C-28), 81.2 (C-13), 62.2 (C-21), 56.0 (C-5), 55.5 (C-22), 53.5 (C-9), 47.7 (C-14, 15), 43.7 (C-4), 41.4 (C-8), 41.1 (C-7), 40.7 (C-1), 39.4 (C-10), 39.2 (C-12), 37.8 (C-3), 28.4 (C-19), 21.0 (C-6), 20.8 (C-11), 19.0 (C-2), 15.1 (C-20) ppm; MS (ESI, MeOH/DMSO, 4:1): *m*/*z* (%) 566 (55%, [M − Br]^+^); analysis calcd. For C_37_H_45_N_2_O_3_Br (645.67): C 68.83, H 7.02, N 4.34; found: C 68.63, H 7.27, N 4.19.

#### 4.2.16. 2-{2-[(E)-2-(1H-Indol-3-yl)ethenyl]-pyridinium-1-yl}-ethyl (4α, 8β, 13β) 13-Methyl-16-oxo-17-norkauran-18-oate Bromide (**13**) 

Following GPC from **7** (0.215 g, 0.51 mmol) and **5** (0.12 g, 0.54 mmol) followed by chromatography (SiO_2_, CHCl_3_/MeOH, 8:2), **13** (0.10 g, 40%) was obtained as a yellowish solid: *R*_f_ = 0.35 (SiO_2_, CHCl_3_/MeOH, 8.5:1.5); m.p. = 94–97 °C; ${\left[\alpha \right]}_{D}^{20}$ = −12.83° (*c* = 0.073, MeOH); IR (ATR): ν = 3368*br*, 2923*m*, 2850*m*, 1725*s*, 1629*w*, 1604*s*, 1562*m*, 1496*m*, 1446*br*, 1433*m*, 1375*w*, 1318*w*, 1280*w*, 1244*m*, 1209*w*, 1166*w*, 1146*w*, 1131*m*, 1112*w*, 1095*w*, 1056*m*, 1040*w*, 966*w*, 928*w*, 818*w*, 745*m*, 665*w*, 570*w*, 508*w*, 423*w* cm^−1^; UV-Vis (MeOH): λ_max_ (log ε) = 434.17 nm (4.02); ^1^H NMR (500 MHz, DMSO-d_6_): δ =12.14 (*s*, 1H, NH), 8.83 (*d*, *J* = 5.5 Hz, 1H, 23-H), 8.61 (*d*, *J* = 7.9 Hz, 1H, 26-H), 8.41 (*t*, *J* = 7.6 Hz, 1H, 25-H), 8.35 (*d*, *J* = 15.6 Hz, 1H, 29-H), 8.32 (*s*, 1H, 37-H), 8.12 (*d*, *J* = 7.2 Hz, 1H, 32-H), 7.76 (*t*, *J* = 6.8 Hz, 1H, 24-H), 7.52 (*d*, *J* = 7.0 Hz, 1H, 35-H), 7.38 (*d*, *J* = 15.7 Hz, 1H, 28-H), 7.30–7.20 (*m*, 2H, 33-H, 34-H), 5.25–4.95 (*m*, 2H, 22-H), 4.65–4.53 (*m*, 1H, 21-H_a_), 4.43–4.30 (*m*, 1H, 21-H_b_), 2.20 (*dd*, *J* = 18.4, 3.6 Hz, 1H, 15-H_a_), 1.91 (*d*, *J* = 12.8 Hz, 1H, 3-H_a_), 1.73 (*d*, *J* = 18.5 Hz, 1H, 15-H_b_), 1.60–1.48 (*m*, 6H, 1-H_b_, 2-H, 6-H_b_, 7-H_b_, 11-H_b_), 1.46–1.36 (*m*, 4H, 7-H_a_, 12-H, 14-H_b_), 1.32 (*d*, *J* = 7.7 Hz, 2H, 11-H_a_, 14-H_a_), 1.17–1.04 (*m*, 2H, 5-H, 9-H), 1.03 (*s*, 2H, 3-H_b_, 6-H_a_), 0.99 (*s*, 3H, 19-H), 0.87 (*s*, 1H, 1-H_a_), 0.85 (*s*, 3H, 17-H), 0.39 (*s*, 3H, 20-H) ppm; ^13^C NMR (126 MHz, DMSO-d_6_): δ = 221.1 (C-16), 176.8 (C-18), 154.4 (C-27), 145.5 (C-23), 145.4 (C-37), 144.0 (C-25), 139.2 (C-29), 137.7 (C-36), 125.7 (C-31), 124.4 (C-26), 123.4 (C-34), 123.2 (C-24), 121.8 (C-33), 120.4 (C-32), 119.6, 113.9 (C-30), 113.1 (C-35), 110.1 (C-28), 62.2 (C-21), 56.4 (C-22), 56.1 (C-5), 54.1 (C-9), 53.6 (C-14), 48.3 (C-13), 48.2 (C-15), 43.6 (C-4), 40.7 (C-7), 39.4 (C-1), 39.3 (C-8), 37.8 (C-10), 37.7 (C-3), 37.0 (C-12), 28.4 (C-19), 21.7 (C-6), 20.2 (C-17), 18.9 (C-11), 18.8 (C-2), 13.0 (C-20) ppm; MS (ESI, MeOH/DMSO, 4:1): *m*/*z* (%) 566 (65%, [M − Br]^+^; analysis calcd. for C_37_H_45_N_2_O_3_Br (645.67): C 68.83, H 7.02, N 4.34; found: C 68.60, H 7.19, N 4.08.

#### 4.2.17. 3-Bromopropyl (4α) -13-hydroxykaur-16-en-18-oate (**14**)

Following GPB from **1** (0.5 g, 1.57 mmol), K_2_CO_3_ (0.434 g, 3.1 mmol) and 1,3-dibromopropane (0.64 mL, 6.3 mmol) followed by chromatography (SiO_2_, hexanes/ethyl acetate, 6:1), **14** (0.3 g, 44%) was obtained as a colorless solid: *R*_f_ = 0.3 (SiO_2_, hexanes/ethyl acetate, 8:2); m.p. = 97–99 °C; ${\left[\alpha \right]}_{D}^{20}$ = −56.12° (*c* = 0.104, CHCl_3_); IR (ATR): ν = 3493*m*, 2990*w*, 2974*w*, 2958*w*, 2939*m*, 2851*m*, 1703*s*, 1469*w*, 1458*w*, 1444*w*, 1392*w*, 1369*w*, 1330*m*, 1275*w*, 1237*m*, 1220*w*, 1201*w*, 1178*w*, 1155*w*, 1119*m*, 1097*w*, 1018*w*, 1001*m*, 967*w*, 952*w*, 938*w*, 891*w*, 875*w*, 849*w*, 813*w*, 770*w*, 694*w*, 620*w*, 571*w*, 518*w*, 420*w* cm^−1^; ^1^H NMR (400 MHz, CDCl_3_): δ = 4.99–4.95 (*m*, 1H, 17-H_a_), 4.83–4.79 (*m*, 1H, 17-H_b_), 4.26–4.18 (*m*, 1H, 21-H_a_), 4.17–4.08 (*m*, 1H, 21-H_b_), 3.48 (*t*, *J* = 6.5 Hz, 2H, 23-H), 2.23–2.00 (*m*, 6H, 3-H_a_, 14-H_a_, 15-H, 22-H), 1.89–1.79 (*m*, 3H, 1-H_a_, 2-H_a_ 6-H_a_), 1.79–1.71 (*m*, 3H, 6-H_b_, 11-H_a_, 12-H_a_), 1.62–1.55 (*m*, 1H, 11-H_b_), 1.55–1.48 (*m*, 2H, 7-H_a_, 12-H_b_), 1.48–1.35 (*m*, 2H, 2-H_b_, 7-H_b_), 1.29–1.23 (*m*, 1H, 14-H_b_), 1.17 (*d*, *J* = 1.7 Hz, 3H, 19-H), 1.08–0.93 (*m*, 3H, 3-H_b_, 5-H, 9-H), 0.85 (*d*, *J* = 2.3 Hz, 3H, 20-H), 0.80 (*dd*, *J* = 13.4, 4.8 Hz, 1H, 1-H_b_) ppm; ^13^C NMR (101 MHz, CDCl_3_): δ = 177.3 (C-18), 156.0 (C-16), 102.9 (C-17), 82.6, 80.2 (C-13), 61.8, 56.9 (C-5), 53.7 (C-9), 50.9, 47.4 (C-15), 47.0 (C-14), 43.9 (C-4), 41.6 (C-8), 41.3 (C-7), 40.6 (C-1), 39.3 (C-10), 39.2 (C-12), 38.0 (C-3), 31.5 (C-22), 29.6 (C-23), 28.8 (C-19), 21.9 (C-6), 20.4 (C-11), 19.1 (C-2), 15.5 (C-20) ppm; MS (ESI, MeOH:CHCl_3_ 4:1): *m*/*z* (%) 359 (90%, [M − Br]^+^); analysis calcd. for C_23_H_35_O_3_Br (439.43): C 62.87, H 8.03; found: C 62.77, H 8.20.

#### 4.2.18. 3-Bromopropyl (4α, 8β, 13β) 13-Methyl-16-oxo-17-norkauran-18-oate (**15**)

Following GPB from **2** (0.5 g, 1.57 mmol), K_2_CO_3_ (0.434 g, 3.1 mmol) and 1,3-dibromopropane (0.64 mL, 6.3 mmol) followed by chromatography (SiO_2_, hexanes/ethyl acetate, 6:1), **15** (0.42 g, 61%) was obtained as a colorless solid: *R*_f_ = 0.69 (SiO_2_, hexanes/ethyl acetate,8:2); m.p. = 103–106 °C; ${\left[\alpha \right]}_{D}^{20}$ = −58.31° (*c* = 0.15, CHCl_3_); IR (ATR): ν = 2932*m*, 2899*w*, 2884*w*, 2854*w*, 2839*w*, 1720*s*, 1470*m*, 1457*m*, 1423*w*, 1389*w*, 1369*w*, 1332*m*, 1321*w*, 1288*m*, 1257*m*, 1233*m*, 1219*w*, 1209*w*, 1179*s*, 1149*s*, 1135*m*, 1110*m*, 1097*m*, 1062*w*, 1018*m*, 978*m*, 942*w*, 929*w*, 895*w*, 876*w*, 852*w*, 827*w*, 767*m*, 738*w*, 696*w*, 660*w*, 632*w*, 612*w*, 587*m*, 532*w*, 507*w*, 462*w*, 436*w*, 418*w* cm^−1^; ^1^H NMR (400 MHz, CDCl_3_): δ = 4.25–4.17 (*m*, 1H, 21-H_a_), 4.15–4.06 (*m*, 1H, 21-H_b_), 3.46 (*t*, *J* = 6.5 Hz, 2H, 23-H), 2.62 (*dd*, *J* = 18.6, 3.8 Hz, 1H, 15-H_a_), 2.16 (*p*, *J* = 6.2 Hz, 3H, 3-H_a_, 22-H), 1.92–1.84 (*m*, 1H, 6-H_a_), 1.85–1.78 (*m*, 1H, 2-H_a_), 1.79 (*d*, *J* = 18.7 Hz, 1H, 15-H_b_), 1.74–1.60 (*m*, 5H, 1-H_a_, 6-H_b_, 7-H_a_, 11-H), 1.61–1.49 (*m*, 2H, 12-H_a_, 14-H_a_), 1.48–1.31 (*m*, 4H, 2-H_b_, 7-H_b_, 12-H_b_, 14-H_b_), 1.29–1.21 (*m*, 1H, 9-H), 1.19 (*s*, 3H, 19-H), 1.13 (*dd*, *J* = 12.0, 2.1 Hz, 1H, 5-H), 1.02 (*td*, *J* = 13.5, 4.2 Hz, 1H, 3-H_b_), 0.96 (*s*, 3H, 17-H), 0.90 (*td*, *J* = 13.1, 4.2 Hz, 1H, 1-H_b_), 0.70 (*s*, 3H, 20-H) ppm; ^13^C NMR (101 MHz, CDCl_3_): δ = 222.3 (C-16), 177.1 (C-18), 61.8 (C-21), 57.0 (C-5), 54.7, 54.3 (C-14), 48.7 (C-13), 48.4 (C-15), 43.9 (C-4), 41.5 (C-7), 39.8 (C-1), 39.4 (C-8), 38.0 (C-10), 37.9 (C-3), 37.3 (C-12), 31.4 (C-22), 29.6 (C-23), 28.9 (C-19), 21.7 (C-6), 20.3 (C-11), 19.8 (C-17), 18.9 (C-2), 13.4 (C-20) ppm; MS (ESI, MeOH/CHCl_3_, 4:1): *m*/*z* (%) 359 (80%, [M − Br]^+^); analysis calcd. for C_23_H_35_O_3_Br (439.43): C 62.87, H 8.03; found: C 62.66, H 8.24.

#### 4.2.19. 3-{4-[(E)-2-(1H-Indol-3-yl)ethenyl]-pyridinium-1-yl}-propyl (4α)-13-Hydroxykaur-16-en-18-oate Bromide (**16**)

Following GPC from **14** (0.155 g, 0.35 mmol) and **3** (0.08 g, 0.36 mmol) followed by chromatography (SiO_2_, CHCl_3_/MeOH, 6:1), **16** (0.161 g, 79%) was obtained as a reddish solid: *R*_f_ = 0.84 (SiO_2_, CHCl_3_/MeOH, 9:1); m.p. = 209–211 °C; ${\left[\alpha \right]}_{D}^{20}$ = −20.78° (*c* = 0.074, MeOH); IR (ATR): ν = 3316*w*, 3163*w*, 2935*w*, 1720*m*, 1607*s*, 1575*m*, 1503*m*, 1462*m*, 1433*m*, 1362*w*, 1330*w*, 1251*m*, 1204*w*, 1180*m*, 1165*w*, 1142*m*, 1104*m*, 1086*w*, 1049*w*, 1038*w*, 960*m*, 873*m*, 832*w*, 745*s*, 608*w*, 570*w*, 513*w*, 429*w* cm^−1^; UV-Vis (MeOH): λ_max_ (log ε) = 445.33 nm (4.59); ^1^H NMR (500 MHz, DMSO-d_6_): δ = 11.98 (*s*, 1H, NH), 8.78 (*d*, *J* = 6.8 Hz, 2H, 25-H, 27-H), 8.28 (*d*, *J* = 16.3 Hz, 1H, 30-H), 8.19–8.12 (*m*, 3H, 24-H, 28-H, 33-H), 7.98 (*d*, *J* = 2.8 Hz, 1H, 38-H), 7.51 (*d*, *J* = 7.2 Hz, 1H, 36-H), 7.31 (*d*, *J* = 16.1 Hz, 1H, 29-H), 7.29–7.19 (*m*, 2H, 34-H, 35-H), 4.92–4.86 (*m*, 1H, 17-H_a_), 4.70–4.65 (*m*, 1H, 17-H_b_), 4.51 (*t*, *J* = 7.0 Hz, 2H, 23-H), 4.17–3.99 (*m*, 2H, 21-H), 2.32–2.23 (*m*, 2H, 22-H), 1.99 (*dd*, *J* = 26.6, 9.8 Hz, 3H, 3-H_a_, 15-H), 1.86 (*dd*, *J* = 10.8, 1.1 Hz, 1H, 14-H_b_), 1.80–1.55 (*m*, 6H, 1-H_a_, 2-H_a_, 6-H, 11-H_a_, 12-H_a_), 1.55–1.39 (*m*, 2H, 7-H_a_, 11-H_b_), 1.34 (*s*, 3H, 2-H_b_, 7-H_b_, 12-H_b_), 1.20 (*dd*, *J* = 11.1, 1.7 Hz, 1H, 14-H_a_), 1.08 (*s*, 3H, 19-H), 1.03 (*dd*, *J* = 11.9, 1.5 Hz, 1H, 5-H), 0.95 (*td*, *J* = 13.5, 4.1 Hz, 1H, 3-H_b_), 0.90 (*d*, *J* = 7.9 Hz, 1H, 9-H), 0.80–0.76 (*m*, 1H, 1-H_b_), 0.74 (*s*, 3H, 20-H) ppm; ^13^C NMR (126 MHz, DMSO-d_6_): δ = 176.9 (C-18), 156.7 (C-16), 145.0 (C-26), 143.9 (C-25, C-27), 138.0 (C-37), 137.2 (C-30), 133.0 (C-38), 125.4 (C-32), 123.4 (C-34), 122.4 (C-24, C-28), 121.6 (C-35), 120.9 (C-33), 117.2 (C-29), 114.1 (C-31), 113.1 (C-36), 103.0 (C-17), 81.2 (C-13), 61.4 (C-21), 57.2 (C-23), 56.4 (C-5), 53.6 (C-9), 47.7 (C-15), 46.6 (C-14), 43.7 (C-4), 41.5 (C-8), 41.3 (C-7), 40.4 (C-1), 39.5 (C-10), 39.2 (C-12), 37.8 (C-3), 29.9 (C-22), 28.7 (C-19), 22.0 (C-6), 20.4 (C-11), 19.2 (C-2), 15.7 (C-20) ppm; MS (ESI, MeOH/DMSO, 4:1): *m*/*z* (%) 580 (80%, [M − Br]^+^); analysis calcd. for C_38_H_47_N_2_O_3_Br (659.70): C 69.18, H 7.18, N 4.25; found: C 68.88, H 7.30, N 4.03.

#### 4.2.20. 3-{4-[(E)-2-(1H-Indol-3-yl)ethenyl]-pyridinium-1-yl}-propyl (4α, 8β, 13β) 13-Methyl-16-oxo-17-norkauran-18-oate Bromide (**17**)

Following GPC from **15** (0.212 g, 0.48 mmol), **3** (0.11 g, 0.05 mmol) followed by chromatography (SiO_2_, CHCl_3_/MeOH, 6:1), **17** (0.246 g, 88%) was obtained as a reddish solid: *R*_f_ = 0.85 (SiO_2_, CHCl_3_/MeOH, 9:1); m.p. = 184–186 °C; ${\left[\alpha \right]}_{D}^{20}$ = −15.67° (*c* = 0.052, MeOH); IR (ATR): ν = 2924*w*, 2847*w,* 1722*m*, 1644*w*, 1598*s*, 1574*m*, 1500*m*, 1457*w*, 1432*m*, 1371*w*, 1355*w*, 1317*w*, 1275*w*, 1246*m*, 1205*m*, 1174*s*, 1131*s*, 1096*w*, 1043*w*, 965*w*, 871*w*,745*s*, 663*w*, 611*w*, 568*w*, 512*w*, 425*w* cm^−1^; UV-Vis (MeOH): λ_max_ (log ε) = 444.49 nm (4.70); ^1^H NMR (400 MHz, DMSO-d_6_): δ = 11.94 (*s*, 1H, NH), 8.75 (*d*, *J* = 6.9 Hz, 2H, 24-H, 28-H), 8.25 (*d*, *J* = 16.2 Hz, 1H, 30-H), 8.14 (*d*, *J* = 6.9 Hz, 3H, 25-H, 27-H, 33-H), 7.96 (*d*, *J* = 2.7 Hz, 1H, 38-H), 7.50 (*d*, *J* = 7.0 Hz, 1H, 36-H), 7.29 (*d*, *J* = 16.1 Hz, 1H, 29-H), 7.26–7.18 (*m*, 2H, 34-H, 35-H), 4.48 (*t*, *J* = 7.0 Hz, 2H, 23-H), 4.17–3.96 (*m*, 2H, 21-H), 2.41 (*d*, *J* = 18.4 Hz, 1H, 15-H_a_), 2.31–2.21 (*m*, 2H, 22-H), 1.96 (*td*, *J* = 12.5, 2.8 Hz, 1H, 3-H_a_), 1.87 (*d*, *J* = 18.5 Hz, 1H, 6-H_a_), 1.72 (*td*, *J* = 13.3, 2.4 Hz, 1H, 15-H_b_), 1.68–1.54 (*m*, 5H, 1-H_a_, 2-H_a_, 6-H_b_, 7-H_a_, 11-H_a_), 1.54–1.44 (*m*, 2H, 12-H_a_, 14-H_a_), 1.44–1.29 (*m*, 4H, 2-H_b_, 7-H_b_, 12-H_b_, 14-H_b_), 1.23–1.11 (*m*, 3H, 5-H, 9-H, 11-H_b_), 1.09 (*s*, 3H, 19-H), 0.97 (*td*, *J* = 13.5, 4.2 Hz, 1H, 3-H_b_), 0.92–0.87 (*m*, 1H, 1-H_b_), 0.85 (*s*, 3H, 17-H), 0.60 (*s*, 3H, 20-H) ppm; ^13^C NMR (101 MHz, DMSO-d_6_): δ = 220.8 (C-16), 176.4 (C-18), 154.8 (C-26), 143.4 (C-24, C-28), 137.5 (C-37), 136.7 (C-30), 132.5 (C-38), 124.9 (C-32), 123.0 (C-29), 121.9 (C-25, C-27), 121.2 (C-35), 120.4 (C-34), 116.8 (C-33), 113.6 (C-31), 112.6 (C-36), 61.0 (C-21), 56.8 (C-23), 55.9 (C-5), 53.7 (C-9), 53.1 (C-14), 47.9 (C-15), 47.7 (C-13), 43.2 (C-4), 40.1 (C-7), 39.0 (C-1), 38.9 (C-8), 37.4 (C-3), 37.2 (C-10), 36.6 (C-12), 29.4 (C-22), 28.3 (C-19), 21.3 (C-6), 19.8 (C-11), 19.7 (C-17), 18.5 (C-2), 13.0 (C-20) ppm; MS (ESI, MeOH/DMSO, 4:1): *m*/*z* (%) 580 (80%, [M − Br]^+^); analysis calcd. for C_38_H_47_N_2_O_3_Br (659.70): C 69.18, H 7.18, N 4.25; found: C 68.89, H 7.33, N 4.02.

#### 4.2.21. 3-{3-[(E)]-2-(1H-Indol-3-yl)-ethenyl]-pyridinium-1-yl}-propyl (4α)-13-Hydroxykaur-16-en-18-oate Bromide (**18**)

Following GPC from **14** (0.305 g, 0.7 mmol) and **4** (0.154 g, 0.7 mmol) followed by chromatography (SiO_2_, CHCl_3_/MeOH, 6:1), **18** (0.366 g, 91%) was obtained as a yellowish solid: *R*_f_ = 0.13 (SiO_2_, CHCl_3_/MeOH, 9:1); m.p. = 175–177 °C; ${\left[\alpha \right]}_{D}^{20}$ = −43.33° (*c* = 0.111, MeOH); IR (ATR): ν = 2932*br*, 2850*w,* 1713*m* 1631*m*, 1577*m*, 1525*w*, 1501*w*, 1459*w*, 1432*m*, 1365*w*, 1331*w*, 1277*w*, 1235*m*, 1148*m*, 1096*w*, 1055*w*, 1020*w*, 958*w*, 880*w*, 818*w*, 745*m*, 679*w*, 568*w*, 527*w*, 501*w*, 426*w* cm^−1^; UV-Vis (MeOH): λ_max_ (log ε) = 358.18 nm (4.66); ^1^H NMR (500 MHz, DMSO-d_6_): δ = 11.66 (*s*, 1H, NH), 9.35 (*s*, 1H, 28-H), 8.83 (*d*, *J* = 6.0 Hz, 1H, 24-H), 8.75 (*d*, *J* = 8.4 Hz, 1H, 26-H), 8.12–8.04 (*m*, 2H, 25-H, 33-H), 7.89 (*d*, *J* = 16.6 Hz, 1H, 30-H), 7.78 (*d*, *J* = 2.7 Hz, 1H, 38-H), 7.48 (*d*, *J* = 7.5 Hz, 1H, 36-H), 7.22 (*d*, *J* = 16.5 Hz, 1H, 29-H), 7.24–7.14 (*m*, 2H, 34-H, 35-H), 4.87 (*s*, 1H, 17-H_a_), 4.69 (*d*, *J* = 7.2 Hz, 3H, 17-H_b_, 23-H), 4.21–4.03 (*m*, 2H, 21-H), 2.42–2.33 (*m*, 2H, 22-H), 2.05–1.90 (*m*, 3H, 3-H_a_, 15-H), 1.85 (*d*, *J* = 11.0 Hz, 1H, 14-H_b_), 1.79–1.65 (*m*, 3H, 1-H_a_, 2-H_a_, 6-H_a_), 1.65–1.49 (*m*, 3H, 6-H_b_, 11-H_a_, 12-H_a_), 1.50–1.39 (*m*, 2H, 7-H_a_, 11-H_b_), 1.33 (*s*, 3H, 2-H_b_, 7-H_b_, 12-H_b_), 1.19 (*dd*, *J* = 10.8, 1.6 Hz, 1H, 14-H_a_), 1.06 (*s*, 3H, 19-H), 1.03 (*dd*, *J* = 12.0, 1.7 Hz, 1H, 5-H), 0.95 (*td*, *J* = 13.5, 4.2 Hz, 1H, 3-H_b_), 0.89 (*d*, *J* = 8.1 Hz, 1H, 9-H), 0.79–0.76 (*m*, 1H, 1-H_b_), 0.75 (*s*, 3H, 20-H) ppm; ^13^C NMR (126 MHz, DMSO-d_6_): δ =176.9 (C-18), 156.7 (C-16), 142.0 (C-28), 141.1 (C-24), 140.1 (C-37), 140.0 (C-26), 137.8 (C-27), 130.7 (C-30), 129.7 (C-38), 128.2 (C-25), 125.3 (C-32), 122.9 (C-29), 120.9 (C-35), 120.5 (C-33), 115.8 (C-34), 113.4 (C-31), 112.8 (C-36), 103.0 (C-17), 79.1 (C-13), 61.5 (C-21), 59.1 (C-23), 56.4 (C-5), 53.6 (C-9), 47.7 (C-15), 46.6 (C-14), 43.6 (C-4), 41.4 (C-8), 41.3 (C-7), 40.5 (C-1), 39.5 (C-10), 39.2 (C-12), 37.8 (C-3), 30.0 (C-22), 28.7 (C-19), 22.0 (C-6), 20.4 (C-11), 19.2 (C-2), 15.7 (C-20) ppm; MS (ESI, MeOH/DMSO, 4:1): *m*/*z* (%) 580 (63%, [M − Br]^+^); analysis calcd. for C_38_H_47_N_2_O_3_Br (659.70): C 69.18, H 7.18, N 4.25; found: C 68.88, H 7.37, N 3.99.

#### 4.2.22. 3-{3[(E)-2-(1H-Indol-3-yl)-ethenyl]-pyridinium-1-yl}-propyl (4α, 8β, 13β) 13-Methyl-16-oxo-17-norkauran-18-oate Bromide (**19**)

Following GPC from **15** (0.423 g, 0.96 mmol) and **4** (0.212 g, 0.96 mmol) followed by chromatography (SiO_2_, CHCl_3_/MeOH, 6:1), **19** (0.418 g, 75%) was obtained as a yellowish solid: *R*_f_ = 0.27 (SiO_2_, CHCl_3_/MeOH 9:1); m.p. = 171–173 °C; ${\left[\alpha \right]}_{D}^{20}$ = −29.97° (*c* = 0.109, MeOH); IR (ATR): ν = 3164*br*, 3058*w*, 2937*m*, 2847*w,* 2454*w*, 1732*m*, 1718*s*, 1632*m*, 1584*m*, 1526*w*, 1500*m*, 1456*m*, 1427*m*, 1374*w*, 1336*w*, 1321*w*, 1273*w*, 1246*m*, 1225*m*, 1159*s*, 1130*m*, 1108*m*, 1056*w*, 1016*w*, 976*w*, 953*m*, 926*w*, 905*w*, 873*w*, 822*w*, 807*w*, 746*s*, 678*m*, 662*m*, 610*w*, 590*w*, 567*m*, 508*w*, 441*w*, 423*m* cm^−1^; UV-Vis (MeOH): λ_max_ (log ε) = 358.18 nm (4.02); ^1^H NMR (500 MHz, DMSO-d_6_): δ = 11.65 (*s*, 1H, NH), 9.32 (*s*, 1H, 28-H), 8.81 (*d*, *J* = 6.0 Hz, 1H, 26-H), 8.75 (*d*, *J* = 8.3 Hz, 1H, 24-H), 8.12–8.04 (*m*, 2H, 25-H, 33-H), 7.88 (*d*, *J* = 16.5 Hz, 1H, 30-H), 7.77 (*d*, *J* = 2.6 Hz, 2H, 38-H), 7.48 (*d*, *J* = 7.9 Hz, 1H, 36-H), 7.22 (*d*, *J* = 16.3 Hz, 1H, 29-H), 7.23–7.13 (*m*, 1H, 34-H, 35-H), 4.66 (*t*, *J* = 6.9 Hz, 2H, 23-H), 4.12 (*m*, 2H, 21-H), 2.46–2.34 (*m*, 3H, 15-H_a_, 22-H), 1.95 (*d*, *J* = 13.0 Hz, 1H, 3-H_a_), 1.84 (*d*, *J* = 18.3 Hz, 1H, 15-H_b_), 1.73–1.49 (*m*, 6H, 1-H_a_, 2-H_a_, 6-H, 7-H_a_, 11-H_b_), 1.49–1.26 (*m*, 6H, 7-H_b_, 11-H_a_, 12-H, 14-H), 1.19–1.04 (*m*, 3H, 2-H_b_, 5-H, 9-H), 1.08 (*s*, 3H, 19-H), 0.98 (*td*, *J* = 13.5, 3.9 Hz, 1H, 3-H_b_), 0.88 (*td*, *J* = 13.4, 3.7 Hz, 1H, 1-H_b_), 0.83 (*s*, 3H, 17-H), 0.61 (*s*, 3H, 20-H) ppm; ^13^C NMR (126 MHz, DMSO-d_6_): δ = 221.2 (C-16), 176.9 (C-18), 142.0 (C-28), 141.2 (C-26), 140.1 (C-37), 140.0 (C-24), 137.8 (C-27), 130.7 (C-30), 129.7 (C-38), 128.2 (C-25), 125.3 (C-32), 122.9 (C-29), 120.9 (C-35), 120.4 (C-33), 115.8 (C-34), 113.4 (C-31), 112.8 (C-36), 61.6 (C-21), 59.3 (C-23), 56.4 (C-5), 54.1 (C-9), 53.6 (C-14), 48.3 (C-13), 48.1 (C-15), 43.6 (C-4), 40.9 (C-7), 40.5 (C-1), 39.5 (C-8), 39.4 (C-10), 37.9 (C-3), 37.7 (C-12), 29.9 (C-22), 28.7 (C-19), 21.8 (C-6), 20.3 (C-2), 20.2 (C-17), 19.0 (C-11), 13.5 (C-20) ppm; MS (ESI, MeOH/DMSO, 4:1): *m*/*z* (%) 580 (64%, [M − Br]^+^); analysis calcd. for C_38_H_47_N_2_O_3_Br (659.70): C 69.18, H 7.18, N 4.25; found: C 68.96, H 4.45, N 4.02.

#### 4.2.23. 3-{2-[(E)-2-(1H-Indol-3-yl)-ethenyl]-pyridinium-1-yl}-propyl (4α)-13-Hydroxykaur-16-en-18-oate bromide (**20**)

Following GPC from **14** (0.305 g, 0.7 mmol) and **5** (0.154 g, 0.7 mmol) followed by chromatography (SiO_2_, CHCl_3_/MeOH, 4:1), **21** (0.12 g, 30%) was obtained as a yellowish solid: *R*_f_ = 0.2 (SiO_2_, CHCl_3_/MeOH, 8.5:1.5); m.p. = 91–94 °C; ${\left[\alpha \right]}_{D}^{20}$ = −31.96° (*c* = 0.097, MeOH); IR (ATR): ν = 3370*br*, 2925*w*, 2851*w,* 1716*w*, 1628*w*, 1605*m*, 1564*m*, 1499*m*, 1461*w*, 1431*m*, 1364*w*, 1325*w*, 1274*w*, 1243*m*, 1164*w*, 1152*w*, 1135*w*, 1116*w*, 1051*w*, 955*w*, 816*w*, 745*m*, 520*br*, 423*w* cm^−1^; UV-Vis (MeOH): λ_max_ (log ε) = 427.47 nm (4.24); ^1^H NMR (400 MHz, DMSO-d_6_): δ =12.14 (*s*, 1H, NH), 8.81 (*d*, *J* = 6.1 Hz, 1H, 24-H), 8.57 (*d*, *J* = 8.4 Hz, 1H, 27-H), 8.37 (*t*, *J* = 7.7 Hz, 1H, 26-H), 8.32 (*d*, *J* = 16.1 Hz, 1H, 30-H), 8.21 (*s*, 1H, 38-H), 8.06 (*d*, *J* = 7.7 Hz, 1H, 33-H), 7.74 (*t*, *J* = 6.6 Hz, 1H, 25-H), 7.50 (*d*, *J* = 7.8 Hz, 1H, 36-H), 7.31–7.14 (*m*, 2H, 34-H, 35-H), 7.23 (*d*, *J* = 15.2 Hz, 1H, 29-H), 4.94 (*s*, 1H, 17-H_a_), 4.84 (*t*, 2H, 23-H), 4.56 (*s*, 1H, 17-H_b_), 4.23–4.03 (*m*, 2H, 21-H), 2.33–2.12 (*m*, 2H, 22-H), 1.99–1.70 (*m*, 4H, 3-H_a_, 14-H, 15-H_a_), 1.69–1.31 (*m*, 10H, 1-H_a_, 2-H_a_, 6-H, 7-H, 11-H, 12-H), 1.32–1.04 (*m*, 2H, 2-H_b_, 15-H_b_), 0.99 (*s*, 3H, 19-H), 0.97–0.69 (*m*, 4H, 1-H_b_, 3-H_b_, 5-H, 9-H), 0.67 (*s*, 3H, 20-H) ppm; ^13^C NMR (101 MHz, DMSO-d_6_): δ = 176.8 (C-18), 153.8 (C-16), 145.1 (C-24), 144.9 (C-28), 143.8 (C-26), 139.1 (C-30), 137.7 (C-37), 133.6, 132.8 (C-38), 125.6 (C-32), 124.7 (C-27), 123.5 (C-25), 123.3 (C-35), 121.7 (C-34), 120.3 (C-33), 113.8 (C-31), 113.1 (C-36), 110.1 (C-29), 102.9 (C-17), 81.2 (C-13), 61.6 (C-21), 56.7 (C-5), 56.1 (C-9), 55.4 (C-23), 50.9 (C-15), 49.0, 47.6 (C-14), 46.7, 43.6 (C-8), 41.6 (C-7), 39.4 (C-1), 38.4 (C-10), 37.8 (C-3), 37.8, 32.2, 29.4 (C-22), 28.7 (C-19), 22.5 (C-6), 20.8 (C-11), 19.1 (C-2), 15.4 (C-20) ppm; MS (ESI, MeOH/DMSO, 4:1): *m*/*z* (%) 580 (90%, [M − Br]^+^); analysis calcd. For C_38_H_47_N_2_O_3_Br (659.70): C 69.18, H 7.18, N 4.25; found: C 68.96, H 7.34, N 4.13.

#### 4.2.24. 3-{2-[(E)-2-(1-H-Indol-3-yl)-ethenyl]-pyridinium-1-yl}-propyl (4α)-13-Hydroxykaur-16-en-18-oate Bromide (**21**)

Following GPC from **15** (0.371 g, 0.72 mmol) and **5** (0.158 g, 0.72 mmol) followed by chromatography (SiO_2_, CHCl_3_/MeOH, 6:1), **20** (0.174 g, 36%) was obtained as a yellowish solid: *R*_f_ = 0.1 (SiO_2_, CHCl_3_/MeOH, 9:1); m.p. = 89–92 °C; ${\left[\alpha \right]}_{D}^{20}$ = −57.04° (*c* = 0.301, MeOH); IR (ATR): ν = 3360*br*, 2925*w*, 2848*w,* 1721*m*, 1628*w*, 1605*m*, 1564*m*, 1524*w*, 1499*m*, 1449*w*, 1432*m*, 1374*w*, 1318*w*, 1274*w*, 1237*m*, 1156*m*, 1131*m*, 1112*w*, 1096*w*, 1057*w*, 1039*w*, 1016*w*, 966*w*, 877*w*, 853*w*, 816*w*, 745*s*, 663*w*, 567*w*, 508*w*, 423*w* cm^−1^; UV-Vis (MeOH): λ_max_ (log ε) = 430.32 nm (4.24); ^1^H NMR (500 MHz, DMSO-d_6_): δ = 12.08 (*s*, 1H, NH), 8.77 (*d*, *J* = 6.2 Hz, 1H, 24-H), 8.57 (*d*, *J* = 8.1 Hz, 1H, 27-H), 8.38 (*dd*, *J* = 7.8 Hz, 1H, 26-H), 8.31 (*d*, *J* = 15.6 Hz, 1H, 30-H), 8.19 (*d*, *J* = 2.9 Hz, 1H, 38-H), 8.07 (*d*, *J* = 7.6 Hz, 1H, 33-H), 7.75 (*t*, *J* = 6.6 Hz, 1H, 25-H), 7.51 (*d*, *J* = 7.6 Hz, 1H, 36-H), 7.27 (*d*, *J* = 28.6 Hz, 1H, 29-H), 7.28–7.17 (*m*, 2H, 34-H, 35-H), 4.89–4.76 (*m*, 2H, 23-H), 4.23–4.09 (*m*, 2H, 21-H), 2.33–2.22 (*m*, 3H, 15-H_a_, 22-H), 1.93 (*d*, *J* = 13.2 Hz, 1H, 3-H_a_), 1.70–1.42 (*m*, 7H, 1-H_a_, 6-H, 7-H_a_, 11-H_a_, 15-H_b_), 1.43–1.20 (*m*, 6H, 2-H, 7-H_b_, 12-H, 14-H), 1.20–1.03 (*m*, 2H, 5-H, 9-H), 1.01 (*s*, 3H, 19-H), 1.00–0.86 (*m*, 3H, 1-H_b_, 3-H_b_, 11-H_b_), 0.83 (*s*, 3H, 17-H), 0.53 (*s*, 3H, 20-H) ppm; ^13^C NMR (126 MHz, DMSO-d_6_): δ = 221.0 (C-16), 176.9 (C-18), 153.8 (C-28), 145.1 (C-24), 143.8 (C-26), 139.1 (C-30), 137.8 (C-37), 132.6 (C-38), 125.7 (C-32), 124.6 (C-27), 123.6 (C-25), 123.4 (C-35), 121.7 (C-34), 120.3 (C-33), 113.9 (C-31), 113.1 (C-36), 110.2 (C-29), 61.8 (C-21), 56.4 (C-5), 55.6 (C-23), 54.1 (C-9), 53.6 (C-14), 48.3 (C-13), 47.9 (C-15), 43.6 (C-4), 40.8 (C-7), 39.3 (C-1, 8), 37.8 (C-10), 37.7 (C-3), 37.0 (C-12), 28.7 (C-19), 28.6 (C-22), 21.7 (C-6), 20.2 (C-11), 20.2 (C-17), 19.0 (C-2), 13.4 (C-20) ppm; MS (ESI, MeOH/DMSO, 4:1): *m*/*z* (%) 580 (70%, [M + H]^+^); analysis calcd. for C_38_H_47_N_2_O_3_Br (579.8): C 78.72, H 8.17, N 4.83; found: C 78.56, H 8.37, N 4.65.

#### 4.2.25. 4-Bromobutyl (4α) -13-Hydroxykaur-16-en-18-oate Bromide (**22**)

Following GPB from **1** (0.5 g, 1.57 mmol), K_2_CO_3_ 0.434 g, 3.1 mmol), 1,4-dibromobutane (0.74 mL, 6.3 mmol) followed by chromatography (SiO_2_, hexanes/ethyl acetate, 6:1), **22** (0.53 g, 74%) was obtained as a colorless solid: *R*_f_ = 0.37 (SiO_2_, hexanes/ethyl acetate, 8:2); m.p. = 92–96 °C; ${\left[\alpha \right]}_{D}^{20}$ = −50.12° (*c* = 0.353, CHCl_3_); IR (ATR): ν = 3419*br*, 2934*m*, 2848*m,* 1719*s*, 1465*w*, 1444*m*, 1387*w*, 1365*w*, 1329*m*, 1270*w*, 1229*m*, 1202*w*, 1151*s*, 1118*m*, 1081*m*, 1047*w*, 1020*w*, 973*w*, 953*w*, 920*w*, 882*w*, 868*w*, 818*w*, 754*m*, 694*w*, 647*w*, 562*w*, 531*w*, 501*w*, 432*w* cm^−1^; ^1^H NMR (500 MHz, CDCl_3_): δ = 5.09 (*s*, 1H, OH), 4.96 (*s*, 1H, 17-H_a_), 4.80 (*s*, 1H, 17-H_b_), 4.13–3.98 (*m*, 2H, 21-H), 3.43 (*t*, *J* = 6.6 Hz, 2H, 24-H), 2.19–2.11 (*m*, 3H, 3-H_a_, 14-H_a_, 15-H_a_), 2.00–1.91 (*m*, 2H, 22-H), 1.87–1.72 (*m*, 5H, 1-H_a_, 2-H_a_, 6-H_a_, 23-H), 1.71–1.56 (*m*, 3H, 11-H, 12-H_a_), 1.56–1.34 (*m*, 4H, 2-H_b_, 6-H_b_, 12-H_b_, 15-H_b_), 1.30–1.22 (*m*, 1H, 14-H_b_), 1.16 (*s*, 3H, 19-H), 1.07–0.93 (*m*, 3H, 3-H_b_, 5-H, 9-H), 0.84 (*s*, 3H, 20-H), 0.82–0.78 (*m*, 1H, 1-H_b_) ppm; ^13^C NMR (126 MHz, CDCl_3_): δ = 177.4 (C-18), 143.5 (C-16), 102.9 (C-17), 82.6 (C-13), 63.1 (C-21), 56.7 (C-5), 53.8 (C-9), 50.9 (C-15), 47.9 (C-14), 43.9 (C-4), 41.3 (C-8), 40.9 (C-7), 40.7 (C-1), 39.6 (C-10), 39.4 (C-12), 38.1 (C-3), 31.8 (C-24), 29.6 (C-22), 28.8 (C-19), 27.2 (C-23), 21.0 (C-6), 20.8 (C-11), 19.1 (C-2), 15.3 (C-20) ppm; MS (ESI, MeOH:CHCl_3_ 4:1): *m*/*z* (%) 373 (70%, [M − Br]^+^); analysis calcd. for C_24_H_37_O_3_Br (453.46): C 63.57, H 8.22; found: C 63.41, H 8.39.

#### 4.2.26. 4-Bromobutyl (4α, 8β, 13β) 13-Methyl-16-oxo-17-norkauran-18-oate (**23**)

Following GPB from **2** (0.5 g, 1.57 mmol), K_2_CO_3_ (0.434 g, 3.1 mmol) and 1,4-dibromobutane (0.74 mL, 6.3 mmol) followed by chromatography (SiO_2_, hexanes/ethyl acetate, 6:1), **23** (0.53 g, 75%) was obtained as a colorless solid: *R*_f_ = 0.68 (SiO_2_, hexanes/ethyl acetate, 4:1); m.p. = 104–106 °C; ${\left[\alpha \right]}_{D}^{20}$ = −54.38° (*c* = 0.158, CHCl_3_); IR (ATR): ν = 2937*m*, 2891*w,* 2845*w*, 1734*s*, 1718*s*, 1448*m*, 1387*w*, 1355*w*, 1320*w*, 1299*w*, 1245*w*, 1231*m*, 1208*w*, 1180*s*, 1153*s*, 1133*w*, 1109*w*, 1095*w*, 1060*w*, 1031*w*, 1017*w*, 1002*w*, 978*w*, 928*w*, 868*w*, 850*w*, 827*w*, 809*w*, 776*w*, 750*w*, 735*w*, 694*w*, 652*w*, 594*w*, 557*w*, 513*w*, 462*w* cm^−1^; ^1^H NMR (400 MHz, CDCl_3_): δ = 4.13–3.96 (*m*, 2H, 21-H), 3.43 (*t*, *J* = 6.6 Hz, 2H, 24-H), 2.61 (*dd*, *J* = 18.6, 3.8 Hz, 1H, 15-H_a_), 2.17 (*dt*, *J* = 13.4, 3.8 Hz, 1H, 3-H_a_), 1.99–1.85 (*m*, 3H, 6-H_a_, 22-H), 1.85–1.74 (*m*, 4H, 2-H_a_, 15-H_b_, 23-H), 1.74–1.57 (*m*, 5H, 1-H_a_, 6-H_b_, 7-H_a_, 11-H_a_), 1.53 (*td*, *J* = 11.4, 3.6 Hz, 1H, 14-H_a_), 1.48–1.31 (*m*, 4H, 2-H_b_, 7-H_b_, 12-H, 14-H_b_), 1.18 (*s*, 3H, 19-H), 1.15 (*s*, 2H, 9-H, 11-H_b_), 1.12 (*dd*, *J* = 11.9, 2.2 Hz, 1H, 5-H), 1.03 (*td*, *J* = 13.4, 4.1 Hz, 1H, 3-H_b_), 0.97 (*s*, 3H, 17-H), 0.90 (*td*, *J* = 13.1, 4.2 Hz, 1H, 1-H_b_), 0.70 (*s*, 3H, 20-H) ppm; ^13^C NMR (101 MHz, CDCl_3_): δ = 222.4 (C-16), 177.3 (C-18), 63.2 (C-21), 57.0 (C-5), 54.7 (C-9), 54.3 (C-14), 48.4 (C-15), 41.5 (C-7), 39.8 (C-8), 39.4 (C-1), 38.0 (C-10), 37.9 (C-3), 37.3 (C-12), 32.9 (C-24), 29.5 (C-22), 29.0 (C-19), 27.2 (C-23), 21.8 (C-6), 20.3 (C-11), 19.8 (C-17), 19.0 (C-2), 13.4 (C-20) ppm; MS (ESI, MeOH/CHCl_3_, 4:1): *m*/*z* (%) 373 (90%, [M − Br]^+^); analysis calcd. for C_24_H_37_O_3_Br (453.46): C 63.57, H 8.22; found: C 63.41, H 8.39.

#### 4.2.27. 4-{4-[(E)-2-(1H-Indol-3-yl)ethenyl-pyridinium-1-yl-butyl (4α)-13-Hydroxykaur-16-en-18-oate Bromide (**24**) 

Following GPC from **22** (0.3 g, 0.66 mmol) and **3** (0.146 g, 0.66 mmol) followed by chromatography (SiO_2_, CHCl_3_/MeOH, 6:1), **24** (0.287 g, 73%) was obtained as a reddish solid: *R*_f_ = 0.11 (SiO_2_, CHCl_3_/MeOH, 9:1); m.p. = 179–182 °C; ${\left[\alpha \right]}_{D}^{20}$ = −44.96° (*c* = 0.045, MeOH); IR (ATR): ν = 3308*br*, 2953*w,* 2934*m*, 2857*w*, 2843*w*, 1716*m*, 1647*w*, 1591*m*, 1573*m*, 1556*w*, 1492*m*, 1460*w*, 1439*m*, 1385*w*, 1371*w*, 1356*w*, 1316*w*, 1278*w*, 1245*m*, 1207*w*, 1179*m*, 1150*w*, 1136*w*, 1116*m*, 1083*w*, 1062*w*, 1047*w*, 1021*w*, 984*w*, 967*w*, 940*w*, 899*w*, 872*w*, 841*w*, 820*w*, 802*w*, 766*w*, 751*m*, 696*w*, 617*w*, 574*w*, 551*w*, 527*w*, 513*w*, 499*w*, 429*w* cm^−1^; UV-Vis (MeOH): λ_max_ (log ε) = 438.12 nm (4.14); ^1^H NMR (500 MHz, DMSO-d_6_): δ = 11.97 (*s*, 1H, NH), 8.78 (*d*, *J* = 7.0 Hz, 2H, 25-H, 29-H), 8.27 (*d*, *J* = 16.1 Hz, 1H, 31-H), 8.15 (*m*, 3H, 26-H, 28-H, 34-H), 7.98 (*d*, *J* = 2.7 Hz, 1H, 39-H), 7.51 (*d*, *J* = 7.3 Hz, 1H, 37-H), 7.30 (*d*, *J* = 16.2 Hz, 3H, 30-H), 7.27–7.19 (*m*, 2H, 35-H, 36-H), 5.00 (*s*, 1H, 17-H_a_), 4.64 (*s*, 1H, 17-H_b_), 4.48 (*t*, *J* = 7.1 Hz, 2H, 24-H), 4.12–3.94 (*m*, 2H, 21-H), 2.07–1.90 (*m*, 6H, 3-H_a_, 14-H, 15-H_a_, 22-H), 1.78–1.66 (*m*, 3H, 1-H_a_, 2-H_a_, 6-H_a_), 1.65–1.48 (*m*, 4H, 11-H, 23-H), 1.48–1.40 (*m*, 2H, 12-H), 1.39–1.18 (*m*, 3H, 2-H_b_, 6-H_b_, 15-H_b_), 1.10 (*s*, 3H, 19-H), 1.06–0.87 (*m*, 3H, 3-H_b_, 5-H, 9-H), 0.82–0.76 (*m*, 1H, 1-H_b_), 0.74 (*s*, 3H, 20-H) ppm; ^13^C NMR (126 MHz, DMSO-d_6_): δ = 176.4 (C-18), 154.7 (C-16), 144.5 (C-27), 143.3 (C-25, 29), 137.5 (C-38), 136.7 (C-31), 133.2 (C-39), 132.4, 124.9 (C-33), 122.9 (C-35), 121.9 (C-26, 28), 121.1 (C-36), 120.4 (C-34), 116.8 (C-30), 113.6 (C-32), 112.6 (C-37), 102.5 (C-17), 80.7 (C-13), 62.9 (C-21), 58.5 (C-24), 55.7 (C-5), 53.1 (C-9), 50.5 (C-15), 47.2 (C-14), 43.3 (C-4), 43.2 (C-8), 41.0 (C-7), 40.2 (C-1), 39.0 (C-12), 38.8 (C-10), 37.4 (C-3), 28.3 (C-19), 27.4 (C-22), 24.8 (C-23), 20.5 (C-6), 20.4 (C-11), 18.7 (C-2), 15.0 (C-20) ppm; MS (ESI, MeOH/DMSO, 4:1): *m*/*z* (%) 594 (75%, [M − Br]^+^); analysis calcd. for C_39_H_49_N_2_O_3_Br (673.72): C 69.53, H 7.33, N 4.16; found: C 69.39, H 7.54, N 3.87.

#### 4.2.28. 4-{4[(E)-2-(1H-Indol-3-yl)ethenyl]-pyridinium-1-yl}butyl (4α, 8β, 13β) 13-Methyl-16-oxo-17-norkauran-18-oate Bromide (**25**)

Following GPC from **23** (0.3 g, 0.66 mmol) and **3** (0.146 g, 0.66 mmol) followed by chromatography (SiO_2_, CHCl_3_/MeOH, 6:1), **25** (0.314 g, 80%) was obtained as a reddish solid: *R*_f_ = 0.17 (SiO_2_, CHCl_3_/MeOH, 9:1); m.p. = 174–176 °C; ${\left[\alpha \right]}_{D}^{20}$ = −7.30° (*c* = 0.178, MeOH); IR (ATR): ν = 3406*br*, 3128*br*, 2925*m*, 2848*w,* 1721*m*, 1645*w*, 1597*s*, 1575*m*, 1557*w*, 1531*w*, 1501*m*, 1457*m*, 1432*m*, 1371*w*, 1356*w*, 1318*w*, 1274*w*, 1246*m*, 1206*m*, 1173*s*, 1132*s*, 1112*m*, 1097*w*, 1058*w*, 1044*w*, 1016*w*, 965*m*, 928*w*, 871*w*, 744*s*, 663*w*, 612*w*, 590*w*, 569*w*, 510*w*, 425*w* cm^−1^; UV-Vis (MeOH): λ_max_ (log ε) = 443.61 nm (4.58); ^1^H NMR (500 MHz, DMSO-d_6_): δ = 11.97 (*s*, 1H, NH), 8.79 (*d*, *J* = 6.9 Hz, 2H, 25-H, 29-H), 8.26 (*d*, *J* = 16.3 Hz, 1H, 31-H), 8.17–8.12 (*m*, 3H, 26-H, 28-H, 34-H), 7.96 (*d*, *J* = 2.4 Hz, 1H, 39-H), 7.51 (*d*, *J* = 7.3 Hz, 1H, 37-H), 7.29 (*d*, *J* = 16.2 Hz, 1H, 30-H), 7.27–7.20 (*m*, 2H, 35-H, 36-H), 4.47 (*t*, *J* = 6.1 Hz, 2H, 21-H), 4.06–3.93 (*m*, 2H, 24-H), 2.35 (*dd*, *J* = 18.3, 3.5 Hz, 1H, 15-H_a_), 2.02 (*d*, *J* = 13.0 Hz, 1H, 3-H_a_), 1.99–1.88 (*m*, 2H, 23-H), 1.80 (*d*, *J* = 18.3 Hz, 1H, 15-H_b_), 1.79–1.70 (*m*, 1H, 6-H_b_), 1.71–1.51 (*m*, 6H, 1-H_a_,2-H_a_, 7-H_a_, 11-H_a_, 22-H), 1.49 (*d*, *J* = 14.0 Hz, 1H, 6-H_a_), 1.44 (*dd*, *J* = 11.3, 1.9 Hz, 1H, 14-H_a_), 1.39 (*td*, *J* = 13.1, 3.1 Hz, 1H, 7-H_b_), 1.39–1.18 (*m*, 4H, 2-H, 12-H, 14-H_b_), 1.19–1.13 (*m*, 2H, 5-H, 9-H), 1.12 (*s*, 3H, 19-H), 1.08–0.94 (*m*, 2H, 3-H_b_, 11-H_b_), 0.87 (*td*, *J* = 13.4, 13.0, 4.2 Hz, 1H, 1-H_b_), 0.78 (*s*, 3H, 17-H), 0.57 (*s*, 3H, 20-H) ppm; ^13^C NMR (126 MHz, DMSO-d_6_): δ = 221.1 (C-16), 176.9 (C-18), 155.2 (C-27), 143.8 (C-25, 29), 138.0 (C-38), 137.2 (C-31), 133.0 (C-39), 125.4 (C-33), 123.4 (C-36), 122.4 (C-26, 28), 121.6 (C-35), 120.9 (C-34), 117.2 (C-30), 114.1 (C-32), 113.1 (C-37), 63.5 (C-24), 59.0 (C-21), 56.4 (C-5), 54.1 (C-9), 53.6 (C-14), 48.3 (C-13), 48.0 (C-15), 43.7 (C-4), 41.0 (C-7), 39.5 (C-1), 39.4 (C-8), 37.9 (C-10), 37.8 (C-3), 37.0 (C-12), 28.9 (C-19), 28.0 (C-23), 25.2 (C-22), 21.8 (C-6), 20.3 (C-11), 20.1 (C-17), 19.0 (C-2), 13.5 (C-20) ppm; MS (ESI, MeOH/DMSO, 4:1): *m*/*z* (%) 594 (80%, [M − Br]^+^); analysis calcd. for C_39_H_49_N_2_O_3_Br (673.72): C 69.53, H 7.33, N 4.16; found: C 69.40, H 7.53, N 3.89.

#### 4.2.29. 4-{3-[(E)-2-(1H-Indol-3-yl)-ethenyl]-pyridinium-1-yl}butyl (4α)-13-Hydroxykaur-16-en-18-oate Bromide (**26**)

Following GPC from **22** (0.526 g, 1.15 mmol) and **4** (0.256 g, 1.16 mmol) followed by chromatography (SiO_2_, CHCl_3_/MeOH, 6:1), **26** (0.420 g, 61%) was obtained as a yellowish solid: *R*_f_ = 0.06 (SiO_2_, CHCl_3_/MeOH, 9:1); m.p. = 182–184 °C; ${\left[\alpha \right]}_{D}^{20}$ = −33.43° (*c* = 0.157, MeOH); IR (ATR): ν = 3376*br*, 3210*br*, 2933*w*, 2849*w,* 1707*m*, 1632*m*, 1577*m*, 1525*w*, 1501*w*, 1459*w*, 1434*m*, 1386*w*, 1364*w*, 1329*w*, 1276*w*, 1236*m*, 1202*w*, 1152*m*, 1116*m*, 1082*w*, 1055*w*, 1018*w*, 954*w*, 819*w*, 745*s*, 680*w*, 618*w*, 570*w*, 500*w*, 424*w* cm^−1^; UV-Vis (MeOH): λ_max_ (log ε) = 359.12 nm (4.17); ^1^H NMR (500 MHz, DMSO-d_6_): δ = 11.65 (*s*, 1H, NH), 9.36 (*s*, 1H, 25-H), 8.82 (*d*, *J* = 5.9 Hz, 1H, 29-H), 8.74 (*d*, *J* = 8.4 Hz, 1H, 27-H), 8.32 (*s*, 1H), 8.11–8.05 (*m*, 2H, 28-H, 34-H), 7.90 (*d*, *J* = 16.5 Hz, 1H, 31-H), 7.78 (*d*, *J* = 2.7 Hz, 1H, 39-H), 7.49 (*d*, *J* = 7.7 Hz, 2H, 37-H), 7.22 (*d*, *J* = 16.4 Hz, 1H, 30-H), 7.25–7.14 (*m*, 2H, 35-H, 36-H), 4.99 (*s*, 1H, 17-H_a_), 4.73–4.55 (*m*, 3H, 17-H_b_, 24-H), 4.10–3.95 (*m*, 2H, 21-H), 2.11–1.97 (*m*, 5H, 3-H_a_, 14-H, 23-H), 1.90 (*d*, *J* = 9.3 Hz, 1H, 15-H_a_), 1.82–1.57 (*m*, 6H, 1-H_a_, 2-H_a_, 6-H_a_, 11-H_a_, 22-H), 1.56–1.40 (*m*, 3H, 11-H_b_, 12-H), 1.39–1.15 (*m*, 3H, 2-H_b_, 6-H_b_, 15-H_b_), 1.10 (*s*, 3H, 19-H), 1.02 (*dd*, *J* = 12.1, 1.6 Hz, 1H, 5-H), 0.95 (*td*, *J* = 13.5, 9.3, 3.7 Hz, 1H, 3-H_b_), 0.89 (*d*, *J* = 8.0 Hz, 1H, 9-H), 0.77 (*d*, *J* = 8.5 Hz, 1H, 1-H_b_), 0.74 (*s*, 3H, 20-H) ppm; ^13^C NMR (126 MHz, DMSO-d_6_): δ = 176.4 (C-18), 156.2 (C-16), 144.5 (C-38), 141.3 (C-25), 140.5 (C-29), 139.5 (C-27), 137.3 (C-26), 133.2, 130.2 (C-31), 129.1 (C-39), 127.7 (C-28), 124.8 (C-33), 122.4 (C-35), 120.4 (C-36), 120.0 (C-34), 115.3 (C-30), 112.9 (C-32), 112.3 (C-37), 102.5 (C-17), 80.7 (C-13), 62.9 (C-24), 60.3 (C-21), 55.7 (C-5), 53.0 (C-9), 50.5 (C-15), 47.2 (C-14), 43.2 (C-4), 41.5 (C-8), 41.0 (C-7), 40.8 (C-1), 40.2 (C-10), 38.7 (C-12), 37.4 (C-3), 31.8, 28.3 (C-19), 27.5 (C-23), 24.8 (C-22), 20.5 (C-6), 20.4 (C-11), 18.7 (C-2), 15.0 (C-20) ppm; MS (ESI, MeOH/DMSO, 4:1): *m*/*z* (%) 594 (70%, [M − Br]^+^); analysis calcd. For C_39_H_49_N_2_O_3_Br (673.72): C 69.53, H 7.33, N 4.16; found: C 69.40, H 7.52, N 3.97.

#### 4.2.30. 4-{3-[(E)-2-(1H-Indol-3-yl)ethenyl]-pyridinium-1-yl}butyl (4α, 8β, 13β) 13-Methyl-16-oxo-17-norkauran-18-oate Bromide (**27**)

Following GPC from **23** (0.533 g, 1.17 mmol) and **4** (0.26 g, 1.18 mmol) followed by chromatography (SiO_2_, CHCl_3_/MeOH 6:1), **27** (0.447 g, 65%) was obtained as a yellowish solid: *R*_f_ = 0.09 (SiO_2_, CHCl_3_/MeOH 9:1); m.p. = 167–169 °C; ${\left[\alpha \right]}_{D}^{20}$ = −28.15° (*c* = 0.115, MeOH); IR (ATR): ν = 3417*br*, 4198*br*, 2928*w*, 2848*w,* 1722*m*, 1633*m*, 1578*m*, 1525*w*, 1501*m*, 1456*w*, 1434*m*, 1372*w*, 1337*w*, 1321*w*, 1276*w*, 1250*w*, 1233*m*, 1179*w*, 1151*w*, 1132*w*, 1111*w*, 1059*w*, 1029*w*, 1016*w*, 959*w*, 929*w*, 824*w*, 744*m*, 681*w*, 618*w*, 598*w*, 569*w*, 504*w*, 424*w* cm^−1^; UV-Vis (MeOH): λ_max_ (log ε) = 357.9 nm (4.25); ^1^H NMR (500 MHz, DMSO-d_6_): δ = 11.65 (*s*, 1H, NH), 9.37–9.31 (*m*, 1H, 25-H), 8.83 (*d*, *J* = 5.9 Hz, 1H, 29-H), 8.76 (*d*, *J* = 8.4 Hz, 1H, 27-H), 8.15–8.06 (*m*, 2H, 28-H, 34-H), 7.89 (*d*, *J* = 16.5 Hz, 1H, 31-H), 7.78 (*d*, *J* = 2.7 Hz, 1H, 39-H), 7.50 (*d*, *J* = 7.7 Hz, 1H, 37-H), 7.22 (*d*, *J* = 15.7 Hz, 1H, 30-H), 7.27–7.16 (*m*, 2H, 35-H, 36-H), 4.65 (*t*, *J* = 7.1 Hz, 2H, 24-H), 4.11–3.97 (*m*, 2H, 21-H), 2.35 (*d*, *J* = 21.9 Hz, 1H, 15-H_a_), 2.14–1.98 (*m*, 3H, 3-H_a_, 23-H), 1.82 (*d*, *J* = 18.3 Hz, 1H, 15-H_b_), 1.82–1.75 (*m*, 1H, 6-H_a_), 1.73–1.47 (*m*, 7H, 1-H_a_, 2-H_a_, 6-H_b_, 7-H_a_, 11-H_a_, 22-H), 1.43 (*dd*, *J* = 11.4, 2.1 Hz, 1H, 14-H_a_), 1.40–1.21 (*m*, 5H, 2-H_b_, 7-H_b_, 12-H, 14-H_b_), 1.19–1.15 (*m*, 2H, 5-H, 9-H), 1.14 (*s*, 3H, 19-H), 1.02 (*m*, 2H, 3-H_b_, 11-H_b_), 0.89 (*td*, *J* = 13.3, 3.9 Hz, 1H, 1-H_b_), 0.84 (*s*, 3H, 17-H), 0.59 (*s*, 3H, 20-H) ppm; ^13^C NMR (126 MHz, DMSO-d_6_): δ = 221.1 (C-16), 176.9 (C-18), 141.7 (C-25), 141.0 (C-29), 140.0 (C-38), 140.0 (C-27), 137.8 (C-26), 130.7 (C-31), 129.7 (C-39), 128.2 (C-28), 125.3 (C-33), 122.9 (C-30), 120.9 (C-36), 120.4 (C-34), 115.8 (C-35), 113.4 (C-32), 112.8 (C-37), 63.5 (C-24), 60.9 (C-21), 56.4 (C-5), 54.1 (C-9), 53.5 (C-14), 48.3 (C-13), 48.0 (C-15), 43.7 (C-4), 41.0 (C-7), 39.4 (C-1, 8), 37.9 (C-10), 37.8 (C-3), 37.0 (C-12), 28.9 (C-19), 28.0 (C-23), 25.2 (C-22), 21.8 (C-6), 20.2 (C-11), 20.1 (C-17), 19.0 (C-2), 13.5 (C-20) ppm; MS (ESI, MeOH/DMSO, 4:1): *m*/*z* (%) 594 (80%, [M + H]^+^); analysis calcd. for C_39_H_49_N_2_O_3_Br (673.72): C 69.53, H 7.33, N 4.16; found: C 69.46, H 7.58, N 3.86.

#### 4.2.31. 4-{2-[(E{-2-(1H-Indol-3-yl)ethenyl]pyridinium-1-yl}-butyl (4α)-13-Hydroxykaur-16-en-18-oate Bromide (**28**) 

Following GPC from **22** (0.263 g, 0.58 mmol) and **5** (0.128 g, 0.58 mmol) followed by chromatography (SiO_2_, CHCl_3_/MeOH, 4:1), **28** (0.123 g, 36%) was obtained as a yellowish solid: *R*_f_ = 0.19 (SiO_2_, CHCl_3_/MeOH 8.5:1.5); m.p. = 84–87 °C; ${\left[\alpha \right]}_{D}^{20}$ = −21.46° (*c* = 0.135, MeOH); IR (ATR): ν = 3375*br*, 2925*w*, 2851*w,* 1715*w*, 1627*w*, 1605*m*, 1563*m*, 1499*w*, 1472*w*, 1432*m*, 1364*w*, 1329*w*, 1319*w*, 1278*w*, 1242*m*, 1153*m*, 1135*w*, 1117*w*, 1082*w*, 1055*w*, 1019*w*, 955*w*, 864*w*, 815*w*, 743*w*, 694*w*, 610*w*, 567*w*, 515*w*, 422*w* cm^−1^; UV-Vis (MeOH): λ_max_ (log ε) = 426.84 nm (4.14); ^1^H NMR (400 MHz, DMSO-d_6_): δ = 12.11 (*s*, 1H, NH), 8.81 (*d*, *J* = 5.8 Hz, 1H, 25-H), 8.57 (*d*, *J* = 8.2 Hz, 1H, 28-H), 8.37 (*d*, *J* = 8.1 Hz, 1H, 26-H), 8.32 (*d*, *J* = 15.4 Hz, 1H, 31-H), 8.19 (*s*, 1H, 39-H), 8.06 (*d*, *J* = 7.2 Hz, 1H, 34-H), 7.72 (*t*, 1H, 27-H), 7.50 (*d*, *J* = 7.1 Hz, 1H, 37-H), 7.28 (*d*, *J* = 16.1 Hz, 1H, 30-H), 7.22 (*s*, 2H, 35-H, 36-H), 4.94 (*s*, 1H, 17-H_a_), 4.90–4.74 (*m*, 2H, 24-H), 4.66 (*s*, 1H, 17-H_b_), 4.12–3.87 (*m*, 2H, 21-H), 2.03–1.83 (*m*, 5H, 3-H_a_, 14-H, 23-H), 1.75 (*d*, 1H, 15-H_a_), 1.73–1.36 (*m*, 10H, 1-H_a_, 2-H_a_, 6-H_a_, 7-H_a_, 11-H, 12-H, 22-H), 1.26 (*m*, 4H, 2-H_b_, 6-H_b_, 7-H_b_, 15-H_b_), 0.92 (*s*, 3H, 19-H), 0.90–0.74 (*m*, 3H, 3-H_b_, 5-H, 9-H), 0.72–0.64 (*m*, 1H, 1-H_b_), 0.62 (*s*, 3H, 20-H) ppm; ^13^C NMR (101 MHz, DMSO-d_6_): δ = 176.8 (C-18), 156.7 (C-16), 153.5 (C-29), 145.1 (C-25), 144.9 (C-38), 143.7 (C-26), 139.0 (C-31), 137.8 (C-33), 132.8 (C-39), 125.6 (C-32), 124.4 (C-28), 123.4 (C-27), 123.3 (C-36), 121.7 (C-35), 120.3 (C-34), 113.9 (C-17), 113.1 (C-37), 110.0 (C-30), 81.2 (C-13), 63.4 (C-21), 61.5 (C-24), 56.1 (C-5), 53.5 (C-9), 51.0 (C-15), 47.6 (C-14), 43.6 (C-4), 42.3 (C-8), 41.7 (C-7), 40.5 (C-1), 40.1 (C-10), 39.1 (C-12), 37.8 (C-3), 28.6 (C-19), 26.6 (C-23), 25.4 (C-22), 21.0 (C-6), 20.8 (C-11), 19.1 (C-2), 15.4 (C-20) ppm; MS (ESI, MeOH/DMSO, 4:1): *m*/*z* (%) 594 (80%, [M − Br]^+^); analysis calcd. for C_39_H_49_N_2_O_3_Br (673.72): C 69.53, H 7.33, N 4.16; found: C 69.42, H 7.57, N 3.91.

#### 4.2.32. 4-{2-[(E)-2-(1H-Indol-3-yl)ethenyl]pyridinium-1-yl}-butyl (4α, 8β, 13β) 13-Methyl-16-oxo-17-norkauran-18-oate Bromide (**29**)

Following GPC from **23** (0.267 g, 0.59 mmol) and **5** (0.13 g, 0.59 mmol) followed by chromatography (SiO_2_, CHCl_3_/MeOH, 4:1), **29** (1.04 g, 30%) was obtained as a yellowish solid: *R*_f_ = 0.35 (SiO_2_, CHCl_3_/MeOH, 8.5:1.5); m.p. = 86–89 °C; ${\left[\alpha \right]}_{D}^{20}$ = −5.25° (*c* = 0.294, MeOH); IR (ATR): ν = 3381*br*, 2924*w*, 2850*w,* 1719*m*, 1660*w*, 1627*w*, 1605*m*, 1562*m*, 1523*w*, 1499*w*, 1469*w*, 1432*m*, 1380*w*, 1335*w*, 1319*w*, 1278*w*, 1242*m*, 1156*w*, 1131*w*, 1112*w*, 1059*w*, 1016*w*, 966*w*, 855*w*, 816*w*, 744*m*, 661*w*, 564*w*, 506*w*, 459*w*, 423*w* cm^−1^; UV-Vis (MeOH): λ_max_ (log ε) = 427.68 nm (4.67); ^1^H NMR (500 MHz, DMSO-d_6_): δ = 12.10 (*s*, 1H, NH), 8.81 (*d*, *J* = 6.2 Hz, 1H, 25-H), 8.58 (*d*, *J* = 8.4 Hz, 1H, 28-H), 8.40–8.32 (*m*, 1H, 26-H), 8.33 (*d*, *J* = 15.9 Hz, 1H, 31-H), 8.20 (*d*, *J* = 2.8 Hz, 1H, 39-H), 8.07 (*d*, *J* = 7.0 Hz, 1H, 34-H), 7.73 (*t*, *J* = 6.9 Hz, 1H, 27-H), 7.50 (*d*, *J* = 7.7 Hz, 1H, 37-H), 7.30 (*d*, *J* = 15.6 Hz, 1H, 30-H), 7.27–7.17 (*m*, 2H, 35-H, 36-H), 4.83 (*t*, *J* = 7.3 Hz, 2H, 24-H), 4.14–3.86 (*m*, 2H, 21-H), 2.24 (*dd*, *J* = 18.3, 3.3 Hz, 1H, 15-H_a_), 2.05–1.87 (*m*, 3H, 3-H_a_, 23-H), 1.78–1.51 (*m*, 5H, 2-H_a_, 6-H_a_, 15-H_b_, 22-H), 1.51–1.18 (*m*, 10H, 1-H_a_, 2-H_b_, 6-H_b_, 7-H, 11-H_a_, 12-H, 14-H), 1.08–0.98 (*m*, 2H, 5-H, 9-H), 0.96 (*s*, 3H, 19-H), 0.94–0.86 (*m*, 2H, 3-H_b_, 11-H_b_), 0.86–0.80 (*m*, 3H, 17-H), 0.75 (*td*, *J* = 12.9, 12.4, 4.9 Hz, 1H, 1-H_b_), 0.48 (*s*, 3H, 20-H) ppm; ^13^C NMR (126 MHz, DMSO-d_6_): δ = 221.0 (C-16), 176.8, 153.5 (C-29), 145.1 (C-25), 143.7 (C-26), 139.0 (C-31), 137.8 (C-38), 132.7 (C-39), 129.8, 125.7 (C-33), 124.4 (C-28), 123.4 (C-27), 122.7 (C-36), 121.7 (C-35), 120.3 (C-34), 113.9 (C-32), 113.1 (C-37), 110.0 (C-30), 63.6 (C-21), 57.2 (C-24), 56.3 (C-5), 54.0 (C-9), 53.5 (C-14), 48.3 (C-13), 47.9 (C-15), 43.6 (C-4), 40.8 (C-7), 39.4 (C-1), 39.4 (C-8), 39.3 (C-10), 37.8 (C-3), 37.0 (C-12), 28.7 (C-19), 26.8 (C-23), 25.4 (C-22), 21.7 (C-6), 20.2 (C-17), 20.2 (C-11), 18.9 (C-2), 13.4 (C-20) ppm; MS (ESI, MeOH/DMSO, 4:1): *m*/*z* (%) 594 (75%, [M + H]^+^); analysis calcd. For C_39_H_49_N_2_O_3_Br (673.72): C 69.53, H 7.33, N 4.16; found: C 69.36, H 7.59, N 3.94.

## Figures and Tables

**Table 1:** Cytotoxicity (IC_50_ in μM) of isosteviol derivatives from SRB assays; cell lines: A2780 (ovarian carcinoma), A2780cis (resistant derivative of A2780), A549 (lung carcinoma), MCF7 (breast carcinoma), HT29 (colorectal carcinoma), CCD18Co (non-malignant human fibroblasts); results are mean values (n = 3) with SD for all experiments between 5 and 10%. **DOX**: doxorubicin as a positive standard; SL denotes the spacer length, SP the substitution pattern (*ortho*, *meta* or *para* in the pyridinium moiety); resistance index (RI): quotient IC_50_ values A2780cis/A2780; selectivity index 1 (SI 1): CCD18Co/A2780, SI 2 = CCD18Co/A549.

SL	SP	#	A2780	A2780cis	A549	MCF7	HT29	CCD18Co	RI	SI 1	SI 2
	o	**3**	1.19	39.23	26.27	1.12	28.70	87.41	32.88	73.27	3.33
	m	**4**	1.17	20.97	16.41	5.18	7.83	90.32	17.97	77.40	5.50
	p	**5**	2.26	16.42	15.41	10.86	16.49	100.00	7.26	44.21	6.49
2	Br	**7**	19.12	28.70	32.07	11.84	16.14	62.28	1.50	3.26	1.94
2	o	**13**	1.92	4.07	10.85	3.83	6.35	20.64	2.12	10.75	1.90
2	m	**11**	2.94	5.24	23.59	8.73	9.42	29.35	1.78	9.97	1.24
2	p	**9**	0.93	1.54	9.09	1.08	1.56	12.95	1.66	13.96	1.42
3	Br	**15**	9.00	16.65	24.77	12.78	15.13	41.48	1.85	4.61	1.67
3	o	**21**	1.86	2.35	9.27	1.71	3.38	10.06	1.26	5.42	1.09
3	m	**19**	0.99	1.93	11.22	2.50	5.05	15.49	1.94	15.60	1.38
3	p	**17**	0.28	0.30	3.39	0.59	0.76	19.93	1.07	71.25	5.88
4	Br	**23**	13.03	29.10	34.29	18.36	24.61	31.33	2.23	2.40	0.91
4	o	**29**	1.06	3.22	9.86	2.05	3.81	18.58	3.05	17.61	1.88
4	m	**27**	0.76	1.27	6.28	1.57	2.20	10.83	1.67	14.18	1.72
4	p	**25**	0.18	0.31	2.62	0.49	0.88	10.85	1.70	59.32	4.14
		**DOX**	0.007	0.109	0.017	0.083	0.098	0.242	14.7	32.7	13.9

**Table 2:** Cytotoxicity (IC_50_ in μM) of steviol derivatives from SRB assays; cell lines: A2780 (ovarian carcinoma), A2780cis (resistant derivative of A2780), A549 (lung carcinoma), MCF7 (breast carcinoma), HT29 (colorectal carcinoma), CCD18Co (non-malignant human fibroblasts); results are mean values (n = 3) with SD for all experiments between 5 and 10%. **DOX**: doxorubicin as a positive standard; SL denotes the spacer length, SP the substitution pattern (*ortho*, *meta* or *para* in the pyridinium moiety); resistance index (RI): quotient IC_50_ values A2780cis/A2780; selectivity index 1 (SI 1): CCD18Co/A2780, SI 2 = CCD18Co/A549.

SL	SP	#	A2780	A2780cis	A549	MCF7	HT29	CCD18Co	RI	SI 1	SI 2
2	Br	**6**	24.78	28.08	83.38	29.42	30.59	42.19	1.13	1.70	0.51
2	o	**12**	25.73	29.43	54.31	20.23	28.51	75.93	1.14	2.95	1.40
2	m	**10**	2.80	4.20	16.69	3.83	7.65	22.72	1.50	8.12	1.36
2	p	**8**	2.01	1.86	8.82	1.81	1.81	11.82	0.92	5.87	1.34
3	Br	**14**	27.65	28.36	28.97	28.09	29.11	41.72	1.03	1.51	1.44
3	o	**20**	1.81	2.70	12.57	1.85	6.11	19.44	1.49	10.73	1.55
3	m	**18**	2.28	2.71	9.47	9.47	6.66	26.91	1.19	11.82	2.84
3	p	**16**	0.32	0.45	3.00	0.72	0.98	8.33	1.41	26.27	2.77
4	Br	**22**	16.66	18.95	35.62	21.17	29.52	59.68	1.14	3.58	1.68
4	o	**28**	0.50	1.68	7.51	1.18	1.78	10.94	3.33	21.75	1.46
4	m	**26**	1.28	1.82	6.94	1.98	2.43	11.73	1.42	9.14	1.69
4	p	**24**	0.24	0.28	2.76	0.58	0.88	13.09	1.13	53.96	4.74

## Data Availability

Data are contained within the article and the Supplementary Material.

## Supplementary Materials

The following supporting information can be downloaded at: https://www.mdpi.com/article/10.3390/molecules29020381/s1.
