# Supplementary material for: F16 Hybrids Derived from Steviol or Isosteviol Are Accumulated in the Mitochondria of Tumor Cells and Overcome Drug Resistance
Source: Molecules. 2024 Jan 12;29(2):381. doi: 10.3390/molecules29020381 (PMC10821019; doi:10.3390/molecules29020381)

Supplementary Materials

F16 Hybrids Derived from Steviol or Isosteviol are Accumulated in the Mitochondria of A549 Tumor Cells and Overcome Drug Resistance

Niels V. Heise, Julia Heisig, Kristof Meier, René Csuk *, Thomas Mueller

High resolution pictures of cell staining experiments

Subcellular accumulation of compounds 17 and 25 compared to 5 (F16-group) was studied employing the fluorescent characteristics mediated by the F16-group. Cells were co-treated with compounds, the strong mitochondria-targeted and NIR fluorescent agent AHCS2, and Hoechst 33342 for staining of nuclei. Live cell imaging was performed. The different fluorescence spectra enabled simultaneous analysis and direct comparison. Shown are the single images taken in different channels: AHCS2 (deep-red), Hoechst (violet, DAPI), Compounds (green), and the merged versions.

Similar subcellular accumulation pattern of the F16 compounds and AHCS2 could be observed, proving mitochondrial targeting. Thorough analysis, especially of the merged pictures, revealed some minor accumulation of the F16 diterpene hybrids in other cellular areas in addition to mitochondria.

Compound 17_AHCS2

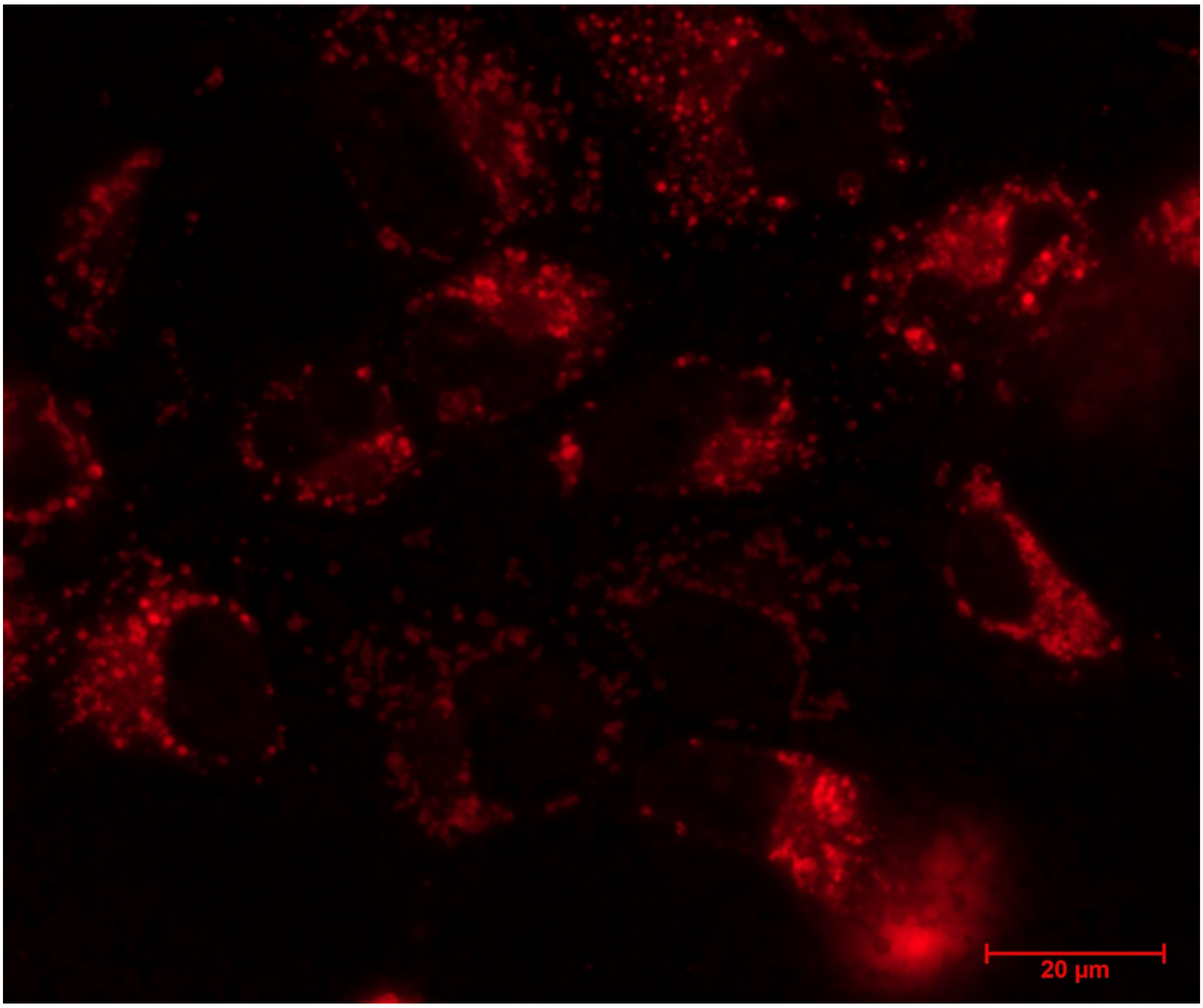

Compound 17_DAPI

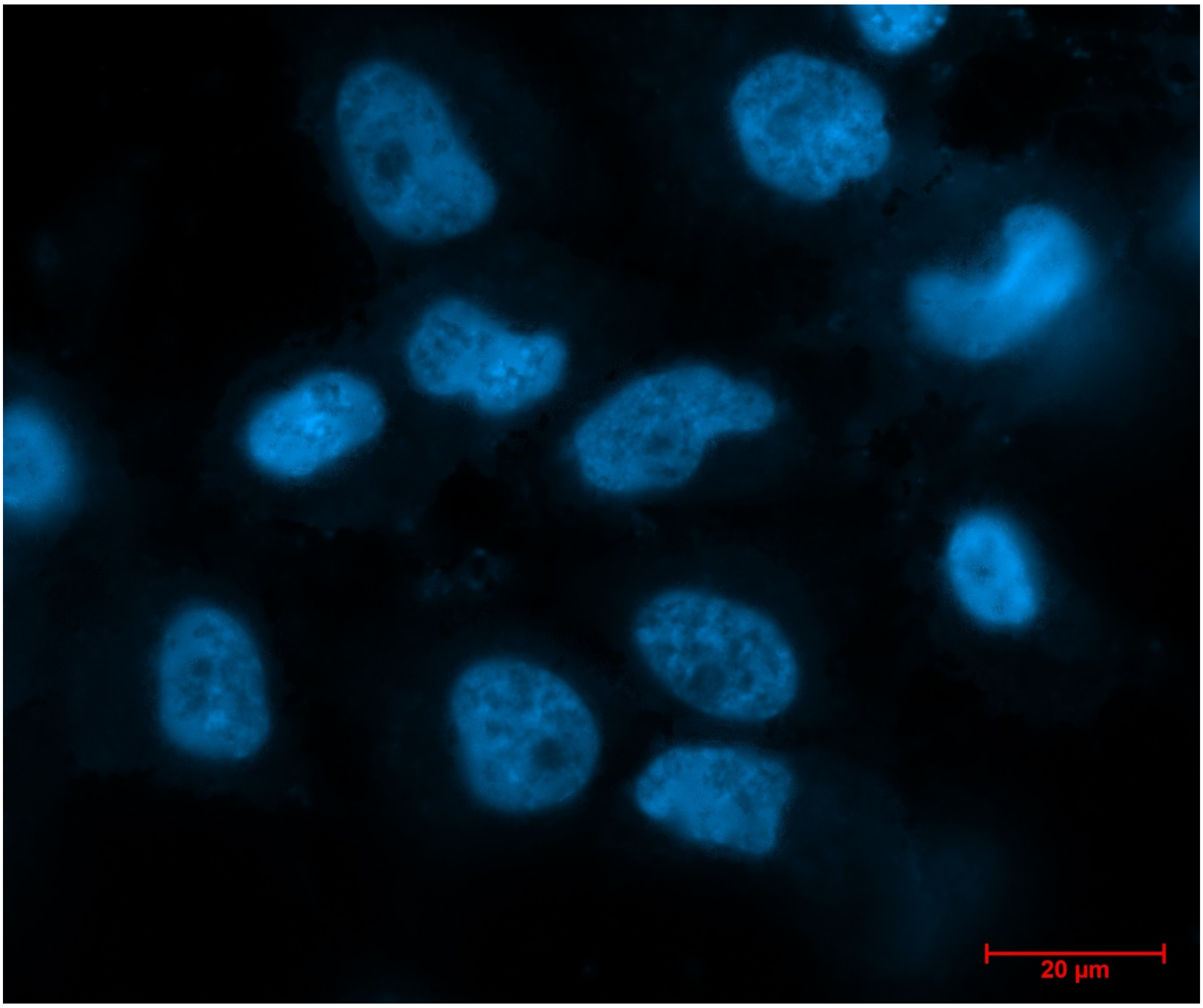

Compound 17_green

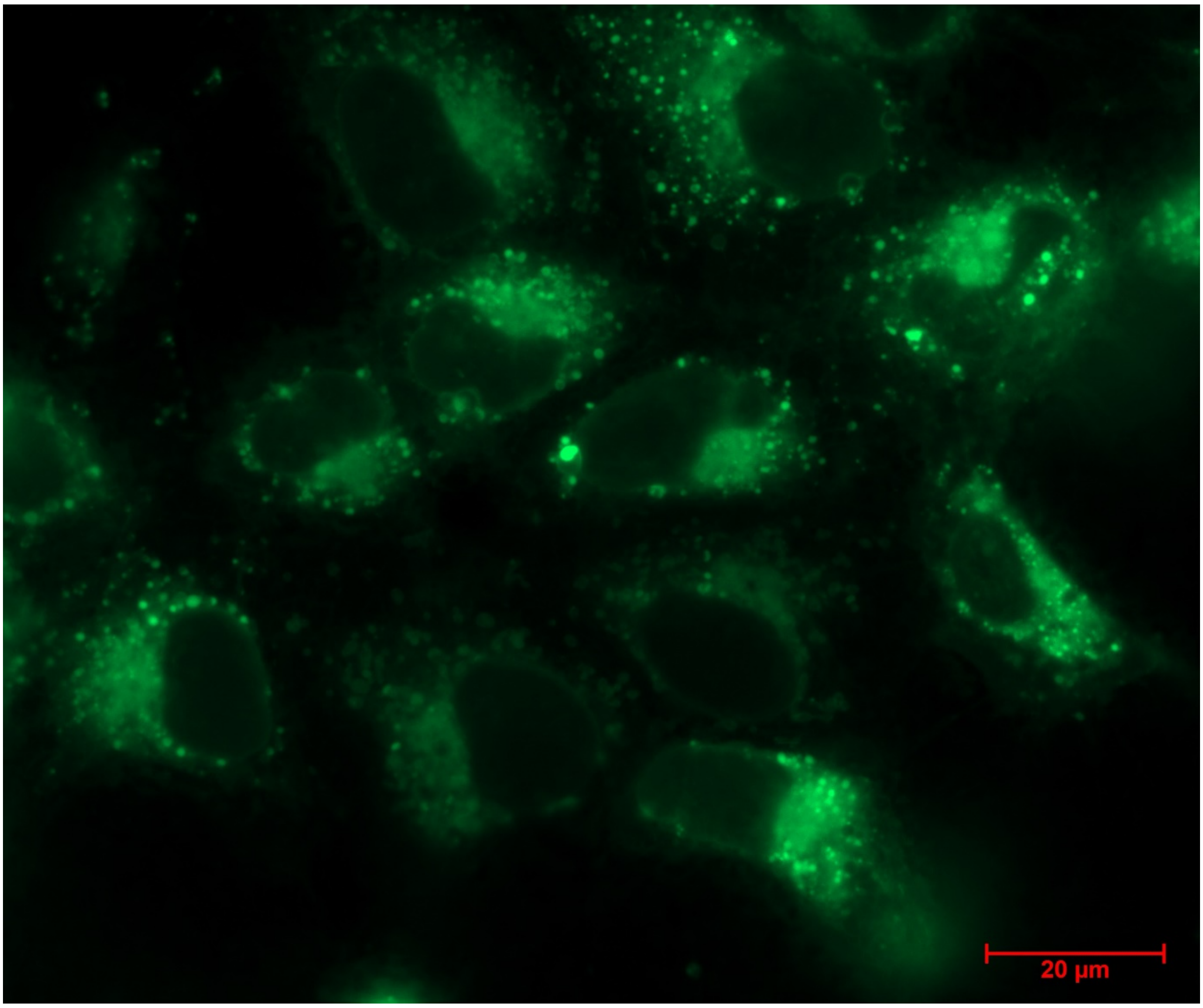

Compound 17_merged

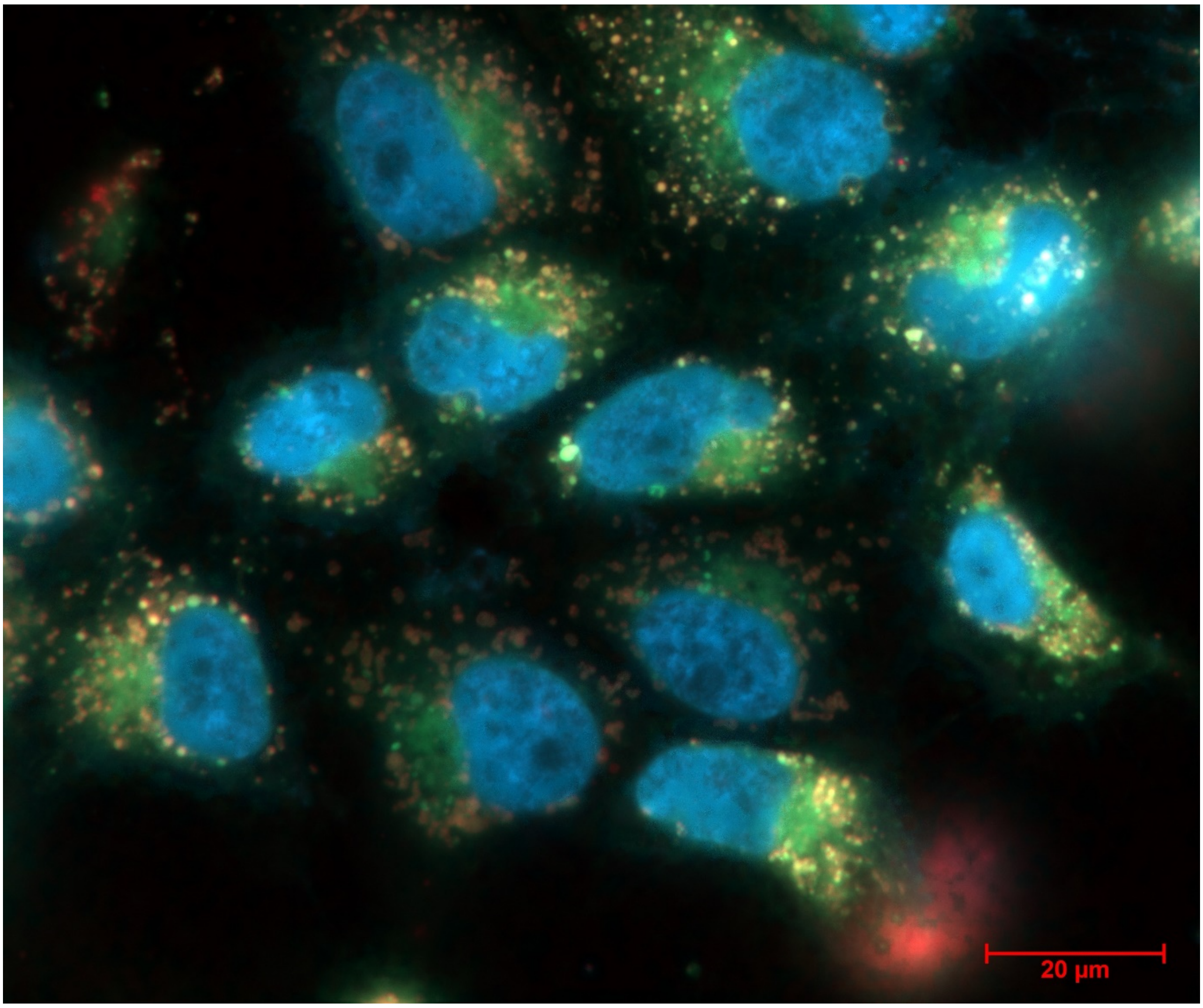

Compound 25_AHCS2

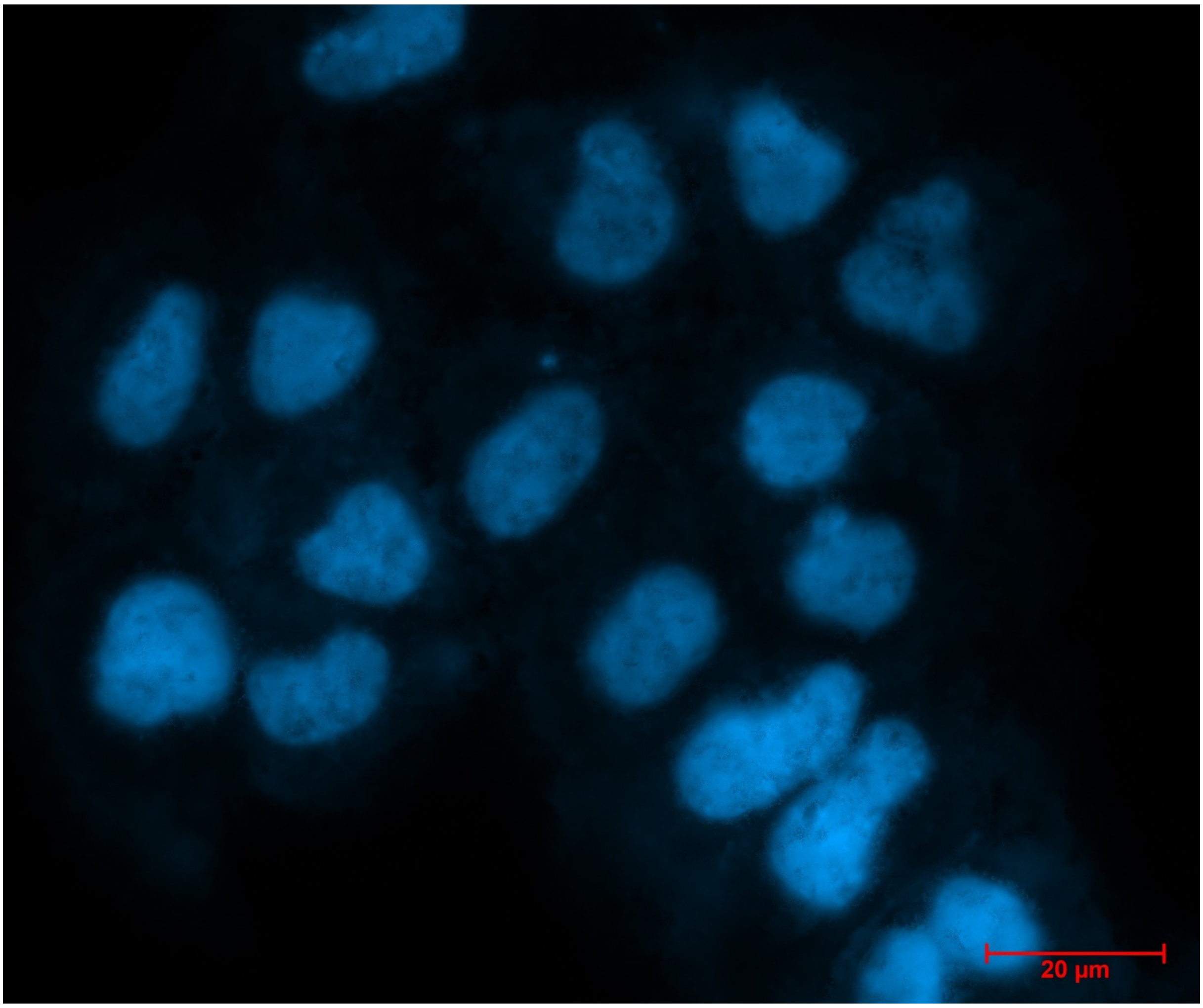

Compound 25_DAPI

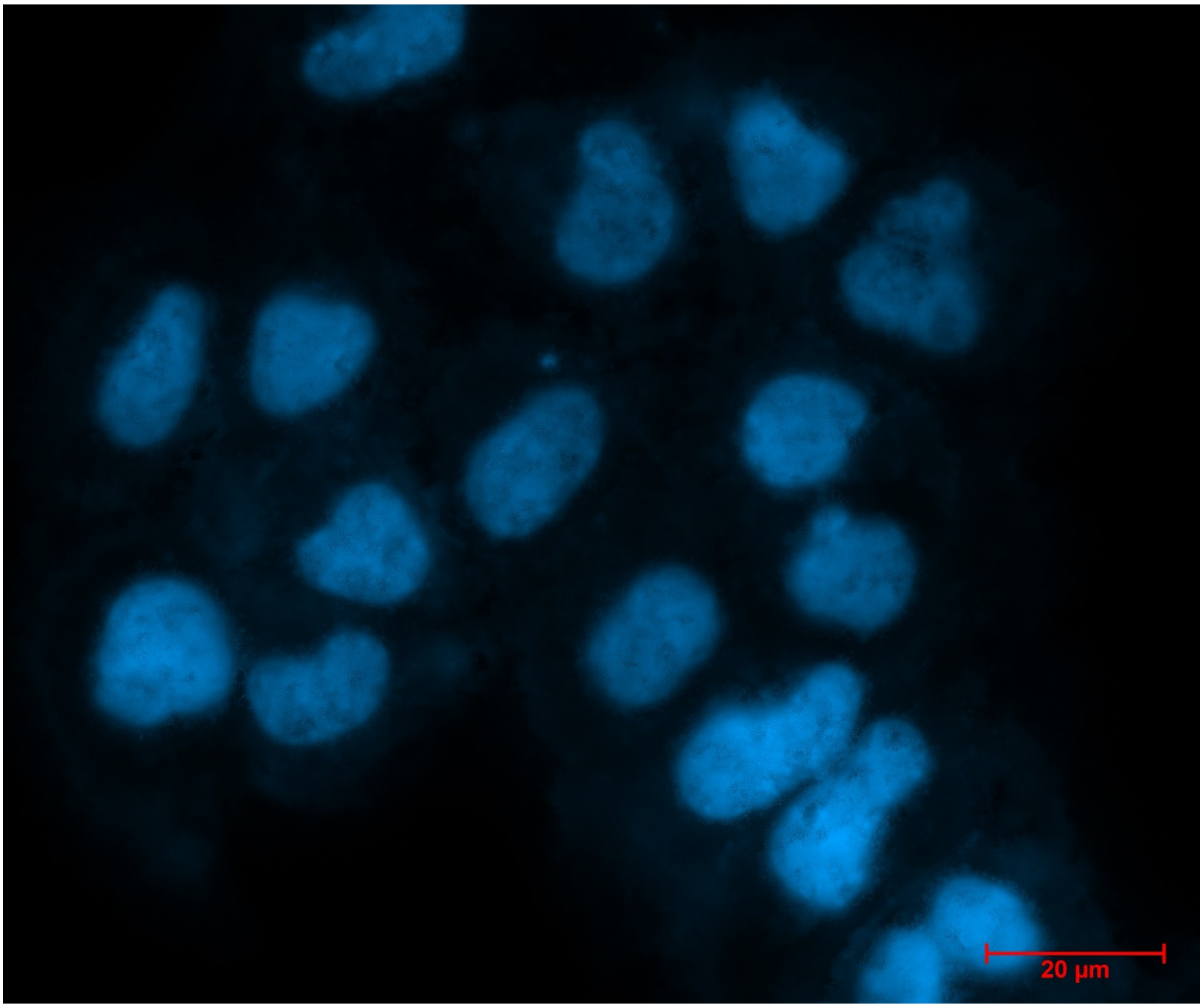

Compound 25_green

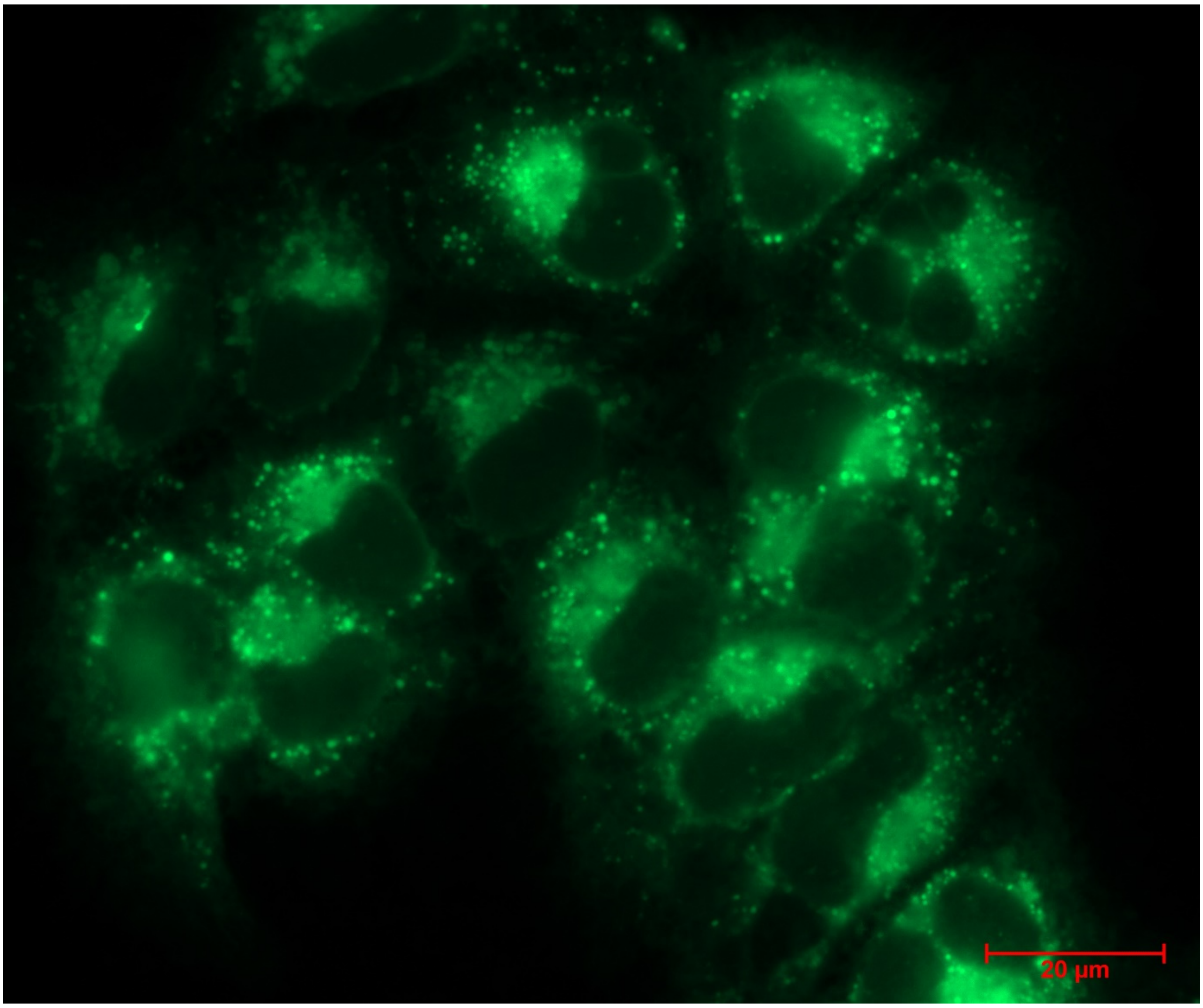

Compound 25_merged

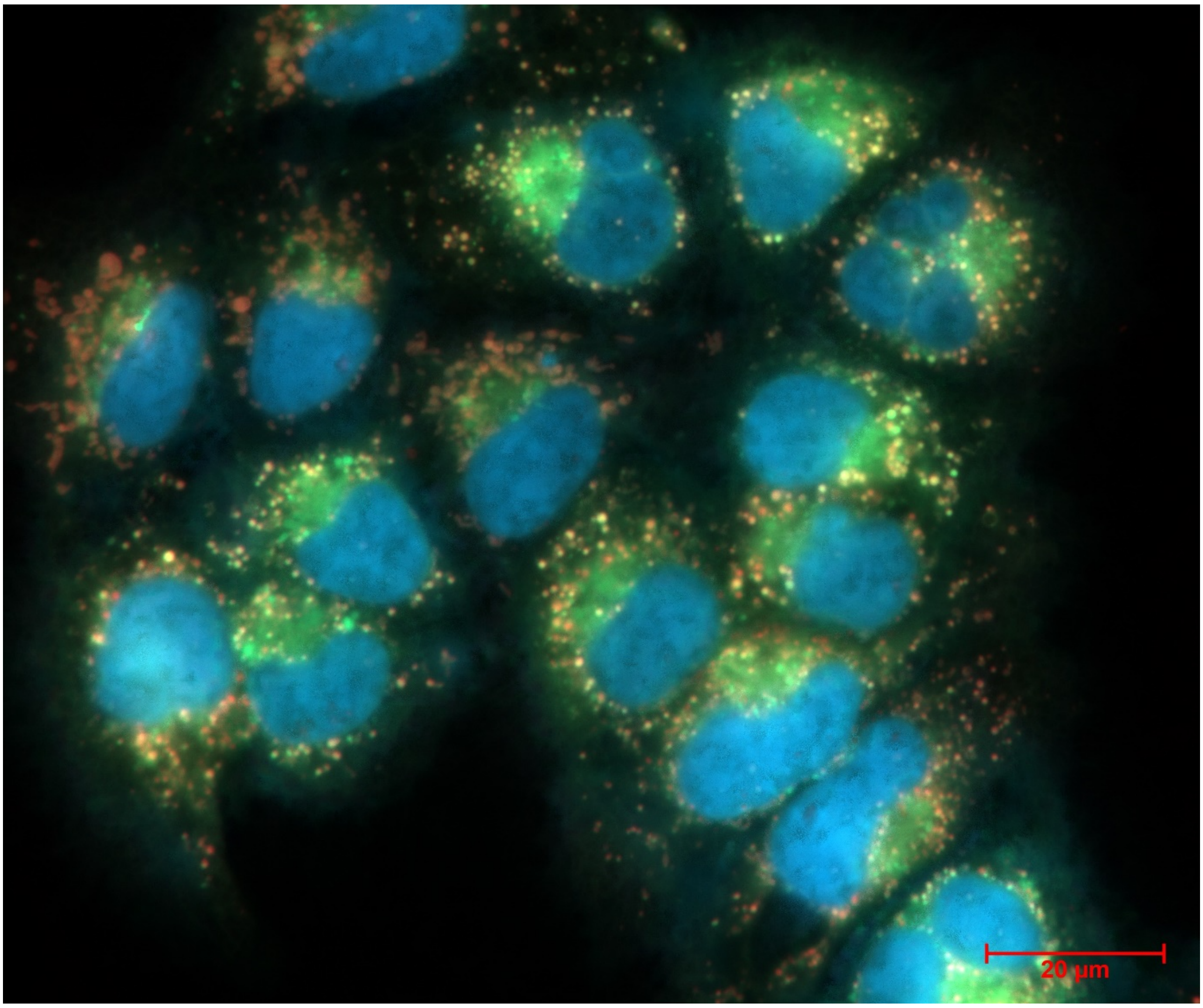

Compound 5_AHCS2

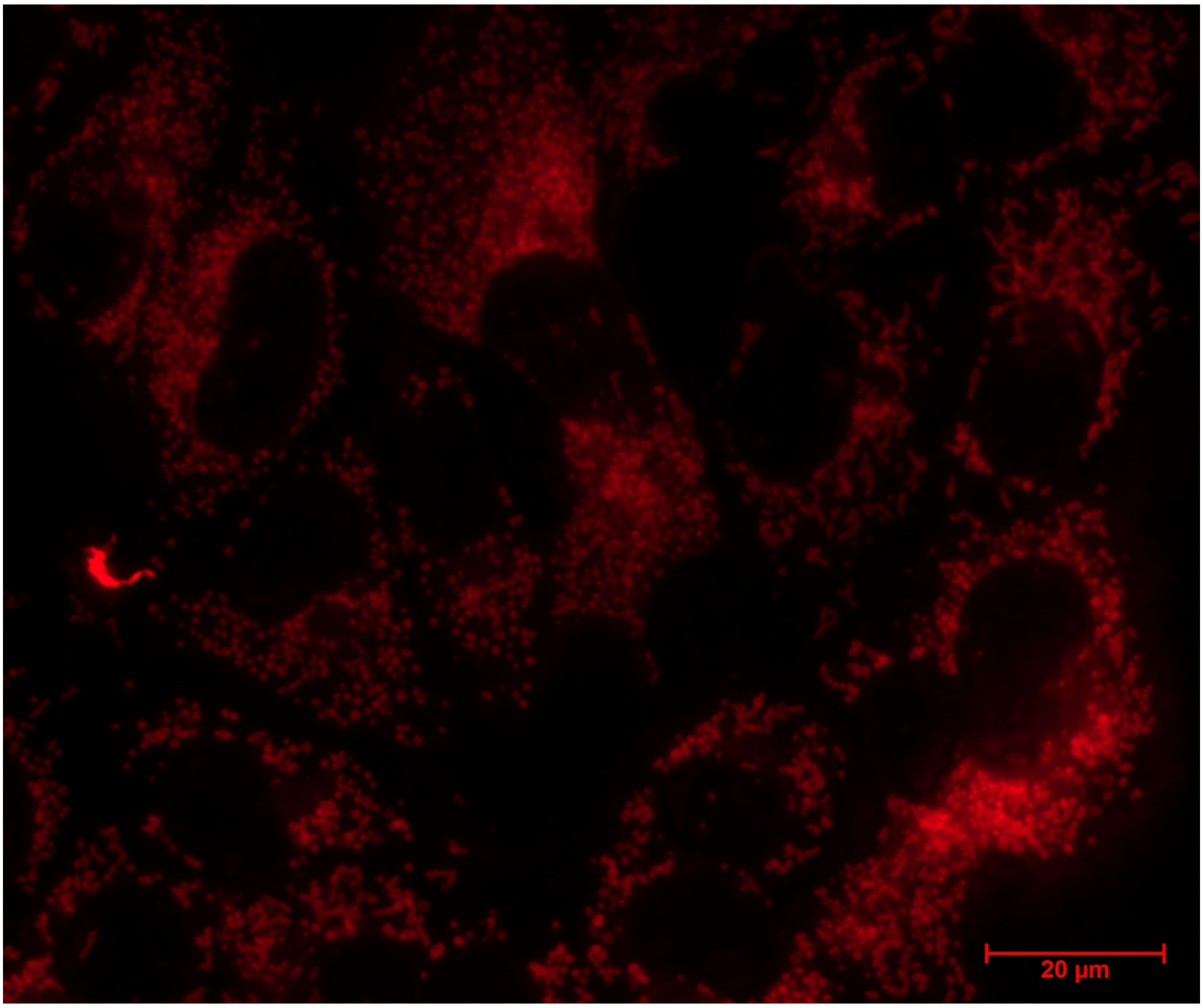

Compound 5_DAPI

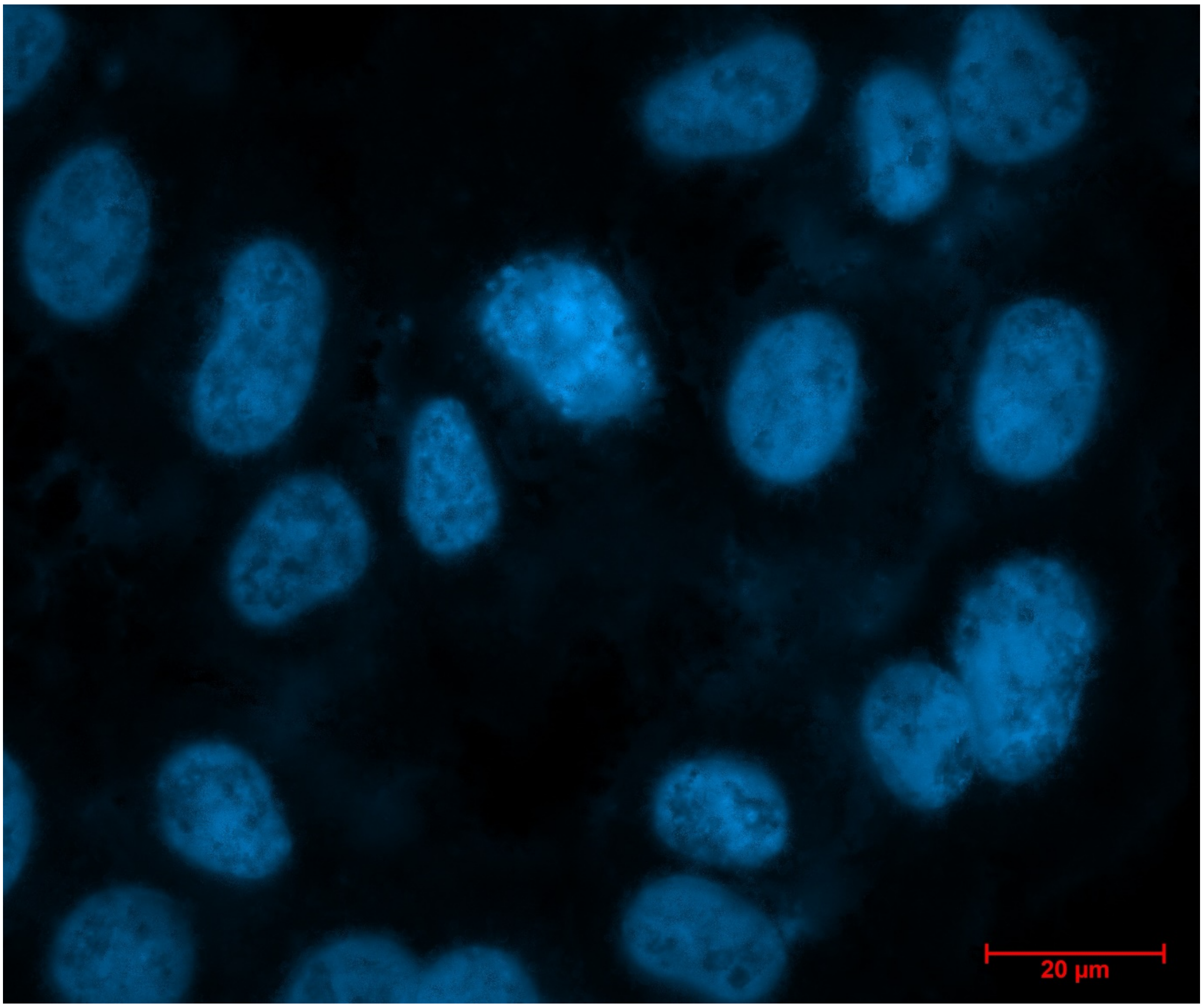

Compound 5_green

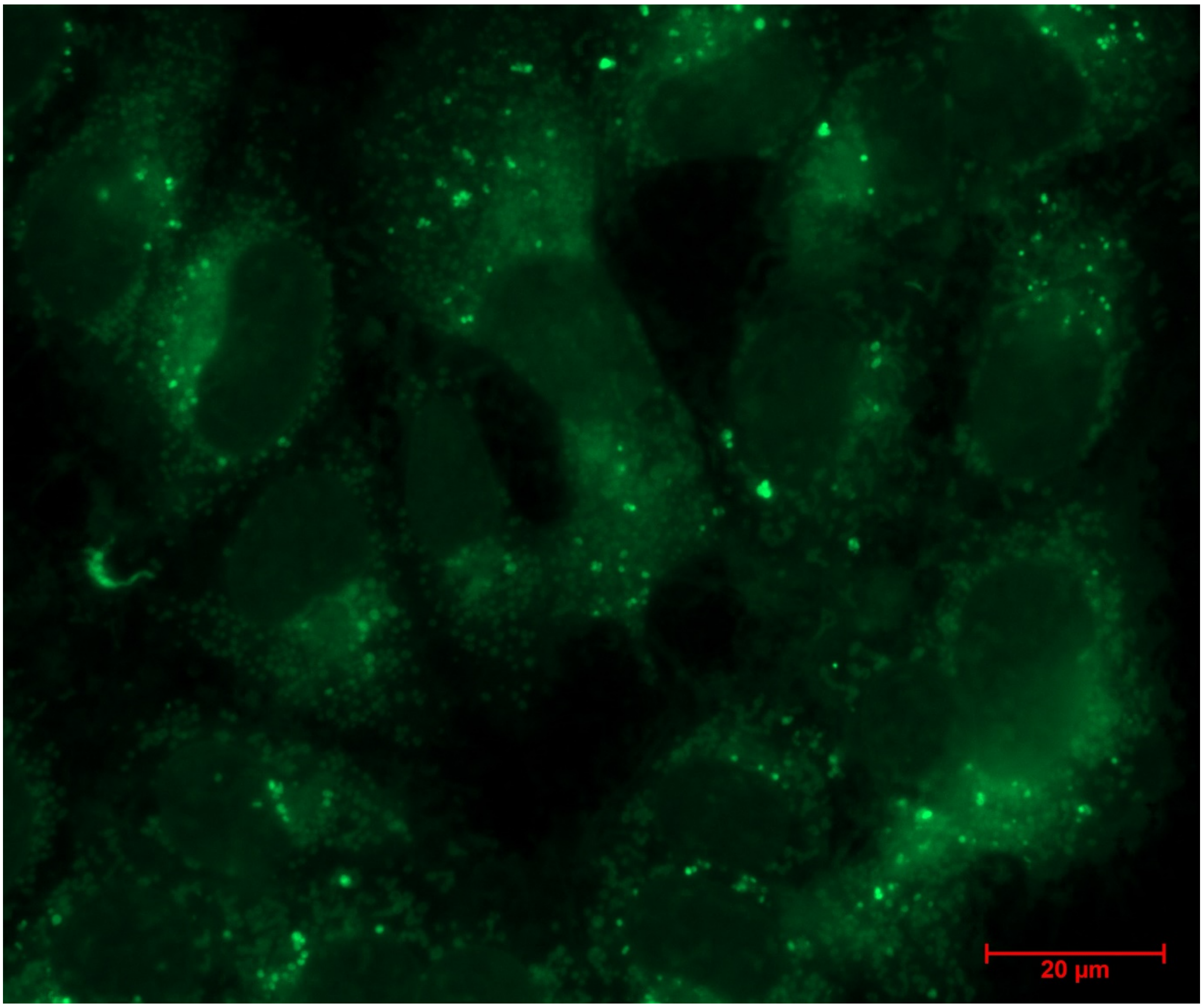

Compound 5_merged

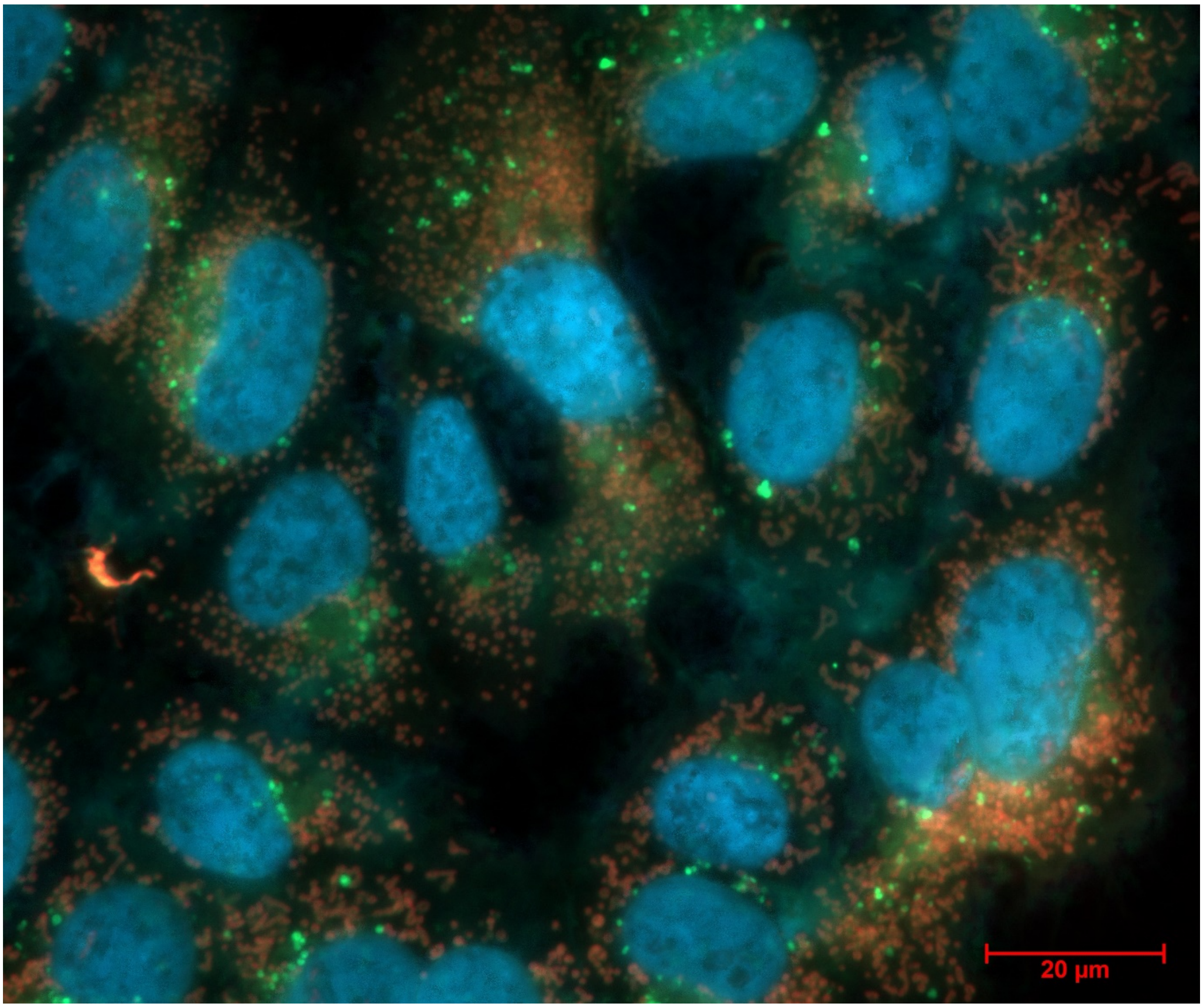

Selected NMR spectra

Spectra for 1

^{1}H NMR (400 MHz, DMSO-d_6)

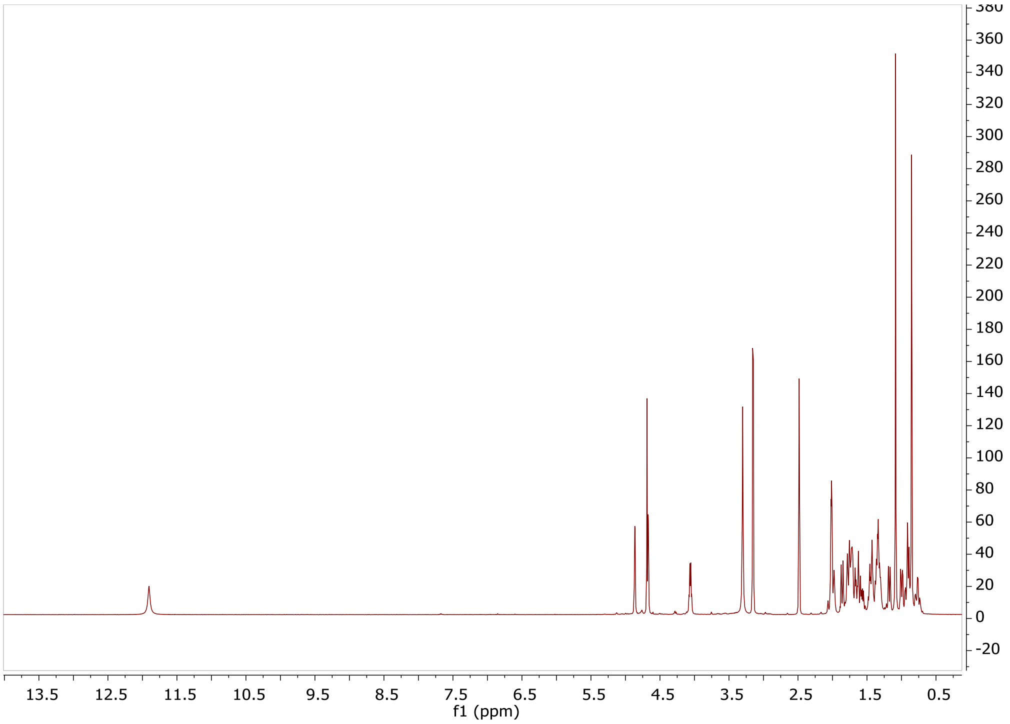

^{13}C NMR (APT, 101 MHz, DMSO-d_6)

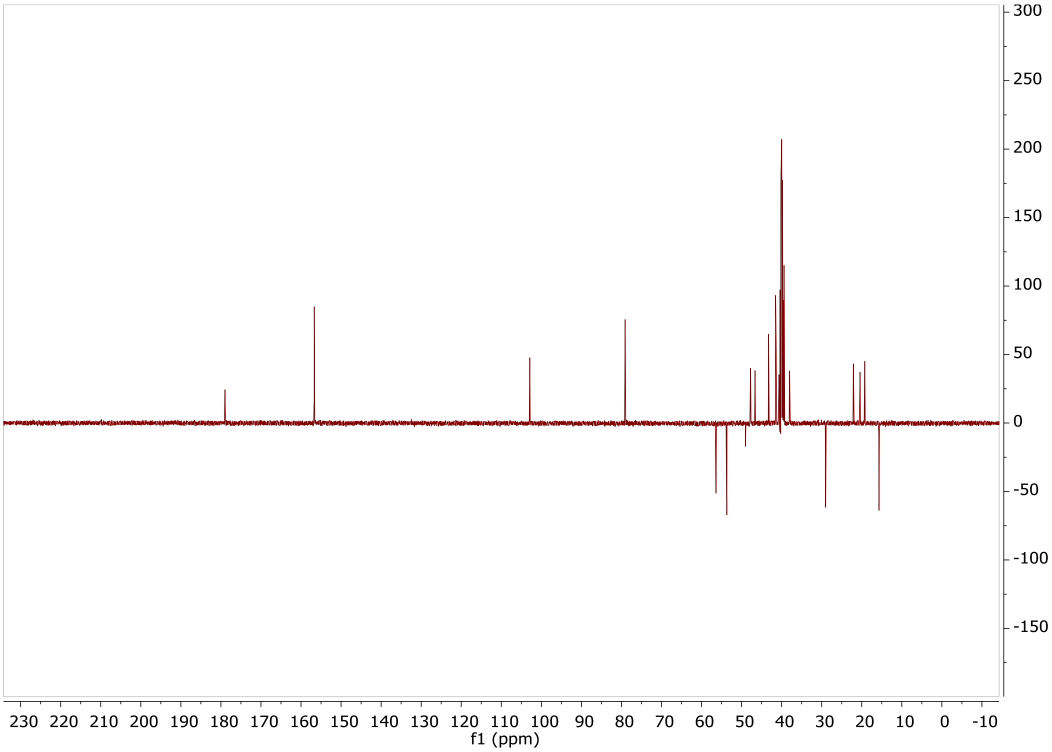

Spectra for 2

^{1}H NMR (400 MHz, chloroform-d$_3$)

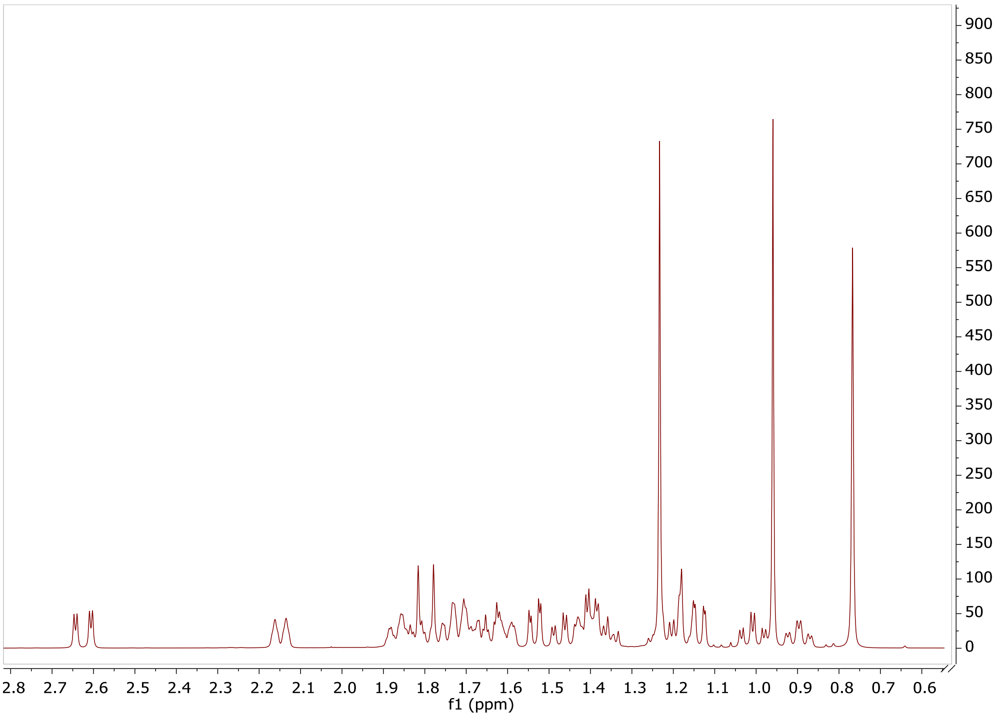

^{13}C NMR (APT, 101 MHz, chloroform-d$_3$)

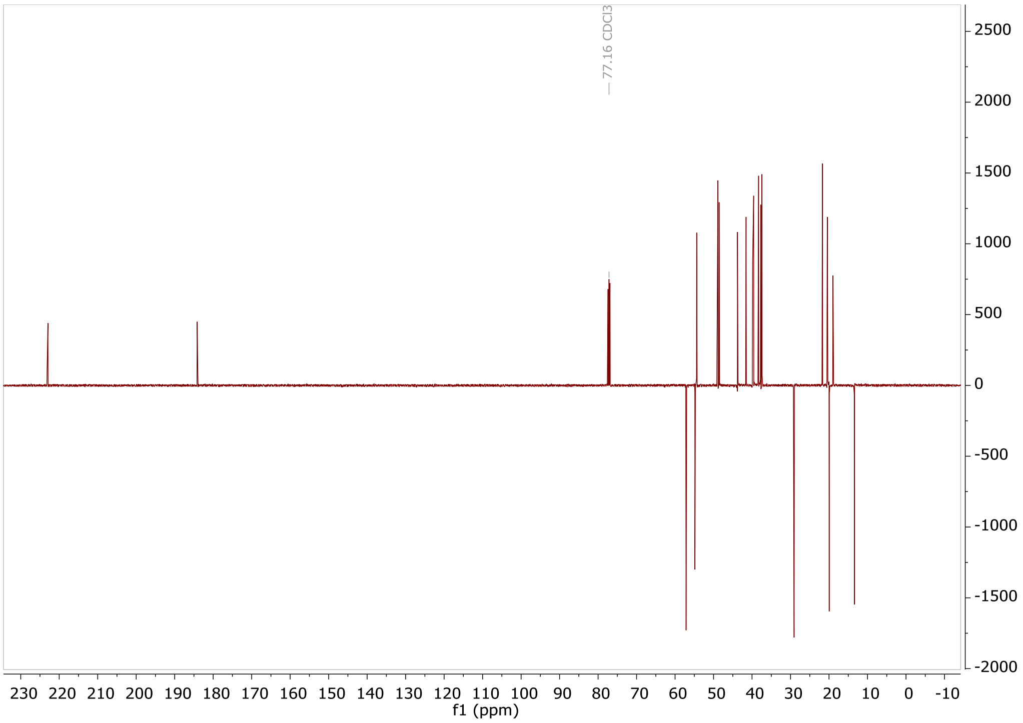

Spectra for 3

^{1}H NMR (400 MHz, DMSO-d$_6$)

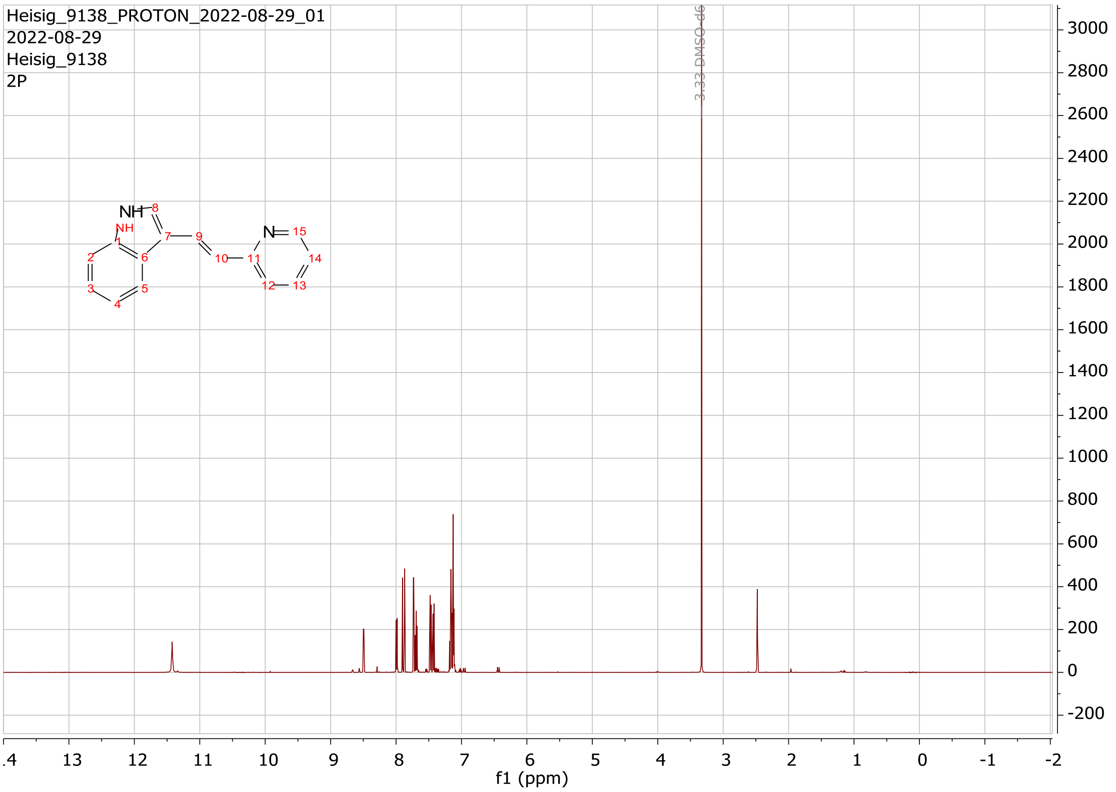

^{13}C NMR (APT, 101 MHz, DMSO-d$_6$)

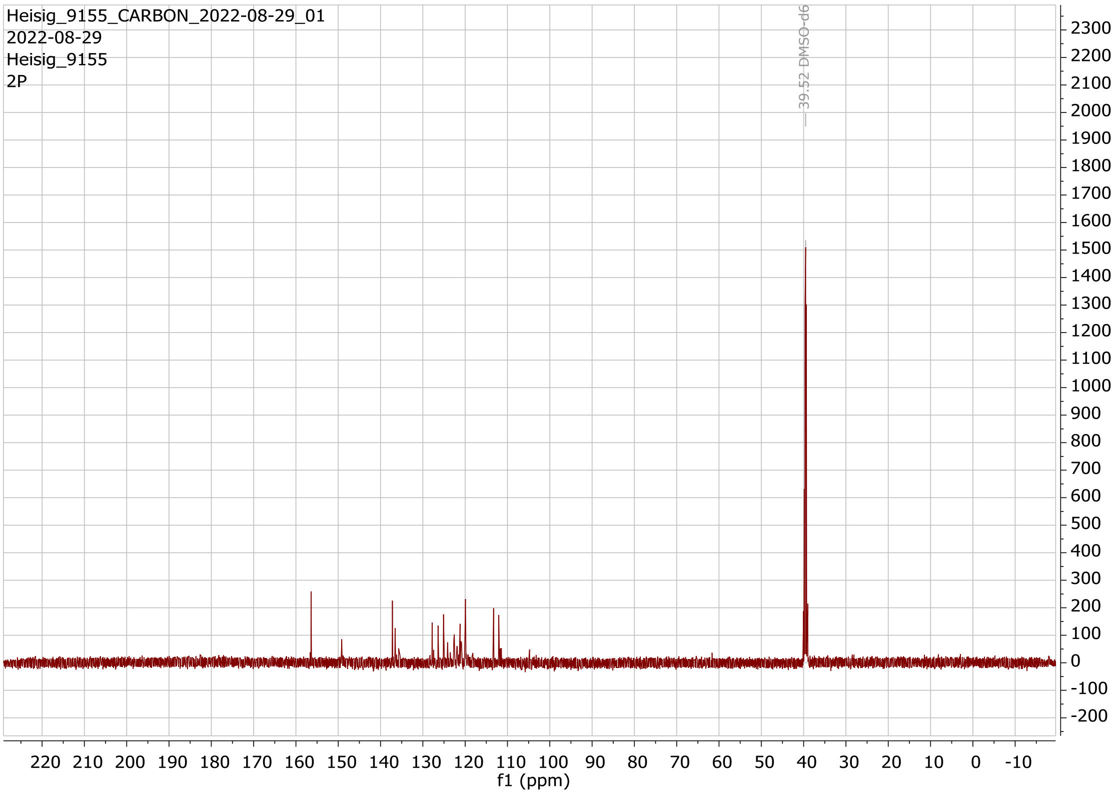

Spectra for 4

^{1}H NMR (400 MHz, DMSO-d$_6$)

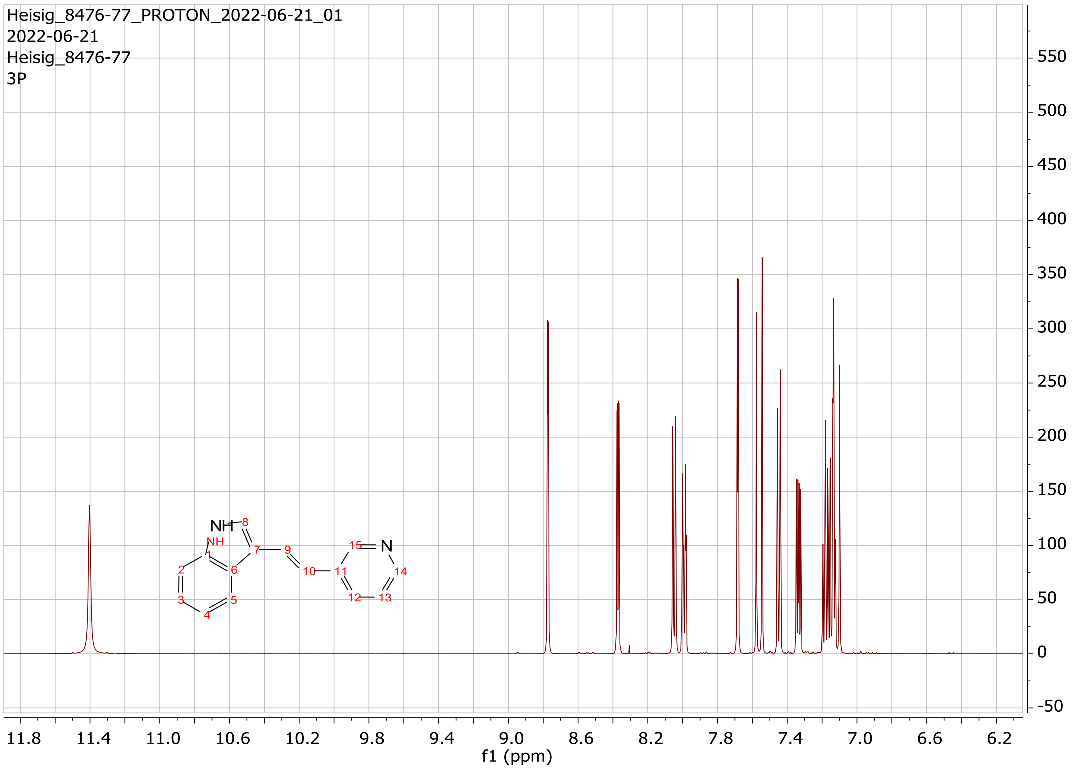

^{13}C NMR (APT, 101 MHz, DMSO-d$_6$)

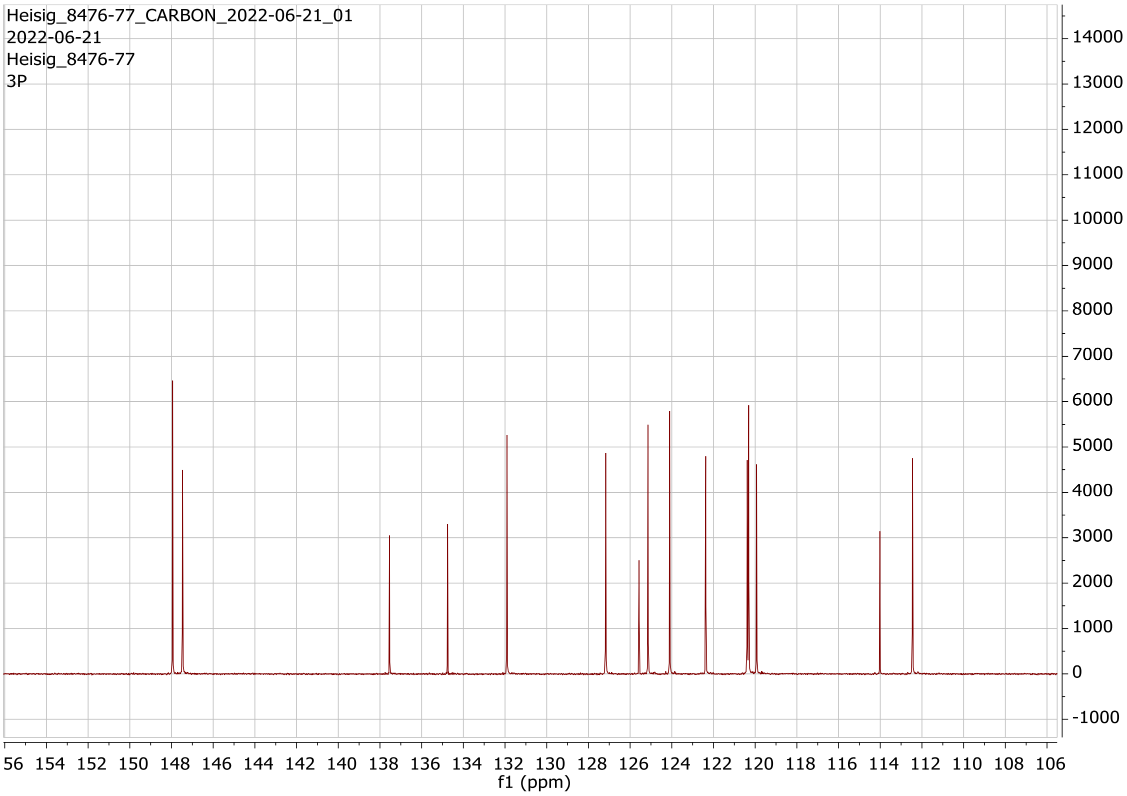

Spectra for 5

^1H NMR (400 MHz, DMSO-d_6)

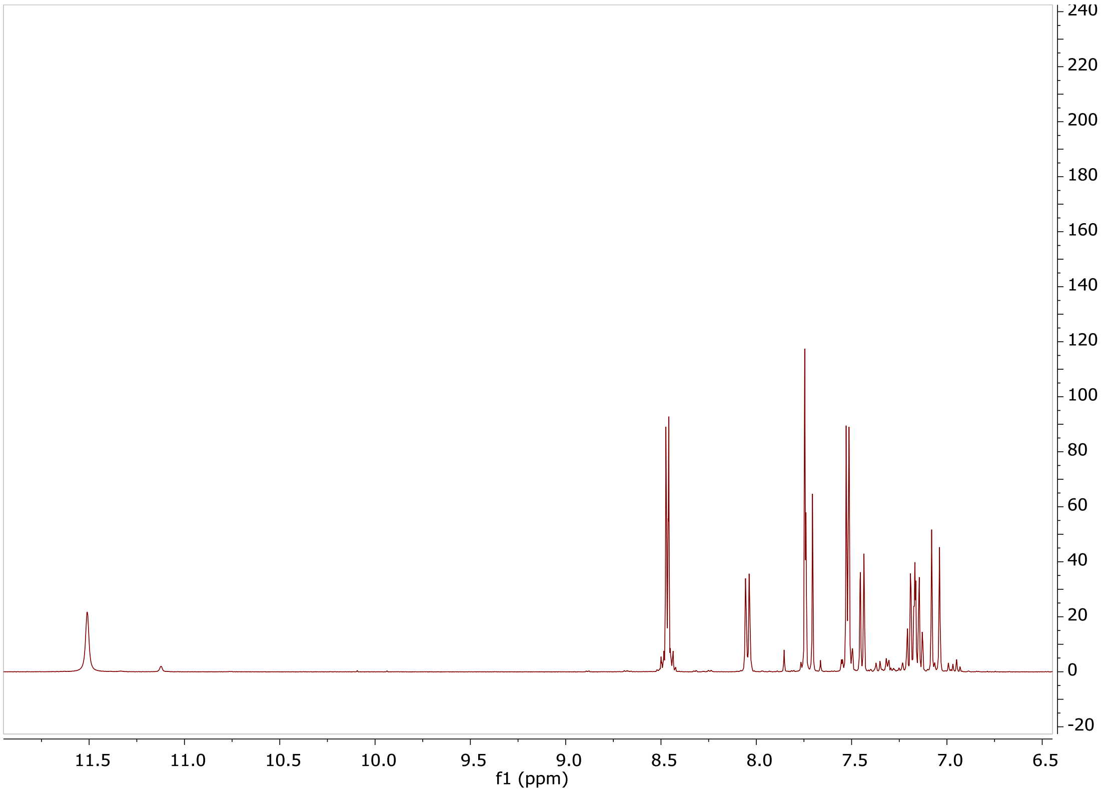

^{13}C NMR (APT, 101 MHz, DMSO-d_6)

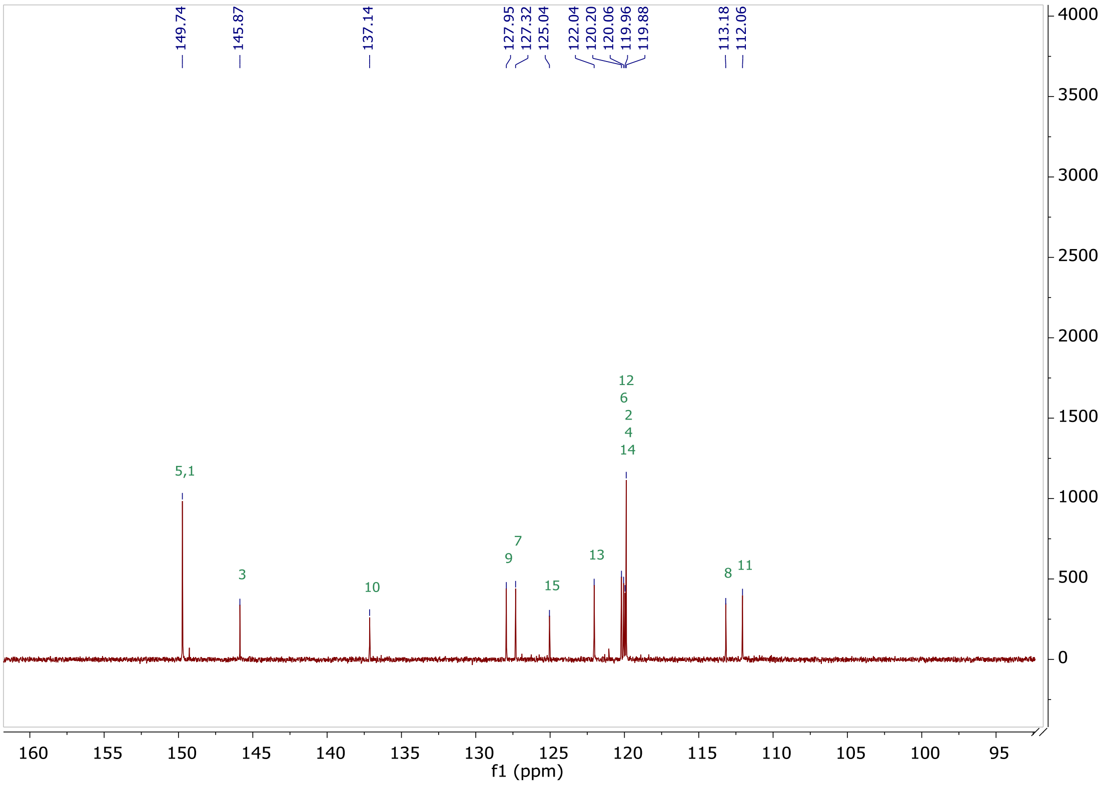

Spectra for 6

^{1}H NMR (400 MHz, chloroform-d_3)

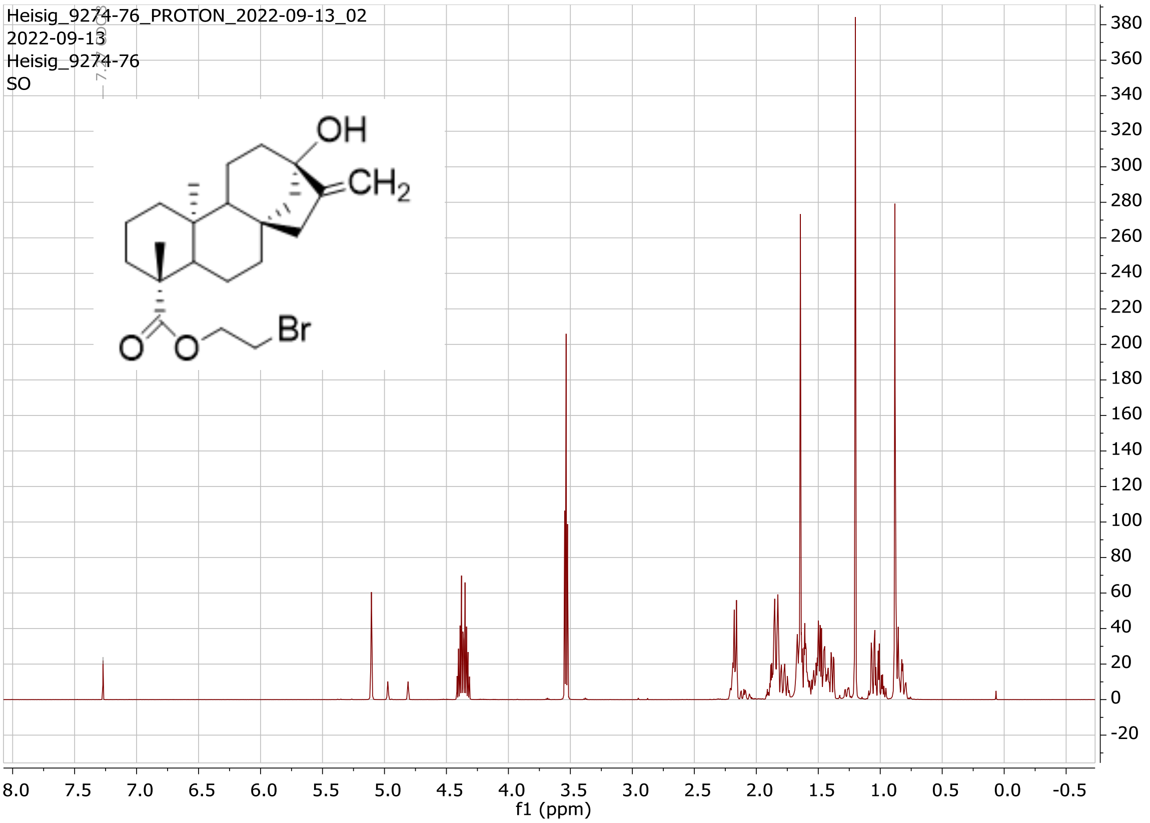

^{13}C NMR (APT, 101 MHz, chloroform-d_3)

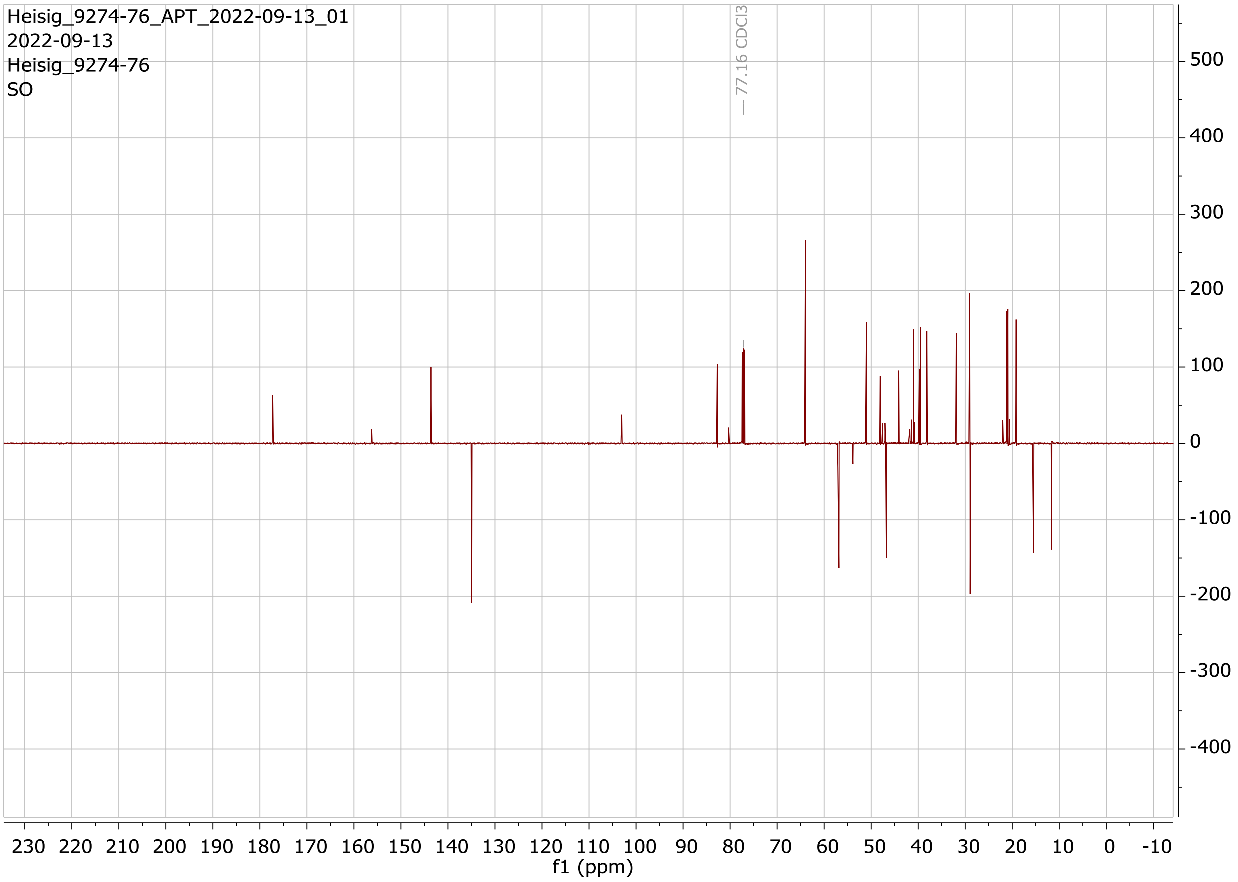

Spectra for 7

^1H NMR (400 MHz, chloroform-d_3)

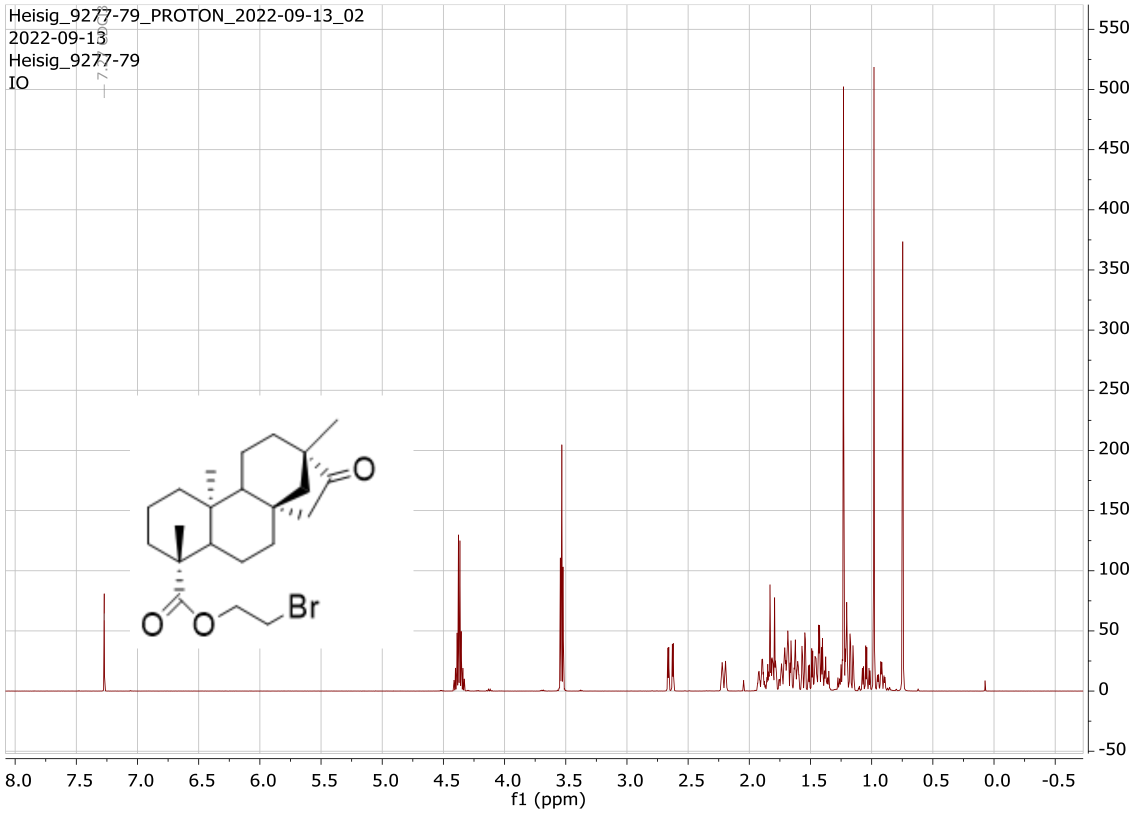

^{13}C NMR (APT, 101 MHz, chloroform-d_3)

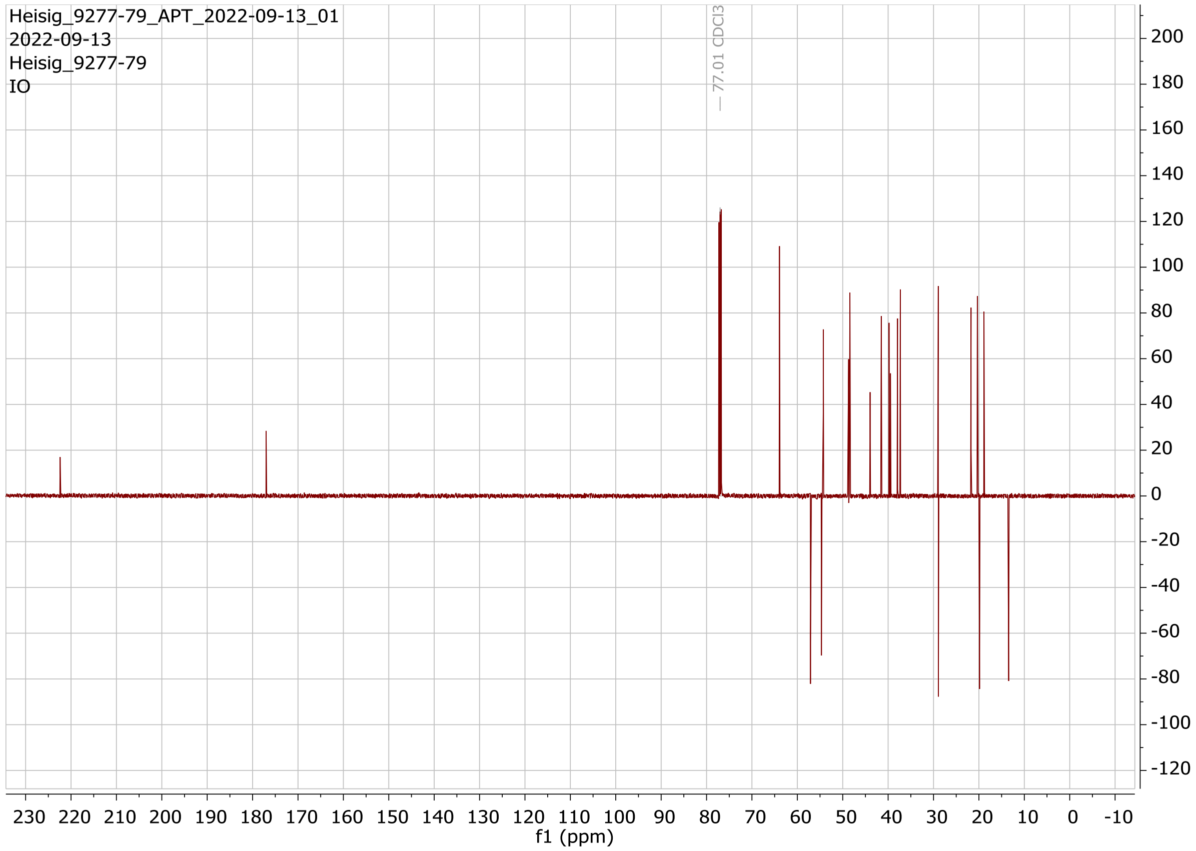

Spectra for 8

^{1}H NMR (400 MHz, chloroform-d$_3$)

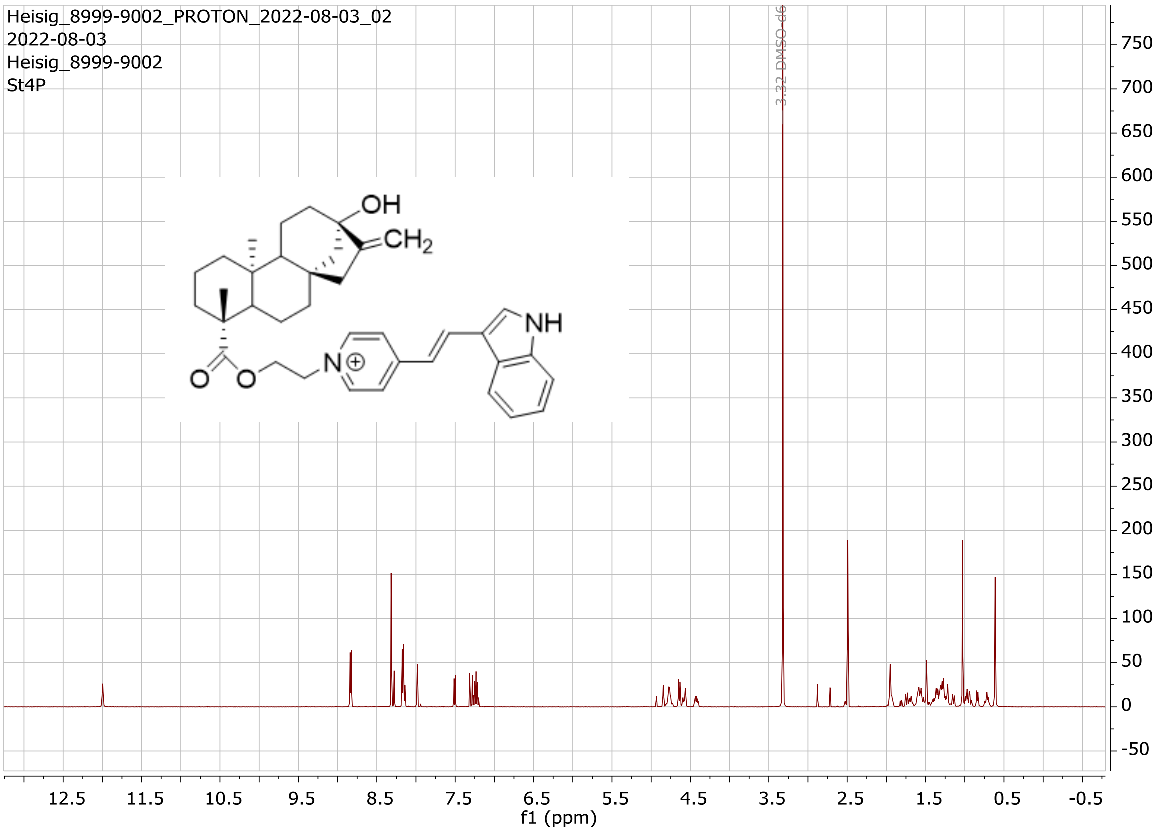

^{13}C NMR (APT, 101 MHz, chloroform-d$_3$)

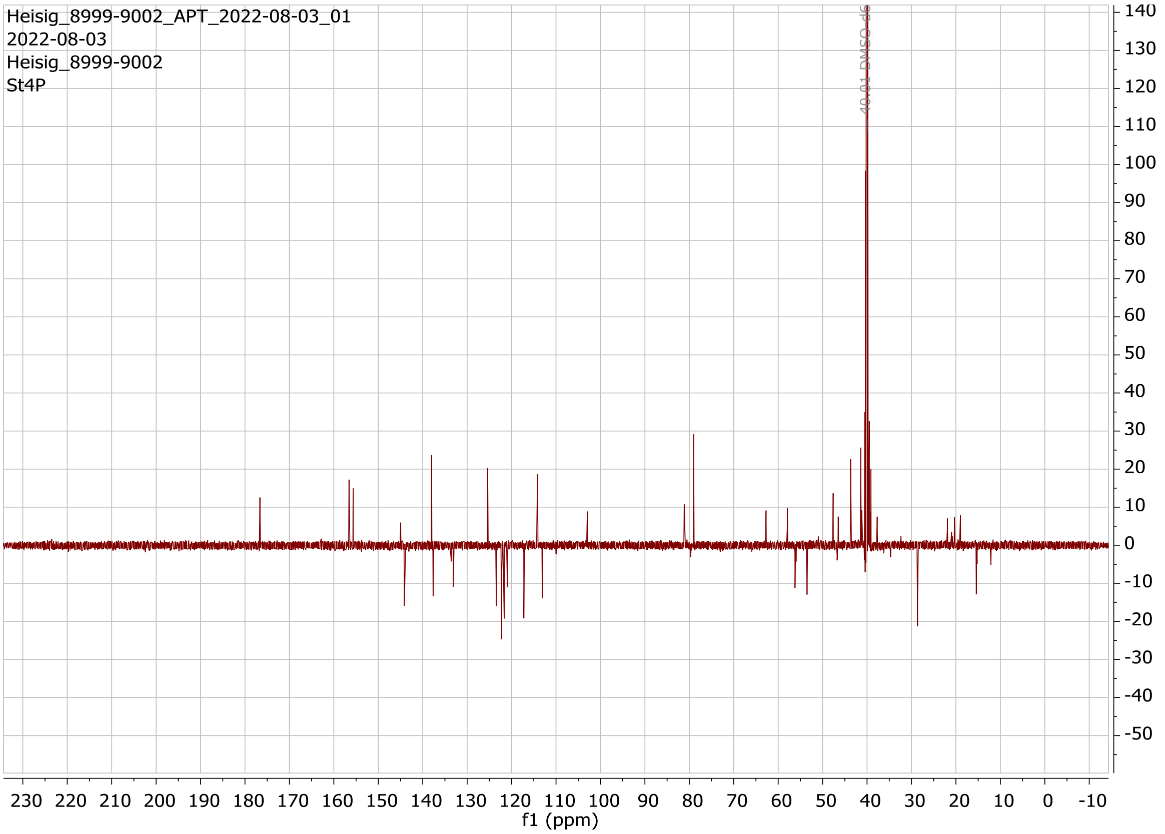

Spectra for 13

^{1}H NMR (400 MHz, DMSO-d_6)

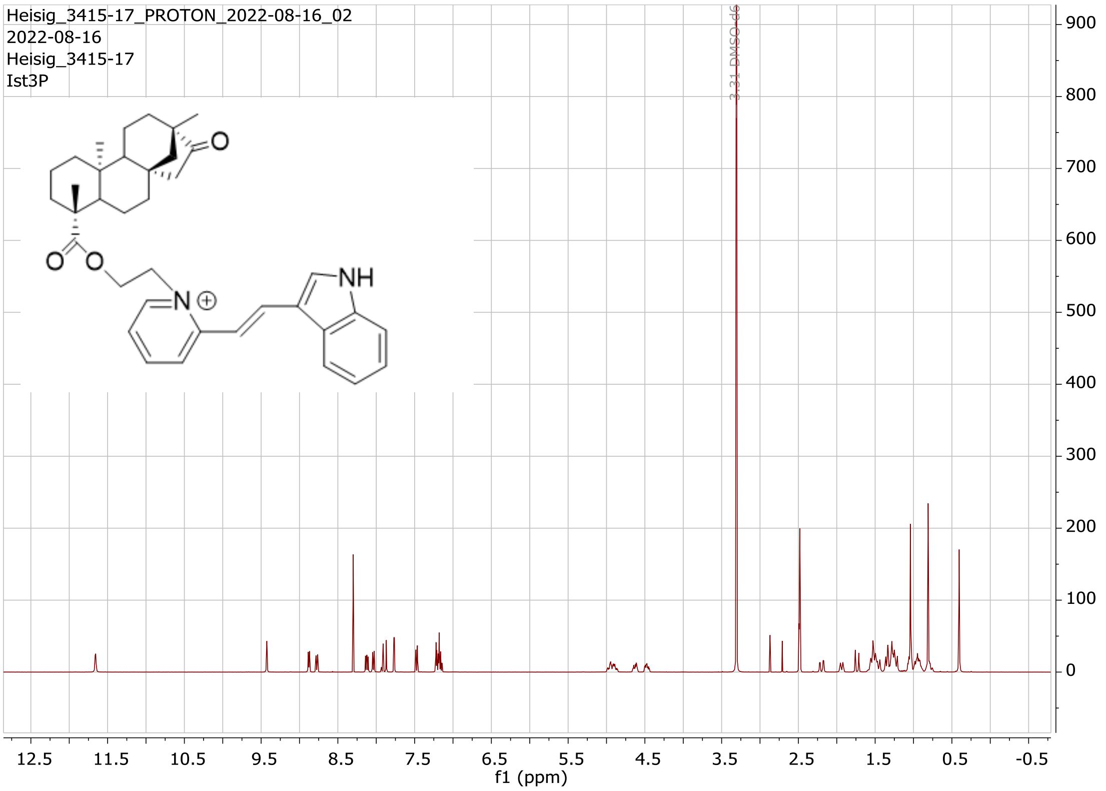

^{13}C NMR (APT, 101 MHz, DMSO-d_6)

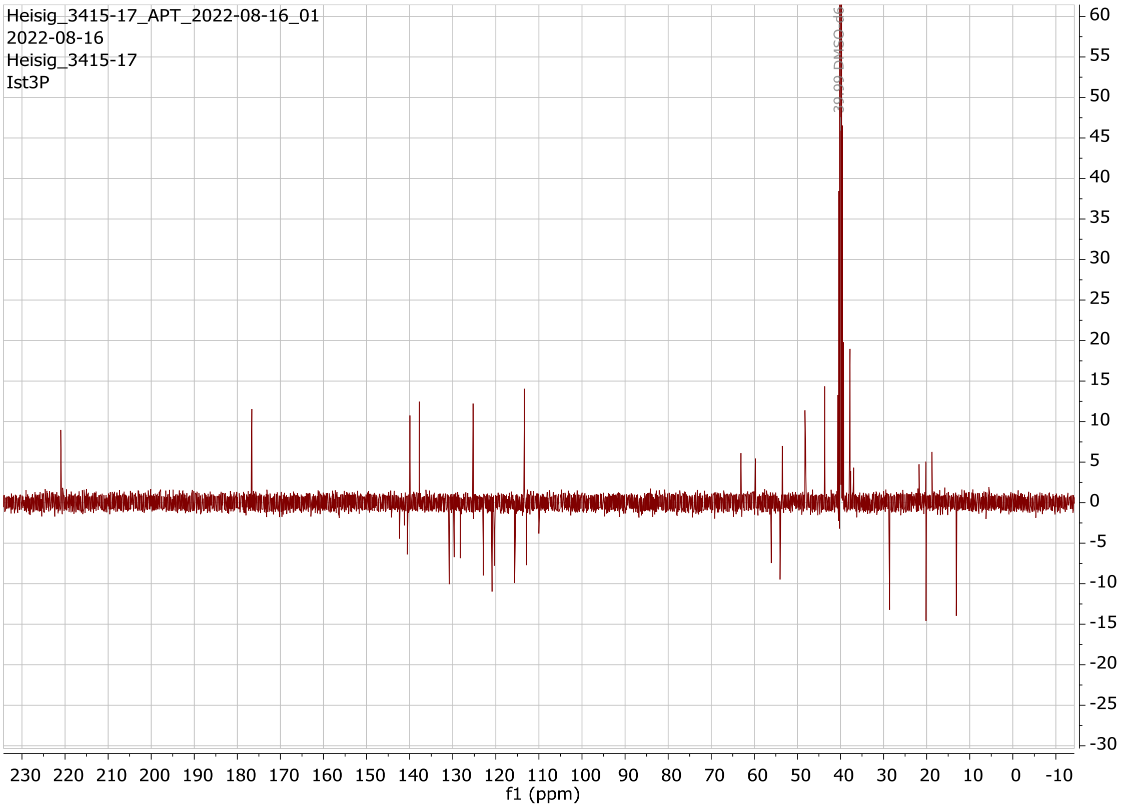

Spectra for 15

^{1}H NMR (400 MHz, chloroform-d$_3$)

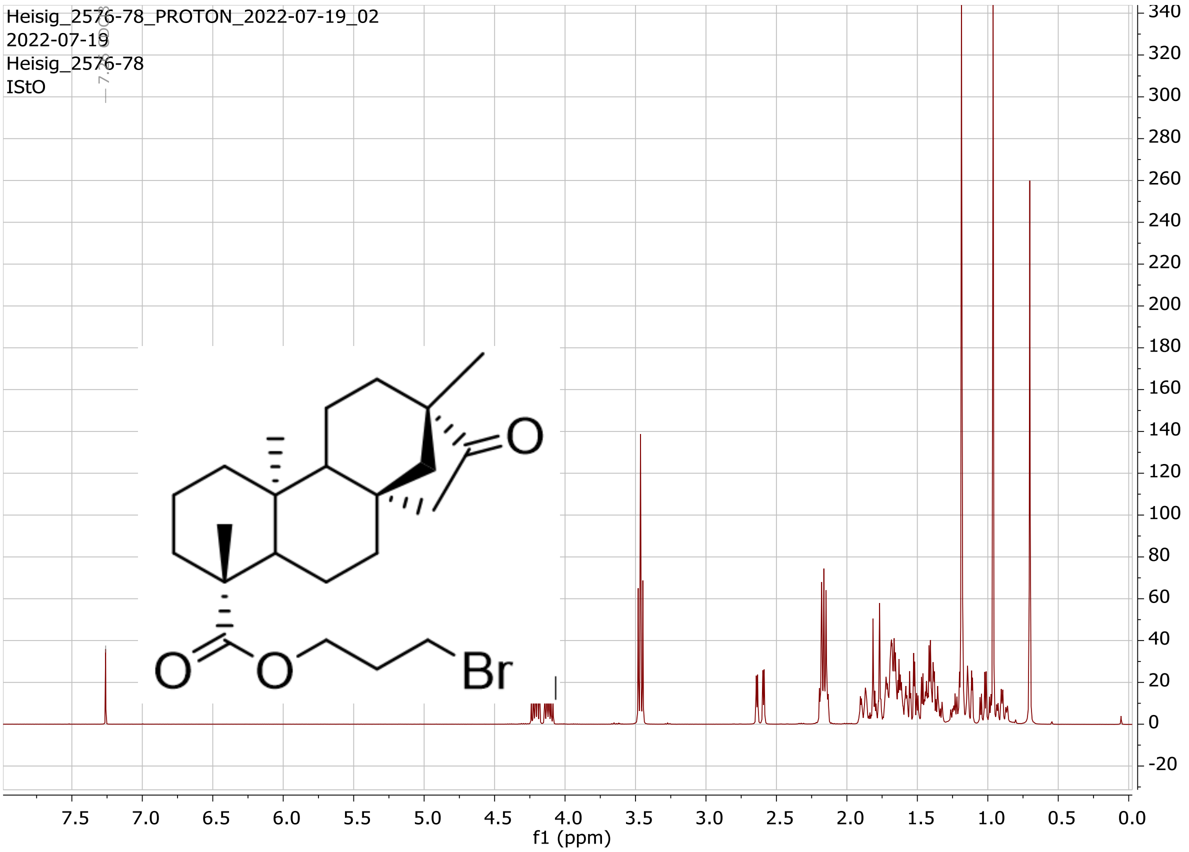

^{13}C NMR (APT, 101 MHz, chloroform-d$_3$)

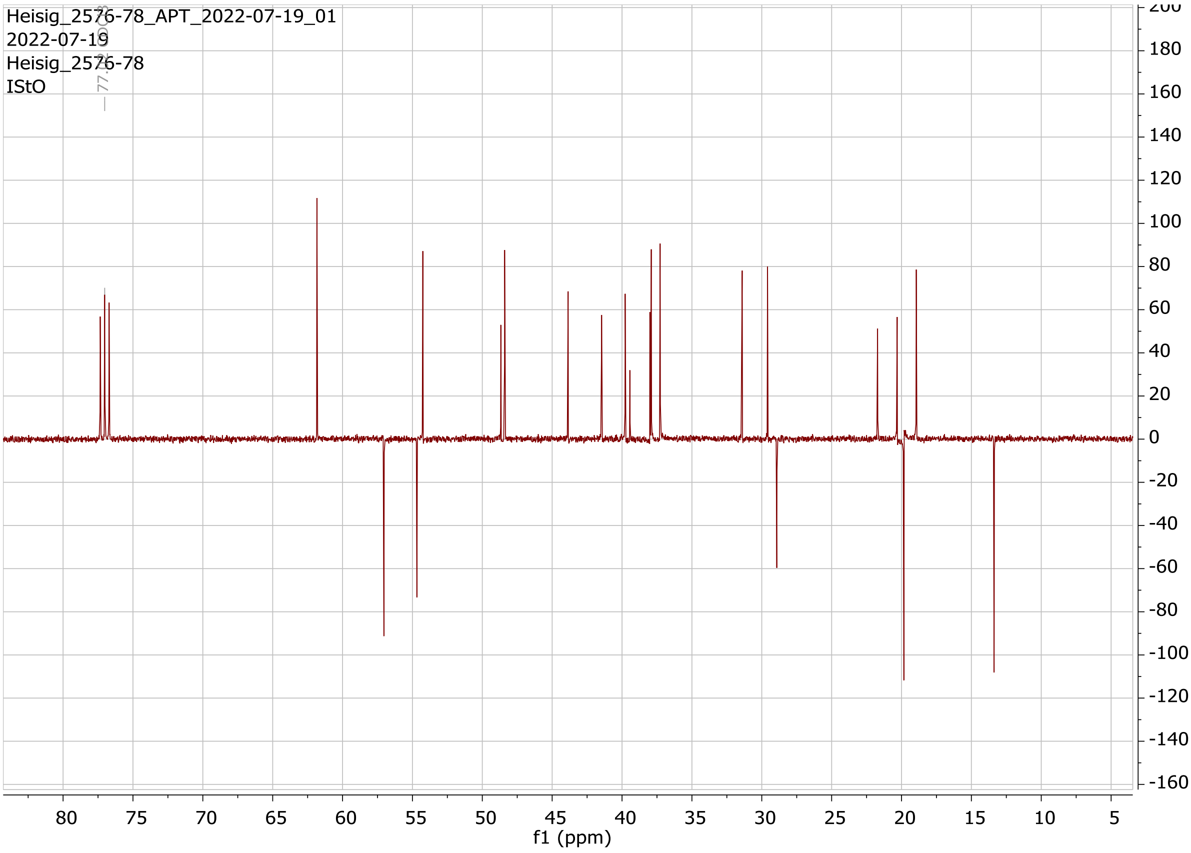

Spectra for 20

^{1}H NMR (400 MHz, DMSO-d_6)

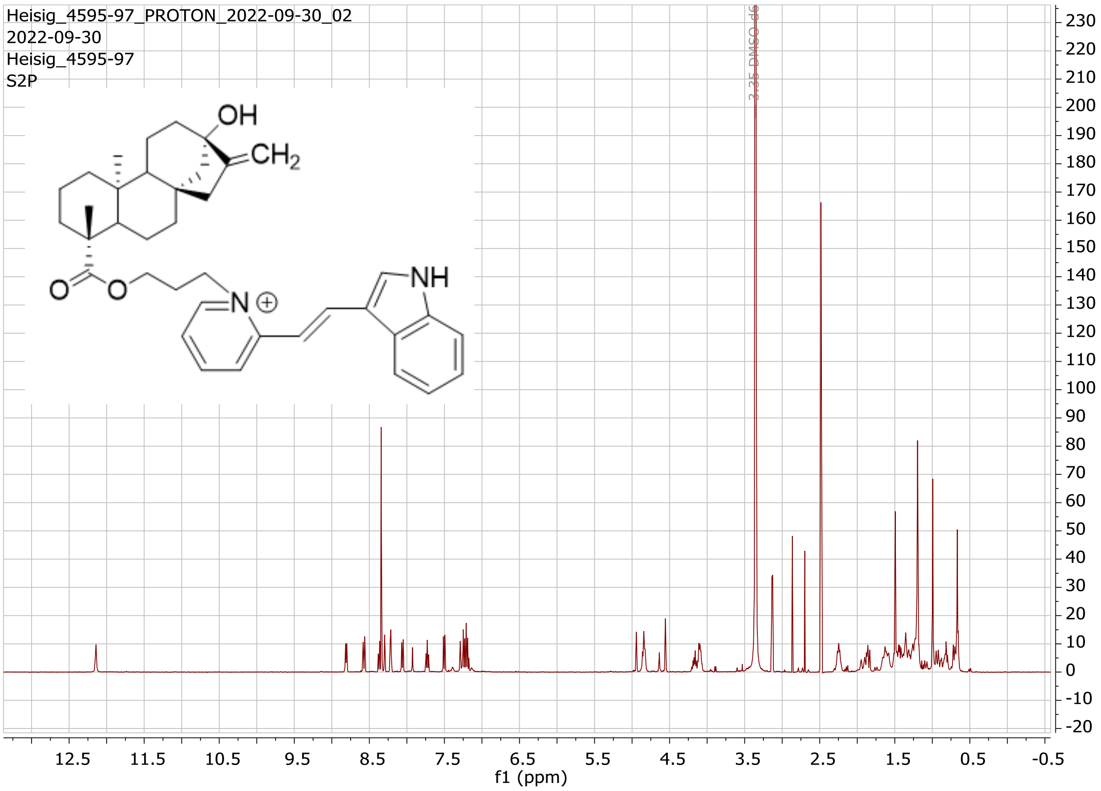

^{13}C NMR (APT, 101 MHz, DMSO-d_6)

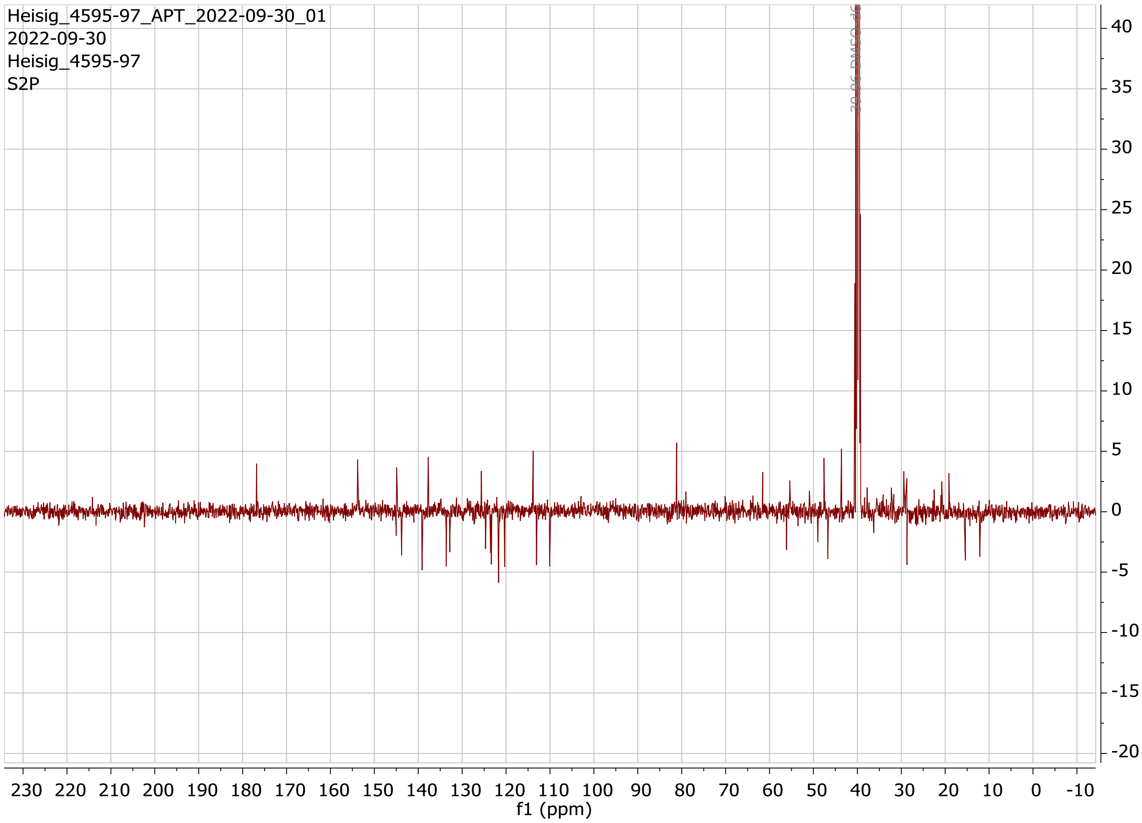

Spectra for 23

^{1}H NMR (400 MHz, DMSO-d_6)

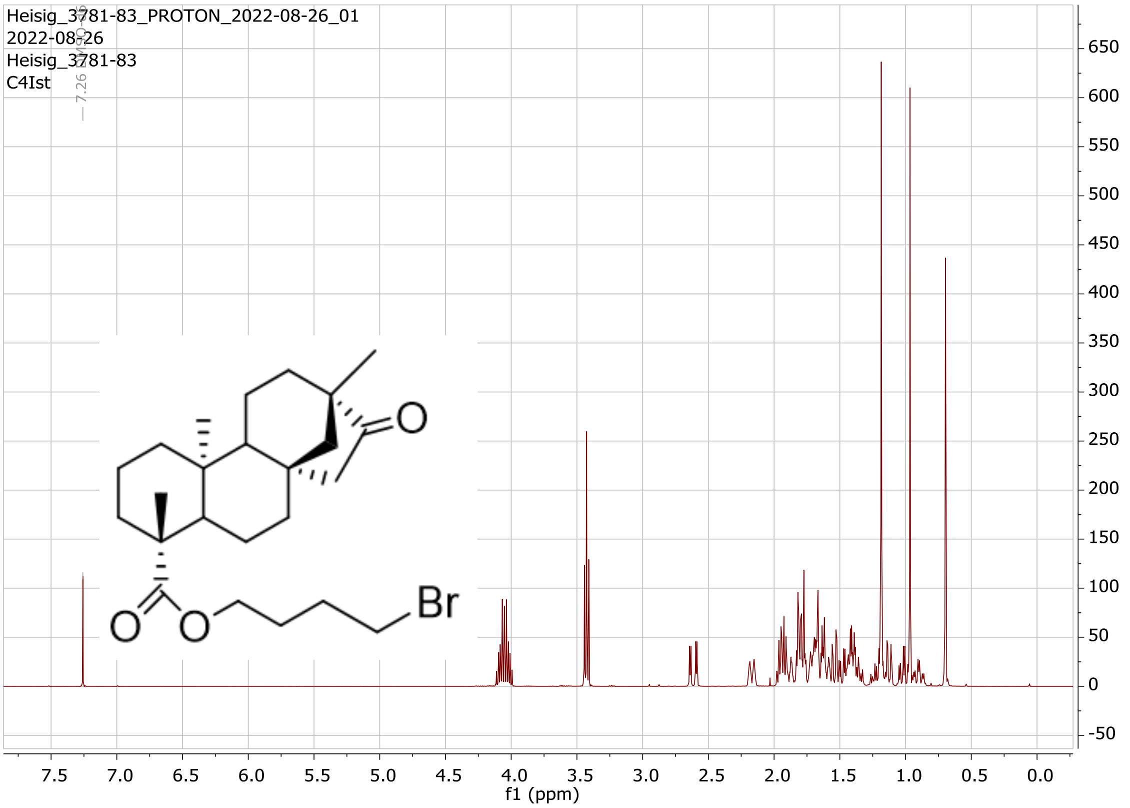

^{13}C NMR (APT, 101 MHz, DMSO-d_6)

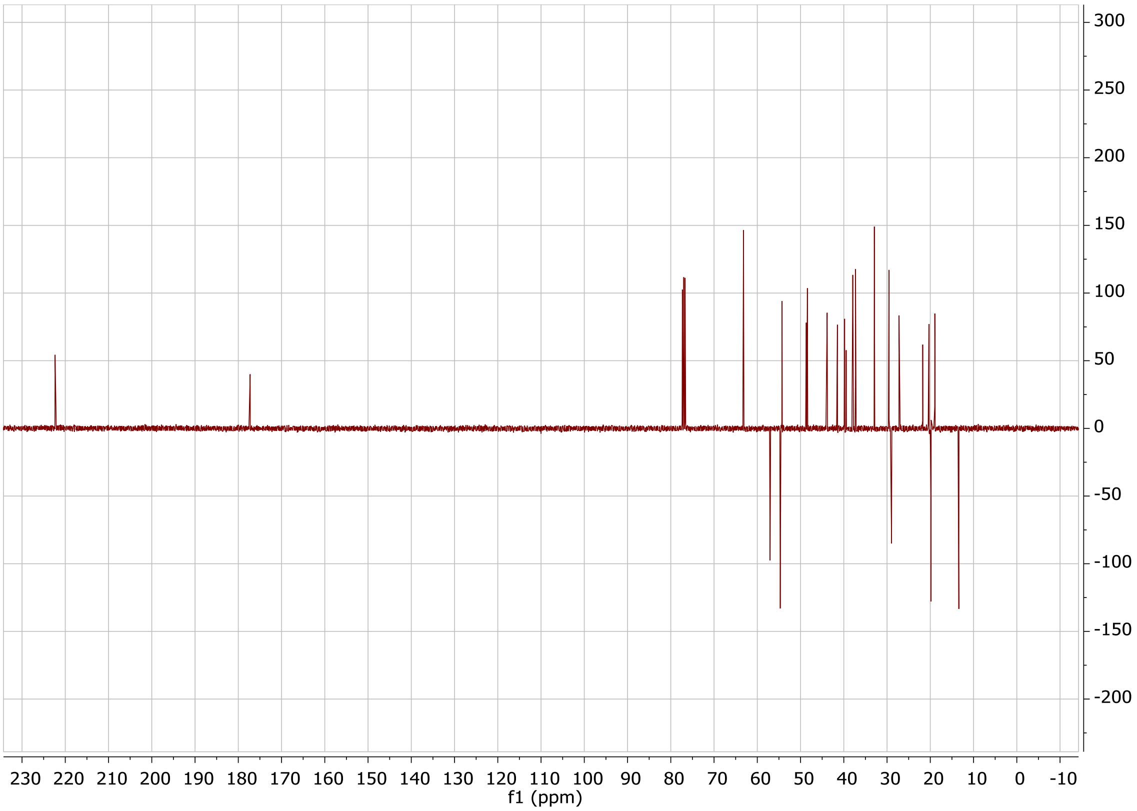

Spectra for 24

^1H NMR (400 MHz, DMSO-d_6)

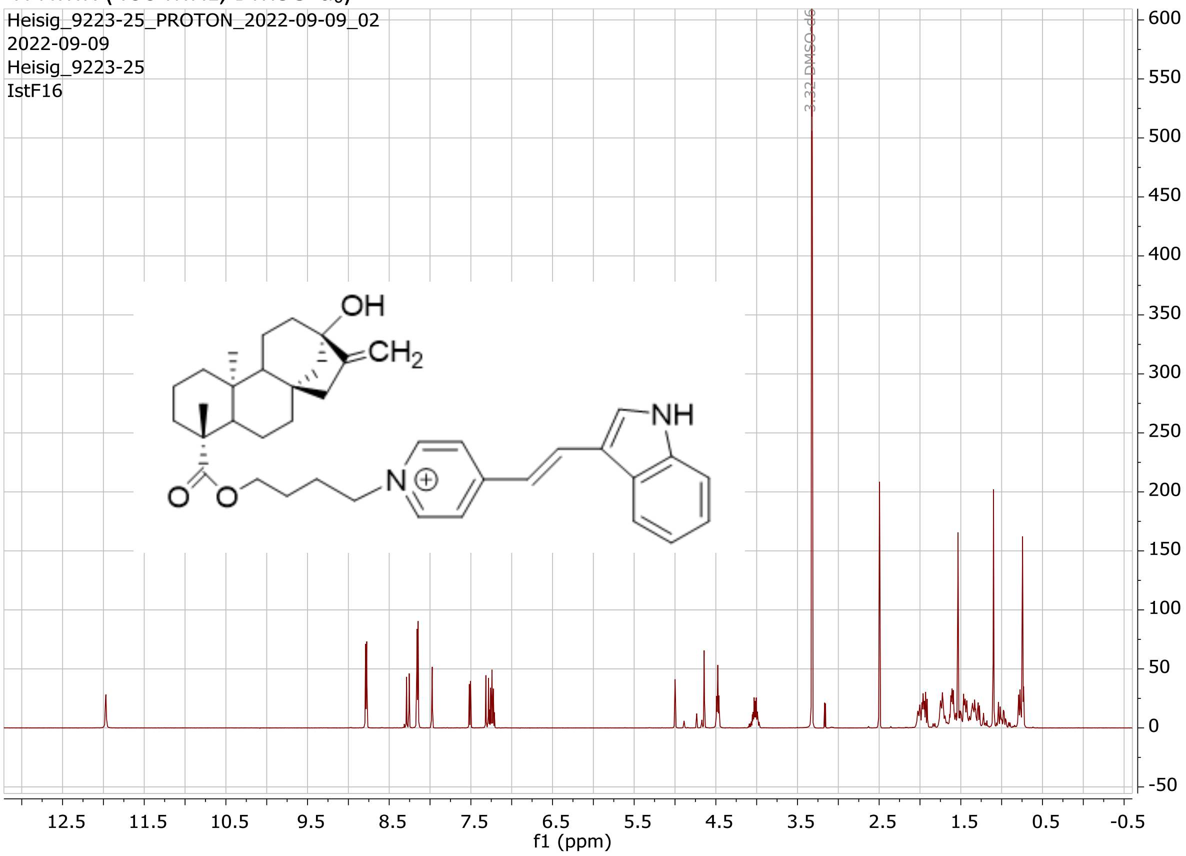

^{13}C NMR (APT, 101 MHz, DMSO-d_6)

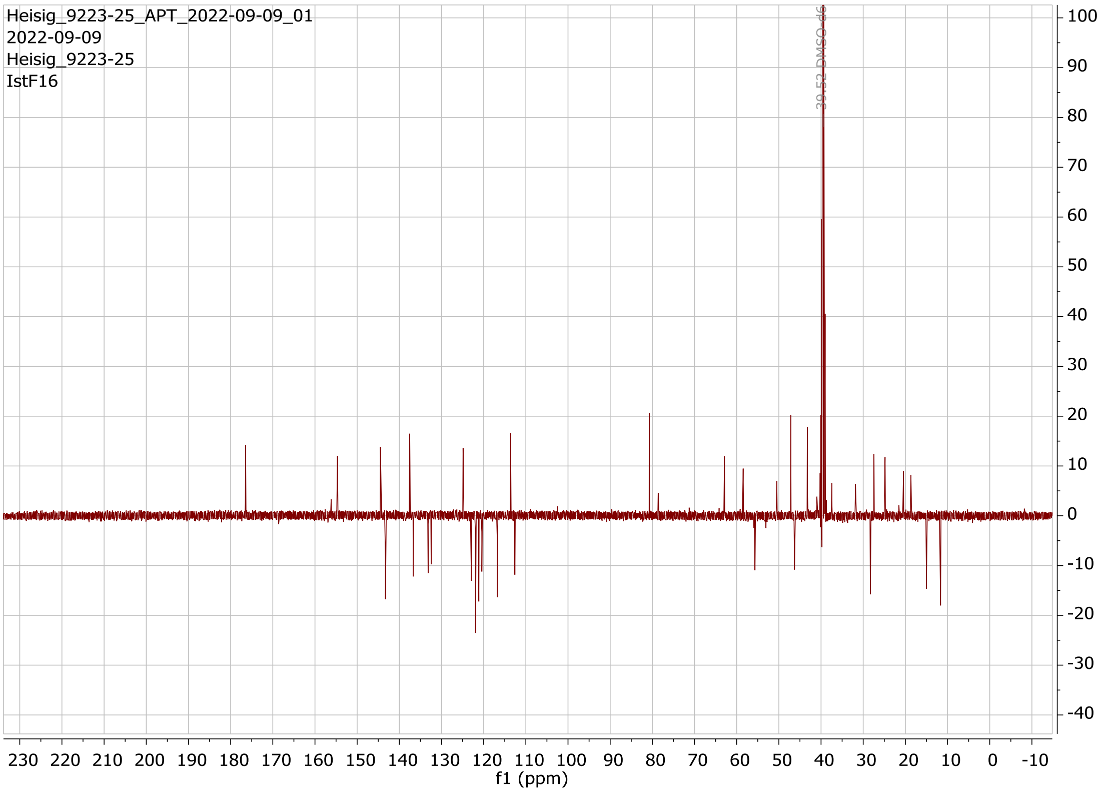

Spectra for 26

^{1}H NMR (400 MHz, DMSO-d_6)

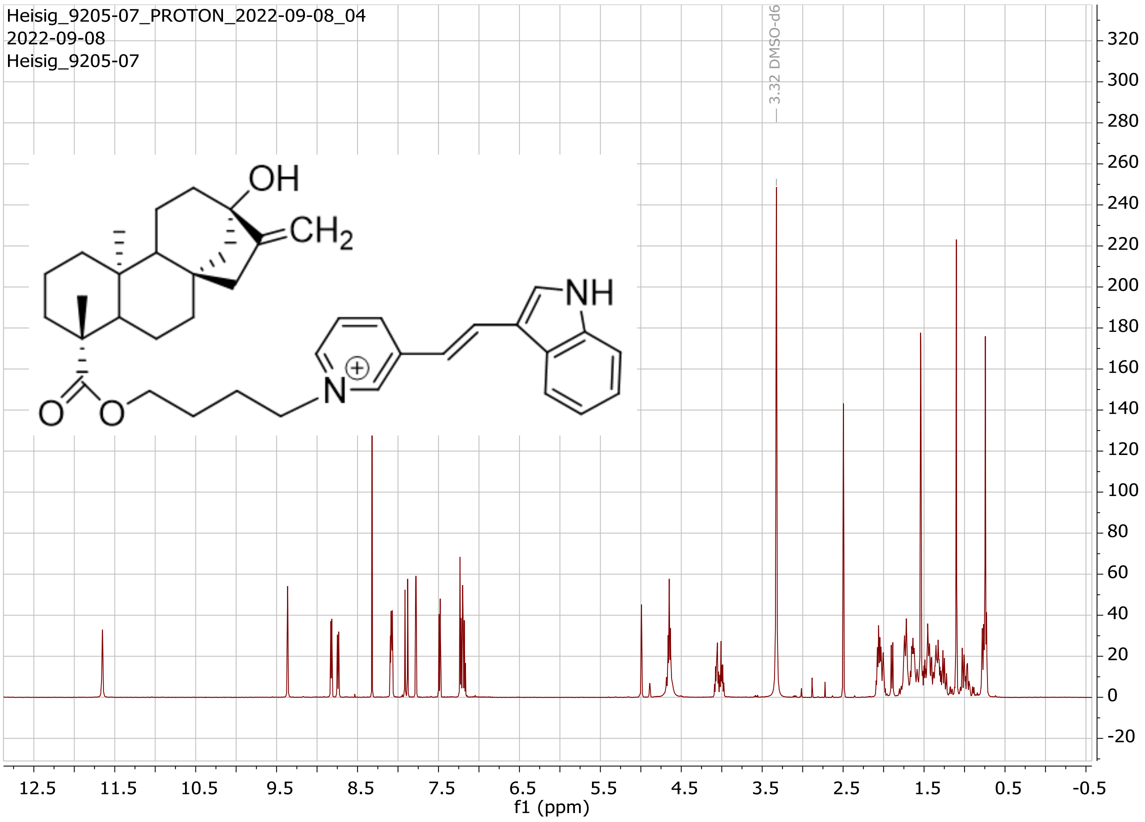

^{13}C NMR (APT, 101 MHz, DMSO-d_6)

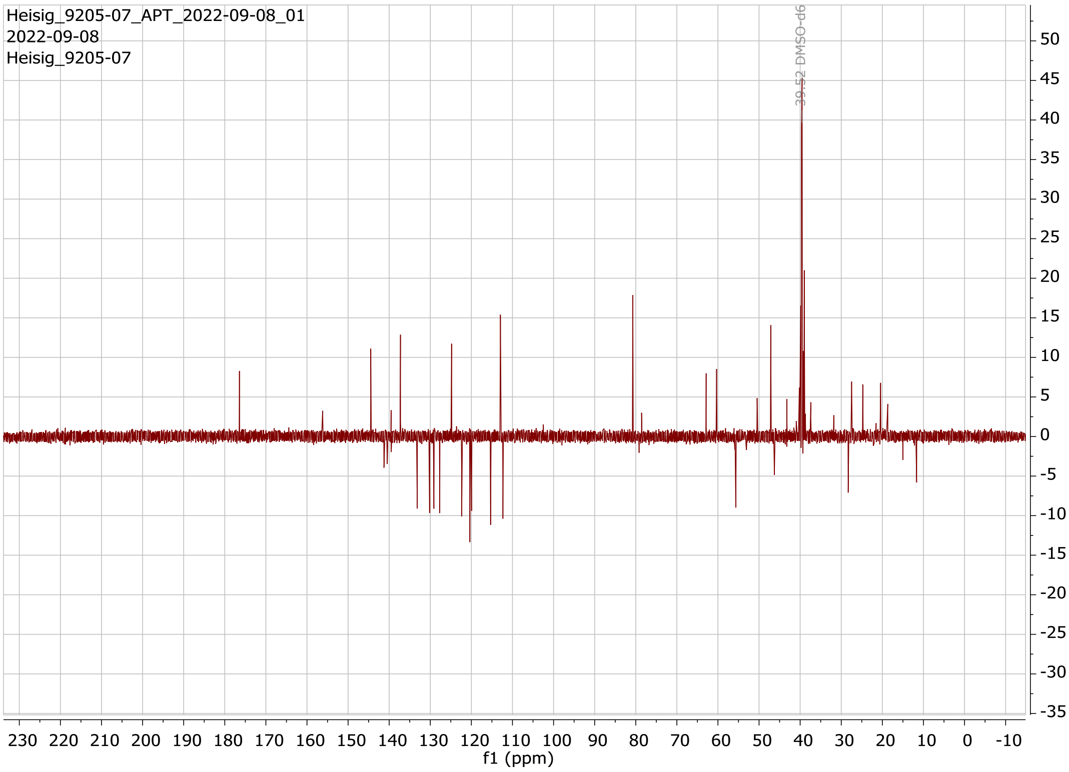

Supplement: Supplementary file 1 [file molecules-29-00381-s001.zip › molecules-2782098-supplementary.pdf]
